# Metabolism of plant-derived toxins from its insect host increases the success of the entomopathogenic fungus *Beauveria bassiana*

**DOI:** 10.1038/s41396-023-01480-3

**Published:** 2023-07-21

**Authors:** Ruo Sun, Benke Hong, Michael Reichelt, Katrin Luck, Duc Tam Mai, Xingcong Jiang, Jonathan Gershenzon, Daniel Giddings Vassão

**Affiliations:** 1 Max Planck Institute for Chemical Ecology, Department of Biochemistry, Jena, Germany; 2 Max Planck Institute for Chemical Ecology, Department of Natural Product Biosynthesis, Jena, Germany; 3 Max Planck Institute for Chemical Ecology, Department of Evolutionary Neuroethology, Jena, Germany; 4 Max Planck Institute of Geoanthropology, Department of Archaeology, Jena, Germany

**Keywords:** Metabolomics, Fungal ecology, Transcriptomics, Applied microbiology, Metabolomics

## Abstract

*Beauveria bassiana* is a soil fungus that parasitizes a large number of arthropod species, including numerous crop pests, causing white muscardine disease and is therefore used as a biological insecticide. However, some insects, such as the cabbage aphid (*Brevicoryne brassicae*), defend themselves chemically by sequestering dietary pro-toxins (glucosinolates) from their Brassicales host plants. Glucosinolates are accumulated by cabbage aphids and activated to form toxic isothiocyanates when under attack. While isothiocyanate formation protects aphids against most attackers, *B. bassiana* is still able to infect the cabbage aphid under natural conditions. We therefore investigated how this fungus is able to circumvent the chemical defense system of the cabbage aphid. Here, we describe how *B. bassiana* infection activates the cabbage aphid defense system, but the resulting toxins are metabolized by *B. bassiana* via the mercapturic acid pathway, of which the first step is catalyzed by glutathione-*S*-transferases of low substrate specificity. This detoxification pathway enhances *B. bassiana* growth when isothiocyanates are present in natural concentrations, and so appears to be an important factor in fungal parasitization of these chemically defended aphids.

## Introduction

Multitrophic interactions involving plants, herbivores, and herbivore enemies are ubiquitous in natural and agricultural ecosystems. In these contexts, plant chemical defenses are not only used by plants for protection against herbivores and pathogens, but also traverse trophic levels and can be exploited by some herbivores as defenses against their own predators, parasitoids, and pathogens [1, 2]. Many insect herbivores are now known to sequester plant defenses [3], but the effectiveness of these stored metabolites against different types of herbivore enemies has been much less studied, especially against pathogenic microorganisms.

Among the best-studied plant chemical defense systems are the glucosinolates (GSLs), glucosylated defense metabolites in plants of the order Brassicales that are activated by myrosinase enzymes upon tissue damage to produce isothiocyanates (ITCs) and other toxic hydrolysis products [4]. The GSL pro-toxins have side-chains derived from their precursor amino acids, and are accumulated in high concentrations in different plant tissues, often as mixtures. Their activation products are perceived by humans as the pungent flavors characteristic of, for example, cabbages, wasabi, mustard, and radishes. This two-component defense system, the so-called “mustard oil bomb”, generates defensive compounds that can traverse trophic levels, affecting not only consuming herbivores [5] but subsequently also their natural enemies [6, 7]. A well-known example involves the cabbage aphid *Brevicoryne brassicae* (Hemiptera: Aphididae), a specialist of Brassicales plants that sequesters plant GSLs. These insects ingest the plant pro-toxins via a piercing-sucking feeding mode that minimizes tissue rupture and so mostly circumvents GSL activation by the plant defense system [8]. Besides, these insects are capable of taking up and accumulating GLSs selectively [7]. In addition to sequestering GSLs in its hemolymph, the cabbage aphid produces an endogenous myrosinase enzyme in its head and thoracic muscles [9]. Once the cabbage aphid is attacked, its sequestered GSLs are activated by myrosinase just as when the plant is damaged, resulting in the formation of toxic ITCs and giving the cabbage aphid the apt moniker “walking mustard oil bomb” [9]. The ITCs thus formed by the herbivore can cause negative effects on higher trophic level predators or parasitoids [6, 7, 10, 11]. The high electrophilicity of the ITC –N = C = S functional group enables these compounds to react quickly and at times spontaneously with biological nucleophiles [12], resulting in their insecticidal [5], bactericidal [13, 14], and fungicidal properties [15]. In spite of these potent chemical defenses, populations of cabbage aphids can often be controlled by the use of suitable natural enemies such as insect predators [16], parasitoids [17], or entomopathogenic fungi [18]. However, it is not yet known how these enemies circumvent the ITCs released from the cabbage aphid.

The entomopathogenic fungus *Beauveria bassiana* (Hypocreales: Cordycipitaceae) is a biological control agent widely used against a number of agricultural insect pests such as whiteflies [19, 20], aphids [21, 22], and beetles [23, 24], in which it causes white muscardine disease. Once its conidia come into contact with the body of the insect host, they germinate, penetrate the cuticle, and grow inside the insects as hyphae and blastospores, typically killing the insect within days. Afterwards, a white mold emerges from the insect cadaver and produces new conidia for a next generation [25]. In terms of susceptibility to ITCs, previous studies of another entomopathogen, *Metarhizium anisopliae*, have shown that germination and growth in vitro, as well as its ability to infect insects were reduced by phenylethyl- (2PE-), 2-chlorophenyl-, and allyl-ITCs (A-ITC) [26]. In addition, the volatile A-ITC inhibited germination of conidia of the entomopathogenic fungi *B. bassiana* and *Isaria fumosorosea* [27]. Nevertheless, the ITC-producing cabbage aphid can still be readily controlled by *B. bassiana* and other entomopathogens [18, 28, 29]. It is unclear, however, whether the GSLs accumulated in the cabbage aphid are indeed activated and harmful to *B. bassiana* during their infection of this insect. Adding to the complexity of this interaction, the production and accumulation of different GSLs are variable among plants of Brassicales that serve as cabbage aphid hosts, as well as among different plant organs and plant developmental stages [30, 31]. Furthermore, the accumulation of GSLs from plants by the cabbage aphid is selective, being strongly influenced by the side-chain structure of the different GSLs [7, 10]. Nevertheless, whether GSLs with different side-chain structures have differential impact on *B. bassiana* after accumulation by the cabbage aphid is still unknown.

The ability of organisms to tolerate feeding on GSL-producing plants or insects can often be attributed to their capacities to metabolically deactivate GSLs or ITCs. Such metabolic mechanisms against the GSL defense system have been well studied in certain herbivorous insects. Larvae of *Plutella xylostella* and *Pieris rapae*, for example, can efficiently metabolize ingested GSLs to non-toxic products and thus prevent ITC formation [32, 33]. However, most herbivores are apparently unable to block ITC formation, but instead metabolize the ITCs once formed via conjugation to glutathione (GSH) followed by the mercapturic acid pathway [34]. Among microorganisms, the plant pathogens *Sclerotinia sclerotiorum* [35] and *Pseudomonas syringae* [36] have been shown to hydrolyze ITCs to form non-toxic amines, but entomopathogenic fungi have not been investigated regarding their metabolism of GSLs and ITCs.

Here we studied the accumulation, metabolism, and biological effects of these plant defensive metabolites in the tritrophic interaction between Brassicales plants, the cabbage aphid, and the entomopathogenic fungus *B. bassiana*. We examined how the parasitization by *B. bassiana* is affected by the GSLs accumulated as defenses by cabbage aphids. Using non-targeted and targeted metabolomic analyses, we identified the mercapturic acid pathway as the major route by which *B. bassiana* metabolizes ITCs during cabbage aphid infection. The fungal enzymes involved in the initial detoxification reaction were then identified and characterized in vitro. Finally, we investigated the influence of *B. bassiana* ITC metabolism on its physiological development. Our results help to shed light on how insect-sequestered and activated toxins can be tolerated by a widely used pest-controlling fungus, and illustrate more generally how plant defensive compounds act across multiple trophic levels, including effects on pathogenic microorganisms.

## Results

### Development of *B. bassiana* on cabbage aphids is impeded by glucosinolate (GSL) sequestration in the aphids

**Fig. 1:**
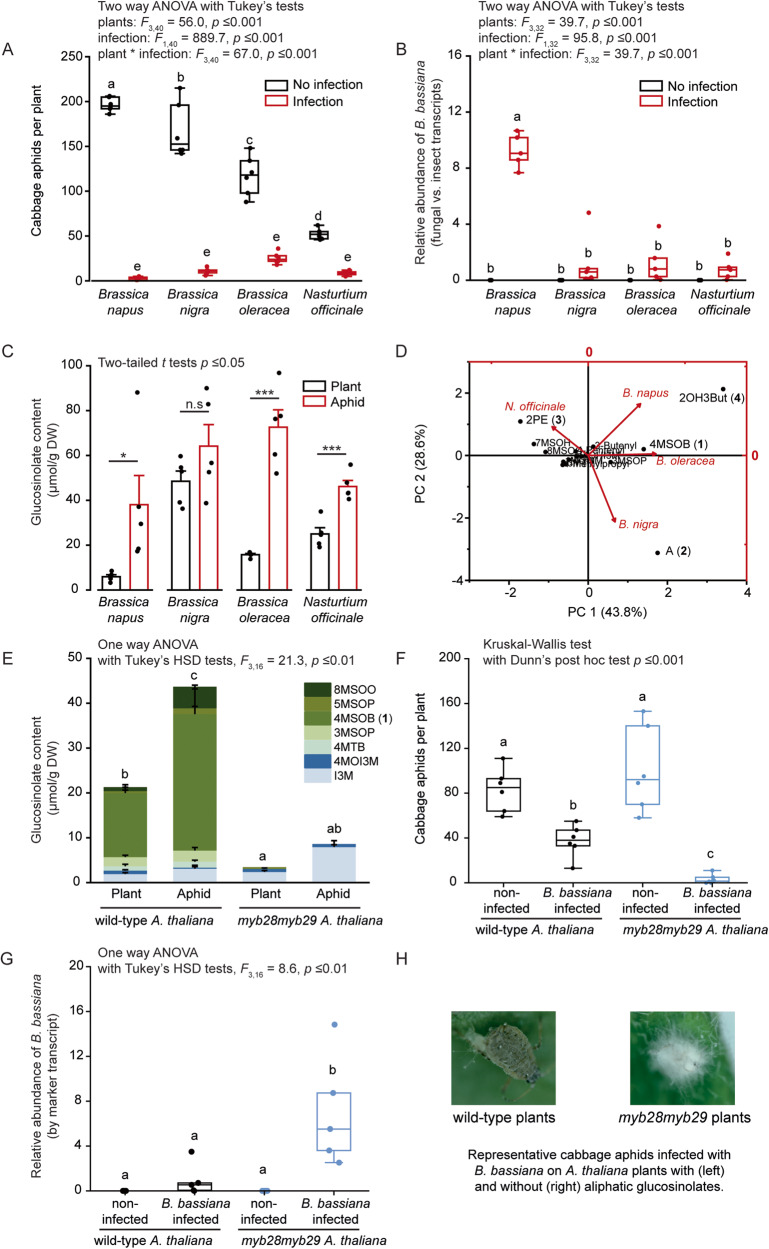
Development of *Beauveria bassiana* on cabbage aphids is affected by the glucosinolates (GSLs) of their Brassicales host plants. **A–D** Cabbage aphids were fed on *Brassica napus*, *B. nigra*, *B. oleracea*, or *Nasturtium officinale* as host plants. **A** Number of living aphids, including both surviving adults and their offspring, for aphids infected by *B. bassiana* compared to non-infected ones (*n* = 6). **B** Relative abundance of *B. bassiana* infecting aphids (measured as the transcript level of the *B. bassiana* actin gene (*actin*) relative to the cabbage aphid elongation factor 1 alpha gene (*ef1α*), *n* = 5). **C** GSL content of host plants and cabbage aphids (*n* = 5, detailed GSL contents are listed in Table S1). **D** Principal component analysis (PCA) plot showing the variation among GSL accumulation by the cabbage aphid on different Brassicales plants. Vectors indicate the direction and strength of the GSLs accumulated by aphids on different host plants in relation to the overall GSL distribution. **E–H** Cabbage aphids were fed on *Arabidopsis thaliana* with aliphatic GSLs (wild type Col-0 plants) and without aliphatic GSLs (*myb28myb29* mutant plants). **E** GSL content of *A. thaliana* plants and cabbage aphids (*n* = 5). **F** Number of living aphids after infection by *B. bassiana* (*n* = 6). **G** Relative abundance of *B. bassiana* infecting aphids (measured as the transcript level of the *B. bassiana actin* gene relative to the cabbage aphid *ef1α* gene, *n* = 5). **H** Photographs of *B. bassiana*-infected aphids from an *A. thaliana* wild-type plant (left) and a *myb28myb29* plant (right) taken on the 6th day after infection. 3MSOP-GSL, 3-methylsulfinylpropyl GSL; 4MSOB-GSL (**1**), 4-methylsulfinylbutyl GSL; 5MSOP-GSL, 5-methylsulfinylpentyl GSL; 8MSOO-GSL, 8-methylsulfinyloctyl GSL; 4MTB-GSL (**5**), 4-methylthiobutyl GSL; I3M-GSL, indolyl-3-methyl GSL; 4MOI3M-GSL, 4-methoxyindolyl-3-methyl GSL. The statistical methods are listed in the figures. Asterisks and lowercase letters denote statistically significant differences.

Experimental infection of the cabbage aphid (*B. brassicae*) by the entomopathogenic fungus *B. bassiana* dramatically reduced the size of the aphid populations. Aphids were fed for 14 d on *Brassica napus, Brassica nigra, Brassica oleracea*, or *Nasturtium officinale* plants (Fig. 1A), with each plant species containing a different major GSL: 2-hydroxy-3-butenyl (2OH3But)-GSL (**4**) in *B. napus*, allyl (A)-GSL (**2**) in *B. nigra*, 4-methylsulfinylbutyl (4MSOB)-GSL (**1**) in *B. oleracea*, and 2-phenylethyl (2PE)-GSL (**3**) in *N. officinale* (Table S1). Forty 4th instar nymphs were transferred to each individual host plant per replicate, with 6 replicates per plant species being infected by the fungus and another 6 remaining uninfected. During 5 days of feeding, the surviving 4th instar nymphs turned into adults and reproduced. The new offspring increased the total number of aphids, resulting in up to approximately 200 aphids per plant for the uninfected aphids on *B. napus* (Fig. 1A). However, the number of surviving *B. bassiana-*infected aphids, including offspring, was only approximately 3 aphids per plant for *B. napus*, 11 aphids per plant for *B. nigra*, 25 aphids per plant for *B. oleracea*, and 9 aphids per plant for *N. officinale* (Fig. 1A). The survivorship of infected aphids on *B. napus* was significantly lower than on *B. nigra* or *N. officinale* (Fig. S1B). The abundance of the fungus on aphids feeding on each host plant was then measured based on the quantities of the *B. bassiana actin (Bbactin)* gene transcript relative to the transcript of the cabbage aphid *elongation factor 1 alpha (ef1α)* gene, as determined by RT-qPCR on RNA extracted from infected and non-infected aphids (Fig. 1B). Aphids fed on *B. napus* plants contained over 7-fold higher *B. bassiana* levels than aphids fed on *B. nigra, B. oleracea, and N. officinale* plants (Fig. 1B). These results suggest that the insects fed on *B. napus* are less well defended against fungal infection.

As expected, the contents of GSLs accumulated in aphids were generally higher than those in their respective host plants (Fig. 1C and Table S1). The aphids accumulated 38.1 µmol GSLs per gram dry body weight when feeding on *B. napus*, while the total GSL concentrations were 64.1  µmol/g, 72.6 µmol/g, and 46.1 µmol/g in insects fed on *B. nigra, B. oleracea, and N. officinale*, respectively (Fig. 1C). The GSL profiles in the aphids broadly matched those of their corresponding host plants, although their concentrations in the insects relative to those in the plant leaves varied, with the aphids containing 15.3 times the concentration of 2OH3But-GSL (**4**) (*B. napus*), 1.2 times A-GSL (**2**) (*B. nigra*), 6.2 times 4MSOB-GSL (**1**) (*B. oleracea*), and 1.0 times 2PE-GSL (**3**) (*N. officinale*), relative to their respective plant hosts (Fig. 1D and Table S1). Taken together, these results indicate that the successful parasitization of cabbage aphids by *B. bassiana* is correlated not only to the total amount of GSLs accumulated by the insects, but also to the chemical structure of the accumulated GSLs.

To examine the influence of the accumulated GSLs on the resistance of the cabbage aphid against *B. bassiana* in a more controlled way, *Arabidopsis thaliana* plants with (Col-0 wild-type) and without (*myb28myb29* mutant) aliphatic GSLs were used as hosts for the cabbage aphid. Aphids accumulated aliphatic GSLs after two weeks of continuous feeding on wild-type plants, especially 4MSOB-GSL (**1**) which reached 30.4 µmol/g DW, but not when feeding on *myb28myb29* plants, which lack this class of GSLs (Fig. 1E). When infected by *B. bassiana*, aphids that had fed on wild-type plants showed increased survival and offspring compared to aphids that had fed on *myb28myb29* plants (Fig. 1F and Fig. S1C, D). Correspondingly, the relative abundance of *Bbactin* transcripts was lower in cabbage aphids feeding on wild-type plants than on GSL-depleted plants (Fig. 1G). Photographs of cabbage aphids taken five days after *B. bassiana* infection show the hyphae emerging from aphid cadavers and visible as a white mold when aphids had fed on *myb28myb29* plants (Fig. 1H). However, the cadavers of *B. bassiana*-infected aphids that had been fed on wild-type Col-0 plants containing aliphatic GSLs did not display such obvious emerged hyphae (Fig. 1H). Therefore, when cabbage aphids fed on wild-type Col-0 plants, they accumulated higher amounts of aliphatic GSLs and showed increased survival and offspring after *B. bassiana* infection, with correspondingly lower *B. bassiana* abundance compared to aphids fed on plants lacking aliphatic GSLs.

### GSLs sequestered by the cabbage aphid are hydrolyzed to isothiocyanates (ITCs) upon *B. bassiana* infection

**Fig. 2:**
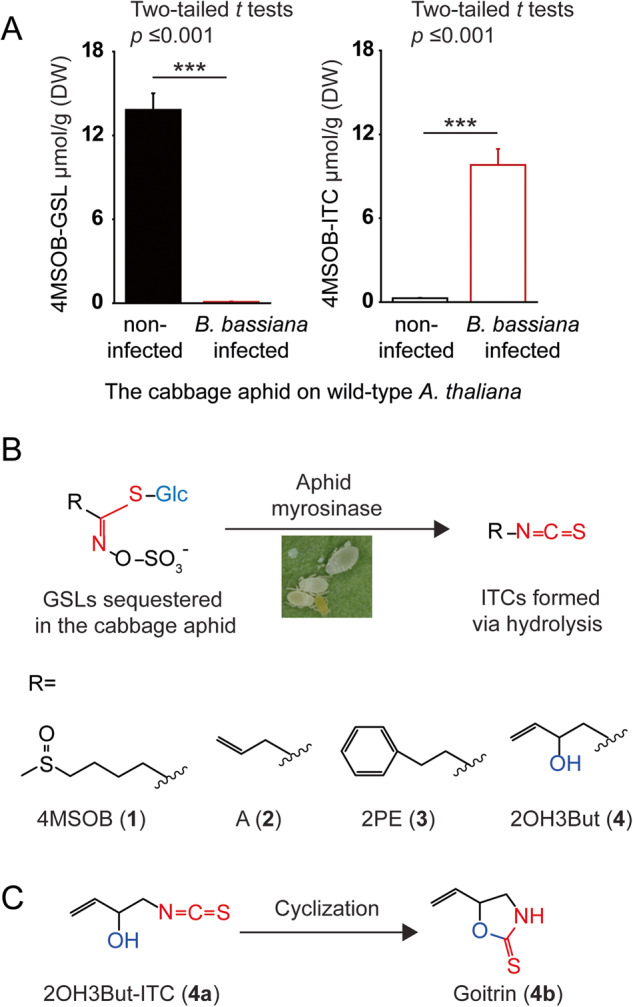
Formation of GSL hydrolysis products in the cabbage aphid. **A** Cabbage aphids feeding on wild-type *A. thaliana* plants (which contain 4MSOB-GSL (**1**)) were infected with *B. bassiana* (*n* = 5). Amounts of 4MSOB-GSL (**1**) (left) and its hydrolysis product 4MSOB-ITC (**1a**) (right) were quantified. **B** The major GSLs sequestered in cabbage aphids were hydrolyzed to ITCs upon *B. bassiana* infection. **C** 2OH3But-ITC (**4a**) cyclizes spontaneously to form goitrin (**4b**). Statistically significant differences between means (±SE) were determined by two-tailed *t* tests in A and are denoted by asterisks.

It has been well documented that cabbage aphids utilize accumulated GSLs and their toxic ITC derivatives as defenses when attacked by predators [7, 10], but there is no information on whether GSLs can be deployed against pathogens. In aphids not infected by *B. bassiana*, 4MSOB-GSL (**1**) was mostly present as the intact GSL (Fig. 2A). Once cabbage aphids were infected by *B. bassiana*, 4MSOB-GSL (**1**) levels were drastically reduced in the aphids feeding on wild-type *A. thaliana* plants, and most of the sequestered 4MSOB-GSL (**1**) was converted to 4MSOB-ITC (**1a**) (Fig. 2A). Moreover, other GSLs such as A-GSL (**2**), 2PE-GSL (**3**), and 2OH3But-GSL (**4**) that were sequestered by cabbage aphids from other Brassicales host plants were also hydrolyzed and transformed to ITCs and other hydrolysis products, such as A-ITC (**2a**), 2PE-ITC (**3a**), and goitrin (**4b**), respectively (Fig. 2B). Goitrin (**4b**) is the spontaneous cyclization product of 2OH3But-ITC (**4a**) due to the intramolecular reaction of its hydroxyl group with the electrophilic ITC core group (Fig. 2C) [30]. This variation in GLS hydrolysis products may affect *B. bassiana* development on aphids fed on *B. napus*, which accumulate high amounts of 2OH3But-GSL (**4**) from their host plant (Fig. 1C and Table S1). We then measured the potential of the aphid to carry out GSL hydrolysis, and found that 1.5 to 3.8 µmol 4MSOB-ITC (**1a**), A-ITC (**2a**), 2PE-ITC (**3a**), and goitrin (**4b**) were formed per min during in vitro incubation of the corresponding GSLs with 1 µg crude protein extracted from the cabbage aphid (Fig. S2). Hence, these data indicate that during infection of the cabbage aphid, *B. bassiana* encounters ITCs produced by activation of stored GSLs, and so may benefit from detoxifying these toxins.

### *B. bassiana* metabolizes ITCs via the mercapturic acid pathway

**Fig. 3:**
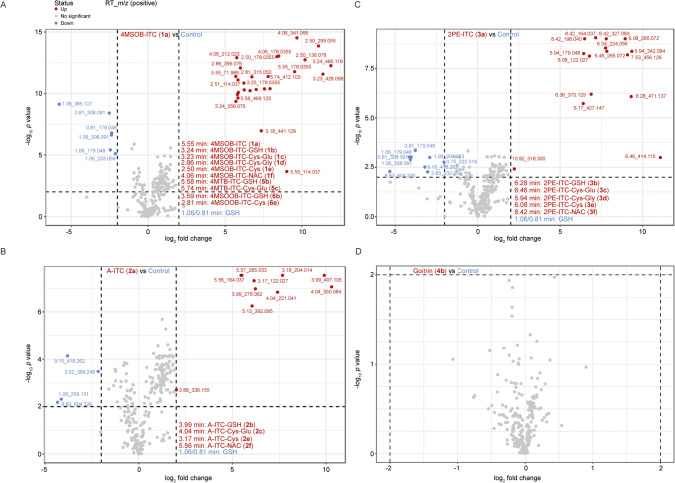
*B. bassiana* metabolizes the major GSL hydrolysis products, the ITCs, to form glutathione (GSH) conjugates and derivatives. **A** Volcano plots of extracted LC-MS/MS features from non-targeted UHPLC-qTOFMS analyses after incubation of the fungus with 4MSOB-ITC (**1a**) in potato dextrose broth indicate that the major metabolites detected derive from conjugation to glutathione (GSH), followed by cleavage of the amino acid constituents of GSH to form CysGlu, CysGly, and Cys conjugates, and then acetylation to give the corresponding NAC conjugate (for chemical structures, please see Fig. 4). Metabolites of 4MTB-ITC (**5a**) and 4MSOOB-ITC (**6a**), which make up 3.8% of a commercial standard of 4MSOB-ITC (**1a**) (Fig. S3), were also detected. As a control (CT), the fungus was incubated without 4MSOB-ITC (**1a**). **B** A-ITC (**2a**) and **C** 2PE-ITC (**3a**) were also metabolized by *B. bassiana* with the formation of apparent GSH conjugates and derivatives. **D** No detectable derivatives were formed when *B. bassiana* was exposed to goitrin (**4b**). Confirmed and putative products are listed in Table S4. The NMR analyses of ITC conjugates are shown in Supplementary file 2; the chromatography of commercial ITC conjugates and mass spectra of the putative ITC-amino acid conjugates are shown in Supplementary file 4. Significant differences between means were determined by two sample *t* tests (*p* ≤0.01).

To determine whether *B. bassiana* metabolizes the major GSL hydrolysis products it encounters, *B. bassiana* cultures were incubated with either 4MSOB-ITC (**1a**), A-ITC (**2a**), 2PE-ITC (**3a**) or goitrin (**4b**) dissolved in 0.025% ethanol in potato dextrose broth (PDB) for 24 h, and the resulting metabolites were analyzed via non-targeted metabolomics using ultra high performance liquid chromatography coupled to quadrupole time of flight mass spectrometry (UHPLC-qTOFMS). *B. bassiana* incubated with 0.025% ethanol in PDB was used as a negative control for metabolomic comparisons. Volcano plots of extracted LC-MS/MS features from these non-targeted analyses indicated that the major metabolites of 4MSOB-ITC (**1a**) produced by *B. bassiana* were 4MSOB-ITC-GSH (**1b**) (the glutathione conjugate), 4MSOB-ITC-Cys-Glu (**1c**) (the cysteinyl-glutamate conjugate), 4MSOB-ITC-Cys-Gly (**1d**) (the cysteinyl-glycine conjugate), 4MSOB-ITC-Cys (**1e**) (the cysteine conjugate), and 4MSOB-ITC-NAC (**1f**) (the *N*-acetyl-cysteine conjugate) (Fig. 3A). Identification was confirmed by chromatography of standards prepared by chemical synthesis. Chromatographic peaks with MS features matching some of the corresponding metabolites of 4MTB (4-methylthiobutyl)-ITC (**5a**) and 4MSOOB (4-methylsulfonylbutyl)-ITC (**6a**) (which make around 3.8% of the commercially obtained 4MSOB-ITC (**1a**) used herein (Fig. S3)), were also detected, including 4MTB-ITC-GSH (**5b**), 4MTB-ITC-Cys-Glu (**5c**), 4MSOOB-GSH (**6b**), and 4MSOOB-Cys (**6e**) (Fig. 3A). Similarly, A-ITC-GSH (**2b**), A-ITC-Cys-Glu (**2c**), A-ITC-Cys (**2e**), and A-ITC-NAC (**2f**) conjugates were detected as products of A-ITC (**2a**) metabolism (Fig. 3B); and 2PE-ITC-GSH (**3b**), 2PE-ITC-Cys-Glu (**3c**), 2PE-ITC-Cys-Gly (**3d**), 2PE-ITC-Cys (**3e**), and 2PE-ITC-NAC (**3f**) conjugates were the main products of 2PE-ITC (**3a**) metabolism by *B. bassiana* (Fig. 3C). However, upon incubation with goitrin (**4b**), no signals corresponding to a potential GSH conjugate or its derivatives could be observed, and no signals were present that suggested goitrin (**4b**) metabolism (Fig. 3D). The structures of the ITC conjugate products were confirmed by chemical synthesis followed by NMR analyses (Supplementary file 2), or comparison to commercially available standards.

**Fig. 4:**
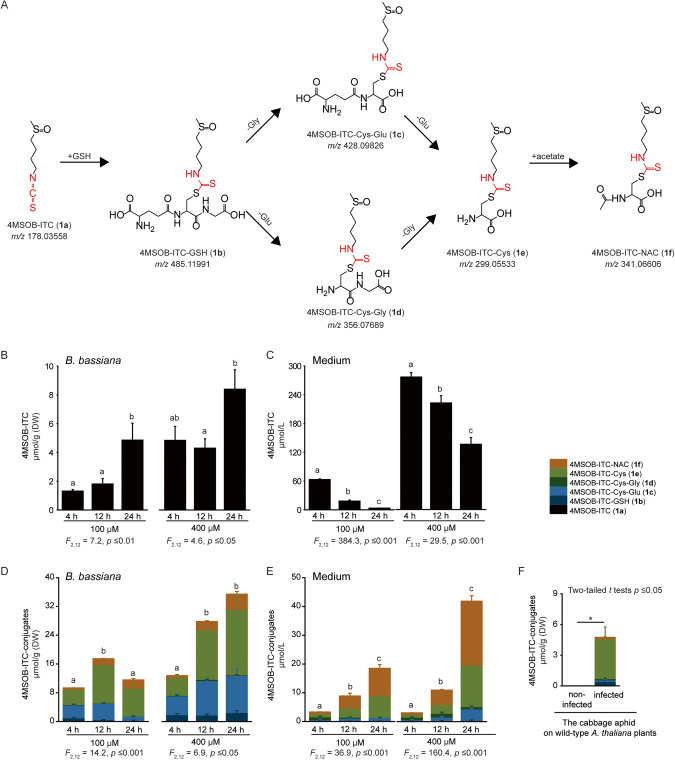
4MSOB-ITC (1a) is metabolized by *B. bassiana* via the mercapturic acid pathway. **A** An overview of the pathway for 4MSOB-ITC (**1a**) metabolism. **B–E** 4MSOB-ITC (**1a**) was added to the *B. bassiana* growth medium (100 and 400 µM) and analyses were performed after 4, 12, and 24 h of incubation (*n* = 5). Amounts of 4MSOB-ITC (**1a**) remaining in *B. bassiana* (**B**) and its growth medium (**C**). 4MSOB-ITC conjugates found in *B. bassiana* (**D**) and its growth medium (**E**). **F** 4MSOB-ITC conjugates formed in cabbage aphids feeding on wild-type *A. thaliana* plants (which contain 4MSOB-GSL (**1**)) after infection with *B. bassiana* (*n* = 5). Statistically significant differences between means (±SE) were determined by Tukey HSD tests in conjunction with one-way ANOVA in **B**–**E**, and by two-tailed *t* tests in **F**, and are denoted by asterisks and lowercase letters.

*B. bassiana* appears to metabolize most ITCs via the general mercapturic acid pathway. This pathway involves the conjugation of the toxin to glutathione (GSH), followed by sequential hydrolysis of the amino acid constituents of GSH to form Cys-Glu, Cys-Gly, and Cys conjugates, and finally acetylation to give the NAC conjugate (Fig. 4A and Fig. S4). To examine the dynamics of 4MSOB-ITC (**1a**) metabolism by *B. bassiana* in more detail, fungal cultures were incubated with 100 µM or 400 µM 4MSOB-ITC (**1a**) in PDB medium for 4 h, 12 h, and 24 h, and 4MSOB-ITC (**1a**) metabolites were analyzed via targeted metabolomics using UHPLC coupled to a triple-quadrupole MS. *B. bassiana* absorbed 4MSOB-ITC (**1a**) from the PDB medium into their hyphae and blastospores, and the concentrations of this compound in *B. bassiana* increased during the 24 h incubation (Fig. 4B). Conversely, the amounts of 4MSOB-ITC (**1a**) present in the surrounding PDB medium significantly decreased during this time and 4MSOB-ITC (**1a**) was barely detectable in the 100 µM samples after 24 h (Fig. 4C). Confirming the previous results, *B. bassiana* metabolized the absorbed 4MSOB-ITC (**1a**) via GSH conjugation followed by the mercapturic acid pathway, and 4MSOB-ITC conjugates were increasingly observed both in *B. bassiana* tissues and in the surrounding PDB medium (Fig. 4D, E). In *B. bassiana* hyphae and blastospores, 4MSOB-ITC-Cys-Glu (**1c**) and 4MSOB-ITC-Cys (**1e**) represented over 70% of the 4MSOB-ITC conjugates formed (Fig. 4D), while 4MSOB-ITC-Cys (**1e**) and 4MSOB-ITC-NAC (**1f**) comprised over 85% of the total 4MSOB-ITC conjugates present in the medium after 24 h (Fig. 4E). Furthermore, we analyzed *B. bassiana*-infected aphids fed on *A. thaliana* wild type plants, and observed that one third of the 4MSOB-ITC (**1a**) formed was converted into 4MSOB-ITC conjugates, mainly in the form of 4MSOB-ITC-Cys (**1e**), after five days of *B. bassiana* infection (Fig. 2A and Fig. 4F).

### *Beauveria bassiana* glutathione-*S*-transferases (GSTs) catalyze reactions with ITCs, and expression of some encoding genes is induced by ITC treatment

**Fig. 5:**
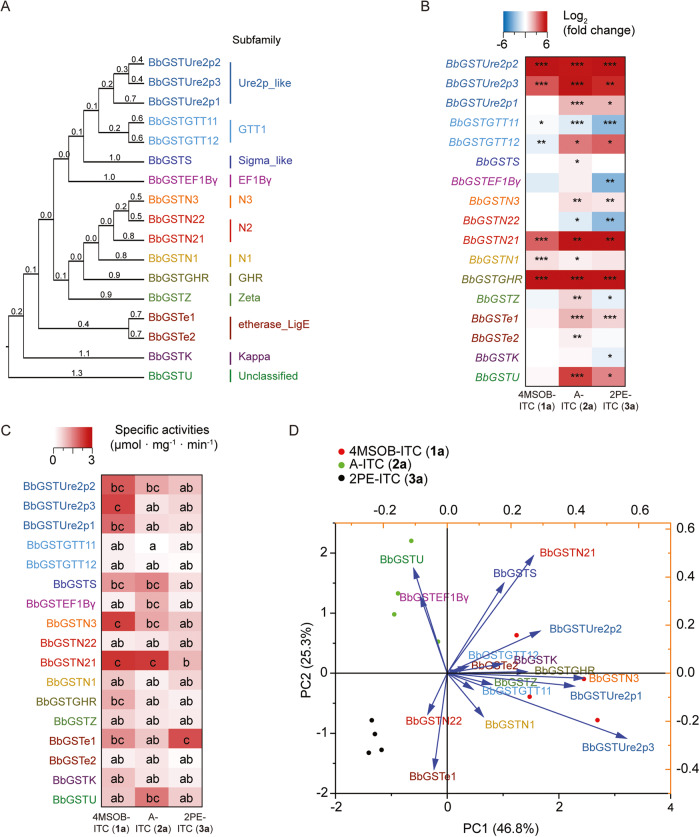
Glutathione-*S*-transferases (GSTs) of *B. bassiana* and their gene inducibility and enzymatic activities with three ITCs. **A** Phylogenetic analysis of the seventeen *B. bassiana* GSTs shows division into twelve groups, eight of which represent existing subfamilies. The BbGST amino acid sequences were aligned and a UPGMA tree was generated. The branch labels represent the expected amino acid substitutions per site. **B** Heatmap showing the expression of GST-encoding genes in *B. bassiana* (relative to *Bbactin*, *n* = 5). The data show log_2_ fold-change of *GST* gene expression level in *B. bassiana* incubated with 4MSOB-ITC (**1a**), A-ITC (**2a**), and 2PE-ITC (**3a**), relative to the expression in the *B. bassiana* control group. Only statistically significant differences are indicated. **C** Specific activity ±SE (µmol substrate consumed · mg^−1^ enzyme · min^−1^) of *B. bassiana* GSTs was determined for 4MSOB-ITC (**1a**), A-ITC (**2a**), and 2PE-ITC (**3a**) (*n* = 4). Specific activity values corrected for non-enzymatic conjugation are listed in Table S5. **D** PCA plot showing the variation of GST activities with different ITCs. Vectors indicate the direction and strength of each GST protein activity relative to the overall distribution. Statistically significant differences between means (±SE) were determined by two-tailed *t* tests (*p* ≤0.05) in **B**, and by Tukey HSD tests in conjunction with two-way ANOVA in **C**, and are denoted by asterisks and lowercase letters.

Given the predominance of mercapturic acid pathway products after exposure to ITCs, the biochemistry of *B. bassiana* ITC metabolism was investigated in more detail. Glutathione-*S*-transferases (GSTs) are typically responsible for the first metabolic step of GSH conjugation to ITCs and other electrophiles. To elucidate the involvement of particular GSTs, all seventeen putative GST-encoding genes present in *B. bassiana* were cloned. Predicted BbGST proteins were classified into twelve subfamilies based on conserved domains within the amino acid sequence. Proteins were assigned to the Ure2p_like, GTT1, Sigma_like, EF1Bγ, N_3 (GSTN family, unknown subfamily 3), N_2 (GSTN family, unknown subfamily 2), N_1 (GSTN family, unknown subfamily 1), glutathionyl hydroquinone reductase (GHR), Zeta, etherase_LigE, and Kappa subfamilies as well as to a group of unclassified GSTs. They were named as BbGSTUre2p, BbGSTGTT1, BbGSTS, BbGSTEF1Bγ, BbGSTN3, BbGSTN2, BbGSTN1, BbGSTGHR, BbGSTZ, BbGSTe, BbGSTK, and BbGSTU (Fig. 5A and Fig. S5). The closest hits of each BbGST sequence among previously classified GST family proteins in the NCBI database are listed in Table S2.

Levels of *BbGST* expression in *B. bassiana* incubated with 50 µM ITCs (4MSOB-ITC (**1a**), A-ITC (**2a**), and 2PE-ITC (**3a**)) for 4 h were compared to those from a control treatment using RT-qPCR. Consequently, we observed that expression of *BbGSTUre2p2*, *BbGSTUre2p3*, *BbGSTN21*, and *BbGSTGHR* was upregulated by all three ITCs (Fig. 5B). The expression of *BbGSTUre2p1*, *BbGSTGTT12*, *BbGSTN3*, *BbGSTe1*, and *BbGSTU* was induced by A-ITC (**2a**) and 2PE-ITC (**3a**), but not by 50 µM 4MSOB-ITC (**1a**) (Fig. 5B). When *B. bassiana* was incubated with 400 µM 4MSOB-ITC (**1a**), the expression of *BbGSTU* was induced after 4 h of incubation, and *BbGSTe1* and *BbGSTN1* transcripts were up-regulated after 12 h of incubation (Fig. S6). The results show that the expression of *BbGST*s from eight subfamilies, namely *BbGSTUre2p*, *BbGSTGTT1*, *BbGSTN3*, *BbGSTN2*, *BbGSTN1*, *BbGSTGHR*, *BbGSTe*, and *BbGSTU*, is induced by ITCs (Fig. 5B), and the inducibility of *BbGST* genes is influenced by both the side-chain structure of ITCs and the concentration of these toxins.

The enzyme activities of BbGSTs towards 4MSOB-ITC (**1a**), A-ITC (**2a**), and 2PE-ITC (**3a**) were measured using in vitro incubations of His-tag purified heterologously produced enzymes, with the corresponding GSH-conjugate products being quantified by their absorption at 274 nm [37]. Although the BbGSTs characterized were distributed into twelve subfamilies and the amino acid identities among the proteins were very low (Fig. 5A), varying between 6.5 to ~40% (Table S3), the most active BbGSTs all had higher rates of catalysis with 4MSOB-ITC (**1a**) than A-ITC (**2a**) or 2PE-ITC (**3a**), including BbGSTUre2p1, BbGSTUre2p2, BbGSTUre2p3, BbGSTN21, and BbGSTN3 (Fig. 5C). BbGSTU had higher activity with A-ITC (**2a**), and BbGSTe1 had higher activity with 2PE-ITC (**3a**) (Fig. 5C). Based on a principal component analysis among GST protein activities and ITCs, it was apparent that BbGSTUre2p1, BbGSTUre2p3, and BbGSTN3 were the most specific enzymes towards 4MSOB-ITC (**1a**); BbGSTU was more specific to A-ITC (**2a**); and BbGSTe1 was more specific to 2PE-ITC (**3a**) (Fig. 5D). In vitro enzyme kinetic assays confirmed that BbGSTUre2p2, BbGSTUre2p3, and BbGSTe1 had higher activities (*V*_max_) with 4MSOB-ITC (**1a**) than A-ITC (**2a**) and 2PE-ITC (**3a**) (Table 1). BbGSTU, BbGSTN21, and BbGSTN3 had the highest catalytic efficiencies (*k*_cat_/*K*_M_) with A-ITC (**2a**). BbGSTe1 had the highest *V*_max_ and *k*_cat_/*K*_M_ with 2PE-ITC (**3a**) of any of the enzymes, although its *k*_cat_/*K*_M_ with 4MSOB-ITC (**1a**) was still higher than that for 2PE-ITC (**3a**) (Table 1). Therefore, we could confirm that BbGSTs from different families are active in vitro towards ITCs with different side-chain structures, and might cooperate in vivo to metabolize these plant-derived, insect-formed toxins.

**Table 1:** Kinetic constants of *B. bassiana* GST enzymes with the substrates 4MSOB-ITC (**1a**), A-ITC (**2a**) and 2PE-ITC (**3a**).

Enzymes	Substrates	*V*_max_	*K*_M_	*k*_cat_	*k*_cat_/*K*_M_
		µmol · mg^-1^ · min^-1^	µM	s^-1^	mM^-1^ · s^-1^
BbGSTUre2p2	4MSOB-ITC (**1a**)	1.09 ± 0.54	1210.99 ± 889.50	0.45 ± 0.23	0.37 ± 0.19
	A-ITC (**2a**)	0.44 ± 0.12	487.23 ± 266.11	0.18 ± 0.05	0.37 ± 0.10
	2PE-ITC (**3a**)	0.15 ± 0.02	223.06 ± 86.81	0.06 ± 0.01	0.28 ± 0.05
BbGSTUre2p3	4MSOB-ITC (**1a**)	1.04 ± 0.27	747.36 ± 338.25	0.47 ± 0.12	0.63 ± 0.17
	A-ITC (**2a**)	0.59 ± 0.24	831.61 ± 572.57	0.27 ± 0.11	0.32 ± 0.13
	2PE-ITC (**3a**)	0.08 ± 0.01	71.76 ± 38.91	0.04 ± 0.01	0.49 ± 0.08
BbGSTU	4MSOB-ITC (**1a**)	0.40 ± 0.09	365.55 ± 184.07	0.24 ± 0.05	0.67 ± 0.15
	A-ITC (**2a**)	2.64 ± 1.33	1178.31 ± 884.78	1.59 ± 0.80	1.35 ± 0.68
	2PE-ITC (**3a**)	0.44 ± 0.09	391.89 ± 159.63	0.27 ± 0.06	0.68 ± 0.14
BbGSTe1	4MSOB-ITC (**1a**)	3.86 ± 1.80	1220.41 ± 839.36	1.77 ± 0.82	1.45 ± 0.68
	A-ITC (**2a**)	0.41 ± 0.05	312.75 ± 100.06	0.19 ± 0.02	0.59 ± 0.08
	2PE-ITC (**3a**)	1.90 ± 0.76	860.76 ± 561.56	0.88 ± 0.35	1.02 ± 0.41
BbGSTN21	4MSOB-ITC (**1a**)	3.96 ± 1.34	1132.00 ± 578.29	1.66 ± 0.56	1.47 ± 0.50
	A-ITC (**2a**)	2.39 ± 0.58	448.48 ± 226.89	1.00 ± 0.24	2.23 ± 0.54
	2PE-ITC (**3a**)	0.97 ± 0.86	1191.89 ± 1464.92	0.41 ± 0.36	0.34 ± 0.30
BbGSTN3	4MSOB-ITC (**1a**)	1.06 ± 0.30	884.72 ± 412.37	0.72 ± 0.21	0.82 ± 0.23
	A-ITC (**2a**)	1.61 ± 0.78	784.38 ± 647.72	1.09 ± 0.53	1.39 ± 0.68
	2PE-ITC (**3a**)	0.32 ± 0.11	396.52 ± 270.03	0.22 ± 0.08	0.55 ± 0.19

Michaelis-Menten constants (±SE) were determined by nonlinear regression of enzymatic activities measured using variable ITC concentrations (25 µM to 800 µM) at saturating GSH concentration (4 mM), *n* = 4.

### ITCs reduce *B. bassiana* growth at high concentration because of insufficient glutathione (GSH) for GST-catalyzed detoxification reactions

**Fig. 6:**
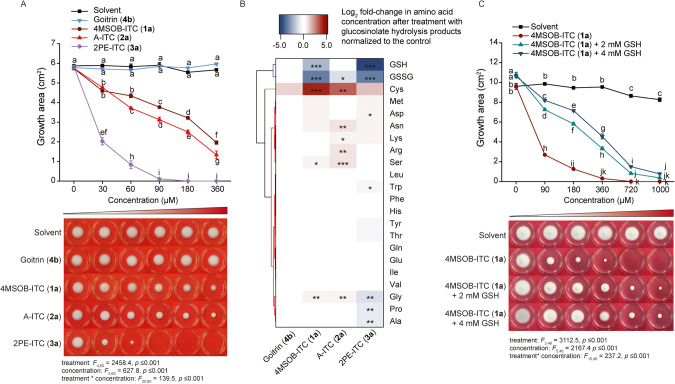
GSL hydrolysis products reduce *B. bassiana* growth and GSH content, but supplementation with GSH alleviates the toxicity of 4MSOB-ITC (1a). **A** The growth of *B. bassiana* is negatively correlated with the concentration of ITCs on PDA plates (*n* = 3). **B** Amino acid contents of *B. bassiana* incubated with GSL hydrolysis products. Data show the log_2_ fold-change of amino acid concentrations after hydrolysis product treatments, normalized to the control (*n* = 4). **C** The inhibition of *B. bassiana* growth on basic medium with increasing concentrations of 4MSOB-ITC (**1a**) is reduced by 2 mM and 4 mM added GSH (*n* = 3). Statistically significant differences between means (±SE) were determined by Tukey HSD tests in conjunction with two-way ANOVA in **A** and **C**, and with one-way ANOVA in **B**, and are denoted by asterisks and lowercase letters.

Although *B. bassiana* detoxifies ITCs via conjugation to GSH, the growth of this fungus was still negatively impacted by these GSL hydrolysis products. The growth of *B. bassiana* was negatively correlated with the concentrations of 4MSOB-ITC (**1a**), A-ITC (**2a**), and 2PE-ITC (**3a**) on potato dextrose agar (PDA) plates, but was not affected by the goitrin (**4b**) treatment (Fig. 6A). Additionally, *B. bassiana* grew much better on media containing 4MSOB-ITC (**1a**) and A-ITC (**2a**) than on media with 2PE-ITC (**3a**). *B. bassiana* growth was reduced by 50% at 4MSOB-ITC (**1a**) and A-ITC (**2a**) concentrations around 180 µM, while 2PE-ITC (**3a**) reduced *B. bassiana* growth by two-thirds even at 30 µM (Fig. 6A). In order to understand the biochemical reasons for these growth reductions, we examined the amounts of free amino acids in *B. bassiana*, as nitrogen is often a limiting macronutrient for fungal growth [38]. The non-targeted metabolomic analyses had shown that GSH concentrations declined after incubation with ITCs (Fig. 3). Using targeted chemical analyses, we determined that ITC metabolism reduced not only GSH content, but also that of oxidized glutathione (GSSG) (Fig. 6B). However, cysteine and glycine concentrations increased after 4MSOB-ITC (**1a**) and A-ITC (**2a**) treatment, but not after exposure to 2PE-ITC (**3a**), which reduced the contents of not only glycine, but also proline, alanine, and tryptophan, compared to the untreated control group (Fig. 6B).

In order to further explore the connection between the decline of GSH levels and the reduction of fungal growth, we complemented the medium with GSH. The radial growth of *B. bassiana* on the basic medium was increased by the supplementation of the medium with increasing concentrations of GSH, which partially remedied the toxicity of 4MSOB-ITC (**1a**) towards *B. bassiana* (Fig. 6C). Similarly, an increased availability of GSH partially rescued *B. bassiana* growth under A-ITC (**2a**) exposure, but only helped the fungus very slightly in the presence of 2PE-ITC (**3a**) (Fig. S7). Therefore, BbGSTs metabolize ITCs but at the marked expense of GSH, whose concentration appears to limit the extent of detoxification possible for *B. bassiana* under natural ITC concentrations.

## Discussion

Plant chemical defenses are sometimes recruited by specialized insect herbivores for protection against their enemies [2, 9, 39–42], but the ingestion and accumulation of plant-produced toxins has the potential to negatively impact these herbivores as well. Insects can avoid poisoning themselves by being insensitive to plant toxins [43], or sequestering non-toxic precursors (“pro-toxins”) [9, 40, 44, 45] that can be secreted or activated when needed. The cabbage aphid *B. brassicae* [9] and the horseradish flea beetle *Phyllotreta armoraciae* [45] are two insect herbivores specialized on crucifer plants that can sequester and accumulate ingested GSLs in their hemolymph. Both produce endogenous myrosinases to activate GSL, forming “walking and flying mustard oil bombs” that may be effective against higher trophic level organisms such as the predatory insects *Harmonia axyridis* [11], *Chrysoperla carnea* [7], and *Adalia bipunctata* [16]. Nothing is known, however, about how this defense might function against insect pathogens. In this study, we examine whether the GSLs selectively accumulated by the cabbage aphid affect the development of *B. bassiana*, an entomopathogenic fungus often used in pest control. Using *A. thaliana* wild-type plants and *myb28myb29* mutant plants (with and without aliphatic GSLs, respectively), we determined that aliphatic GSLs negatively impact *B. bassiana* (Fig. 1G, H). Cabbage aphids feeding on *B. nigra*, *B. oleracea*, or *N. officinale*, which accumulated the ITC precursors A-GSL (**2**), 4MSOB-GSL (**1**), or 2PE-GSL (**3**), respectively, supported much lower *B. bassiana* growth than aphids fed on *B. napus*, which accumulated 2OH3But-GSL (**4**), a GSL that does not yield a stable ITC toxin after activation (Fig. 1B, D). Accordingly, radial growth rates of *B. bassiana* in vitro were negatively correlated with increasing concentrations of A-ITC (**2a**), 4MSOB-ITC (**1a**), and 2PE-ITC (**3a**), but not of the 2OH3But-GSL (**4**) product goitrin (**4b**) (Fig. 6A), suggesting differences in the toxicities of these GSL hydrolysis products. Similar differences have been previously attributed to the disparate metabolism of aliphatic, benzenic, and indolic GSL hydrolysis products, as for example by the insect *Phaedon cochleariae*, which conjugates different amino acids to hydrolysis products derived from different GSL structural types [46]. Our results may also help explain previous reports that the pathogenicity of *B. bassiana* towards its insect hosts is influenced by the plant on which the insect is feeding [47–50]. For instance, the susceptibility of the Brassicales specialist, *P. xylostella*, to *B. bassiana* has been found to vary depending on the plant species [51]. In this study, we demonstrated that differences in GSL composition altered the success of *B. bassiana* on cabbage aphids.

The fungus *B. bassiana* counteracts the toxicity of ITCs by metabolizing these GSL hydrolysis products. Using both non-targeted and targeted metabolomic analyses, we determined that this entomopathogen detoxifies dietary ITCs by conjugation to GSH, and can hence successfully infect even host insects that accumulate and activate GSLs. During the mercapturic acid pathway, ITCs are initially conjugated to GSH followed by the removal of Glu and Gly and *N*-acetylation leading to the formation of ITC-Cys-Glu, ITC-Cys-Gly, ITC-Cys, and ITC-NAC derivatives. These modifications profoundly alter the electrophilicity and polarity of ITCs, reducing their reactivity with other biological nucleophiles such as protein thiols and amines and enabling a rapid efflux from cells [52]. This pathway is characteristic of ITC metabolism in many generalist insect herbivores [53] and even mammals [54]. Various predators and parasitoids of insect herbivores also have mechanisms to circumvent GSL-derived toxins [7]. For example, a specialist parasitoid, *Diadegma semiclausum*, circumvents toxic 4MSOB-ITC (**1a**) in its caterpillar host *P. xylostella* by inducing GSL excretion [6]. In contrast, a generalist predatory lacewing, *C. carnea*, efficiently degrades ITCs ingested from caterpillar prey via conjugation to GSH in the mercapturic acid pathway followed by anal excretion [55].

Glutathione-*S*-transferases (GSTs) are major detoxification enzymes in both prokaryotes and eukaryotes that play a crucial role in the deactivation of several chemically reactive xenobiotics by conjugating the tripeptide GSH to electrophilic centers [56]. Three main GST subfamilies are generally recognized according to their distribution within the cell: cytosolic, microsomal (MAPEG), and mitochondrial (also known as the Kappa class) GSTs [57]. Moreover, GSTs have been further classified based on amino acid or nucleotide sequences, and immunological, kinetic, or structural properties, and it has been suggested that as many as forty-four distinct GST classes may exist [58]. However, most fungal GSTs do not fit easily into these major classes, and attempts at a uniform classification of fungal GSTs have not yet reached a consensus [59]. In this study, we classified seventeen detected BbGSTs based on sequence similarities into eleven previously described classes [59–62] namely Ure2p_like, GTT1, Sigma_like, EF1Bγ, N3, N2, N1, GHR, Zeta, etherase_LigE, Kappa, and one unclassified group, by searching for conserved domains within the amino acid sequence (Fig. 5A and Fig. S5). There is comparatively little knowledge of GST function in fungi in comparison with other organisms. In insects, the Epsilon and Delta class GSTs are often implicated in the detoxification of insecticides and plant defensive chemicals, including ITCs [37, 63–65]; however, such information is not available for fungal GSTs. This study represents the first investigation of GSTs related to plant-derived toxin detoxification in an entomopathogenic fungus. Curiously, a broad range of classes of BbGSTs, eight out of twelve, were induced by ITCs (Fig. 5B). Heavy metal treatment of a septate endophytic fungus (*Exophiala pisciphila*) also induced genes from diverse classes of GSTs [62].

Among the GSTs of *B. bassiana*, there were differences in substrate preference for ITCs. For instance, BbGSTs from the Ure2p, N3, and N2 classes have higher activity toward the aliphatic methylsulfinyl-containing 4MSOB-ITC (**1a**) than other ITCs (Fig. 5C); BbGSTU, which is phylogenetically distant from BbGSTUre2p and BbGSTN21 (Fig. S5), has an apparent preference towards A-ITC (**2a**), an aliphatic ITC with an unsaturated C-C bond (Fig. 5C); BbGSTe1, which is relatively phylogenetically isolated, had significantly higher activity with 2PE-ITC (**3a**), a benzenic ITC, than 4MSOB-ITC (**1a**) and A-ITC (**2a**) (Fig. 5C). In previous studies of GST activities towards various plant ITCs, enzyme preferences appeared to correlate to different side-chain structures [37]. Other GSL detoxification enzymes, such as the GSL sulfatases (GSSs) from *P. xylostella* and the whitefly *Bemisia tabaci*, are also specialized for GSLs with different side-chains [66, 67]. Here, we observed that BbGSTs of different classes were sometimes active on the same ITC substrate, suggesting that GSTs of different types might cooperate to help *B. bassiana* cope with these dietary toxins, although more ITCs should be tested to substantiate this pattern. Given the large variety of GSL structures that may be sequestered by cabbage aphids from crucifer plants [30], and the large variety of plant toxins present in other potential hosts of *B. bassiana*, a flexible detoxification system might be advantageous to this non-specialized entomopathogenic fungus.

The detoxification of plant defense compounds by insect herbivores is often associated with energetic or metabolic costs that lead to reductions in herbivore performance. For example, growth and development of the crucifer-specialist herbivore *P. xylostella* were negatively correlated with the expression of its sulfatase gene and enzyme, which are responsible for GSL detoxification [55]. Thus, in the present study, the inducibility of *BbGST* genes might serve to minimize the physiological costs of detoxification in *B. bassiana* (Fig. 5B). For instance, *BbGSTU* expression was only up-regulated (Fig. S6) at high 4MSOB-ITC (**1a**) concentrations (400 µM) that strongly reduce fungal growth (Fig. 6A). Detoxification via GSTs is also costly in terms of reductions in the supply of GSH, a tripeptide that participates in detoxification, maintenance of redox homeostasis and many other cellular functions. Here, depletion of GSH after ITC treatment was demonstrated by the manner in which GSH complementation restored *B. bassiana* growth (Fig. 6C). A similar situation was observed during ITC detoxification in *Spodoptera littoralis*, where the diversion of GSH to 4MSOB-ITC (**1a**) detoxification delayed larval development [34, 68]. In spite of these costs, the crucial role of GSTs in allowing *B. bassiana* to infect the cabbage aphid is supported by several lines of evidence. First, the only ITC metabolites detected in *B. bassiana* were intermediates and products of the mercapturic acid pathway (Fig. 3) suggesting that this is the major route of GSL processing in this species, a route for which GSTs catalyze the first step. Second, several GST-encoding genes were induced by exposure of *B. bassiana* to specific ITCs (Fig. 5B), and nearly all the induced genes encoded a protein that had high activity with at least one ITC (Fig. 5C). Third, the ability of added GSH, a co-substrate for all GSTs, to increase *B. bassiana* growth in the presence of ITCs (Fig. 6C) is consistent with the importance of GSTs in facilitating fungal growth in GSL-defended cabbage aphids.

In conclusion, our results demonstrate how plant GSL defense compounds sequestered by a specialized aphid herbivore can negatively affect *B. bassiana*, an entomopathogenic fungus frequently used in pest control. The fungus possesses a general detoxification mechanism for the major GSL hydrolysis products involving GST enzymes that may facilitate aphid infection. However, these enzymes are likely not effective at high ITC concentrations due to limitations in the supply of the GSH cosubstrate.

## Methods

Detailed materials and methods with references are described in the supplementary methods (Supplementary file 3).

## Supplementary information

Supplementary file 1

Supplementary file 2

Supplementary file 3

Supplementary file 4

Table S1

Table S2

Table S3

Table S4

Table S5

Table S6

Table S7

Table S8

Table S9

Table S10

Table S11

## Data Availability

All the data needed to understand and assess the conclusions of this research are available in the manuscript and supplementary files; DNA sequences of BbGSTs have been submitted to the NCBI database, and the accession number for BbGSTs (from OP856926 to OP856942) and homologs analyzed in this manuscript are listed in the supplementary materials (detailed in Tables S6 and S7).
