# Supplementary material for: Metabolism of plant-derived toxins from its insect host increases the success of the entomopathogenic fungus Beauveria bassiana
Source: ISME J. 2023 Jul 21;17(10):1693–704. doi: 10.1038/s41396-023-01480-3 (PMC10504261; doi:10.1038/s41396-023-01480-3)
Supplement: Supplementary file 1 [file 41396_2023_1480_MOESM1_ESM.docx]

**Metabolism of plant-derived toxins from its insect host increases the success of the entomopathogenic fungus *Beauveria bassiana*, by Sun, R. *et al*.**

**Supplementary Figures**

**
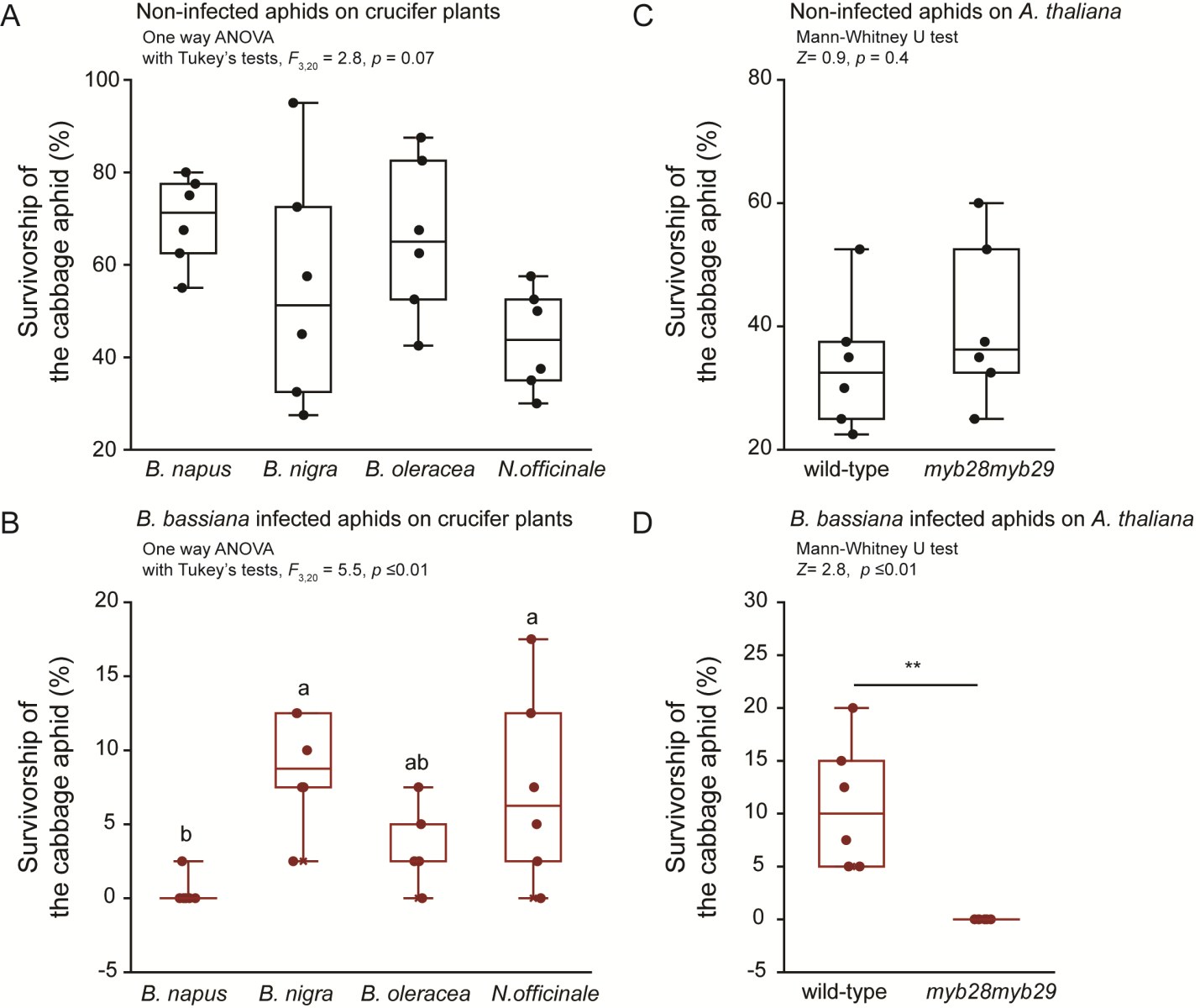
**

**Fig. S1 Adult survivorship and offspring production of the cabbage aphid is affected by their Brassicales host plant.** The survivorship of uninfected (**A**) and *B. bassiana*-infected (**B**) cabbage aphids fed on *Brassica napus*, *B. nigra*, *B. oleracea*, and *Nasturtium officinale* as host plants. The survivorship of uninfected (**C**) and *B. bassiana*-infected (**D**) cabbage aphids fed on *Arabidopsis thaliana* wild-type Col-0 plants and *myb28myb29* mutant (KO) plants. The statistical methods are listed in the figures. Asterisks and lowercase letters denote statistically significant differences.


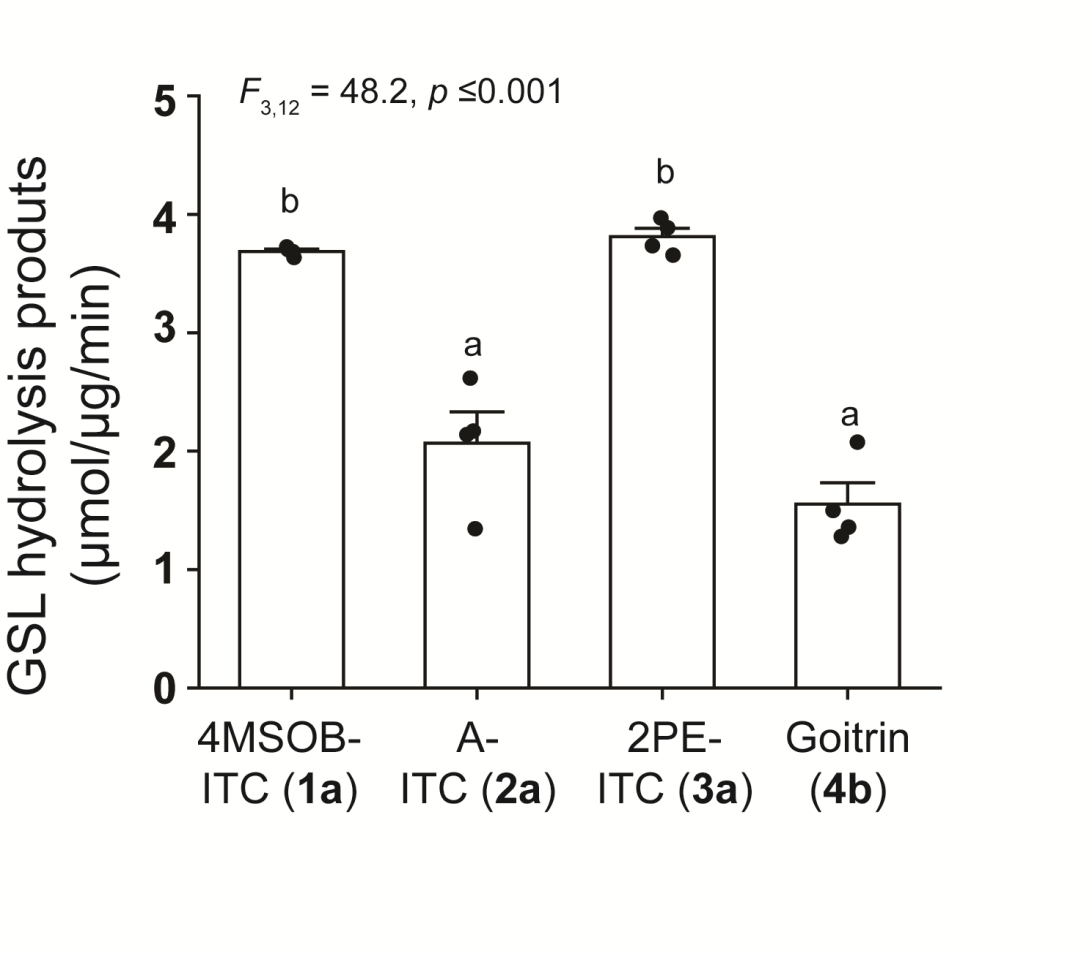


**Fig. S2 GSL hydrolysis products formed by cabbage aphids.** 4MSOB-ITC (**1a**), A-ITC (**2a**), 2PE-ITC (**3a**), and goitrin (**4b**) (µmol substrates · µg protein^-1^ · min^-1^) were formed by the reaction of aphid crude protein extract with 4MSOB-GSL (**1**), A-GSL (**2**), 2PE-GSL (**3**), and 2OH3But-GSL (**4**), respectively (n=4). Statistically significant differences between means (±SE) were determined by Tukey HSD tests in conjunction with one-way ANOVA and are denoted by lowercase letters.


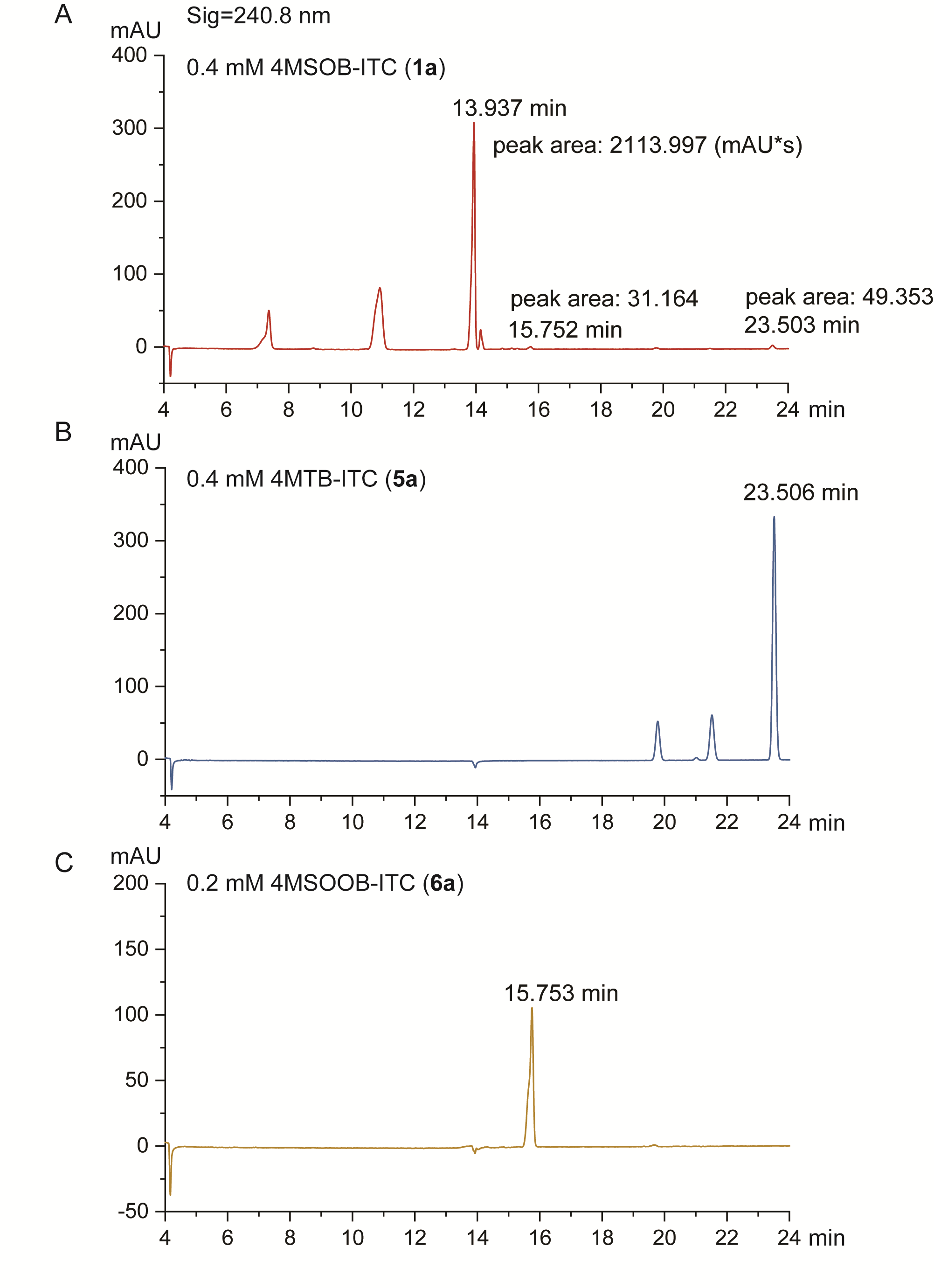


**Fig. S3 4MTB-ITC (5a) and 4MSOOB-ITC (6a) are present as impurities in the 4MSOB-ITC (1a) standard used in this study.** 4MTB-ITC (**5a**) and 4MSOOB-ITC (**6a**) are present at levels of 1.47% and 2.33%, respectively, in our batch of 4MSOB-ITC (**1a**) as determined by HPLC-UV. Chromatograms of the 4MSOB-ITC (**1a**) used (**A**), and 4MTB-ITC (**4a**) (**B**) and 4MSOOB-ITC (**6a**) (**C**) standards were run with the UV detection set at a wavelength of 240.8 nm.


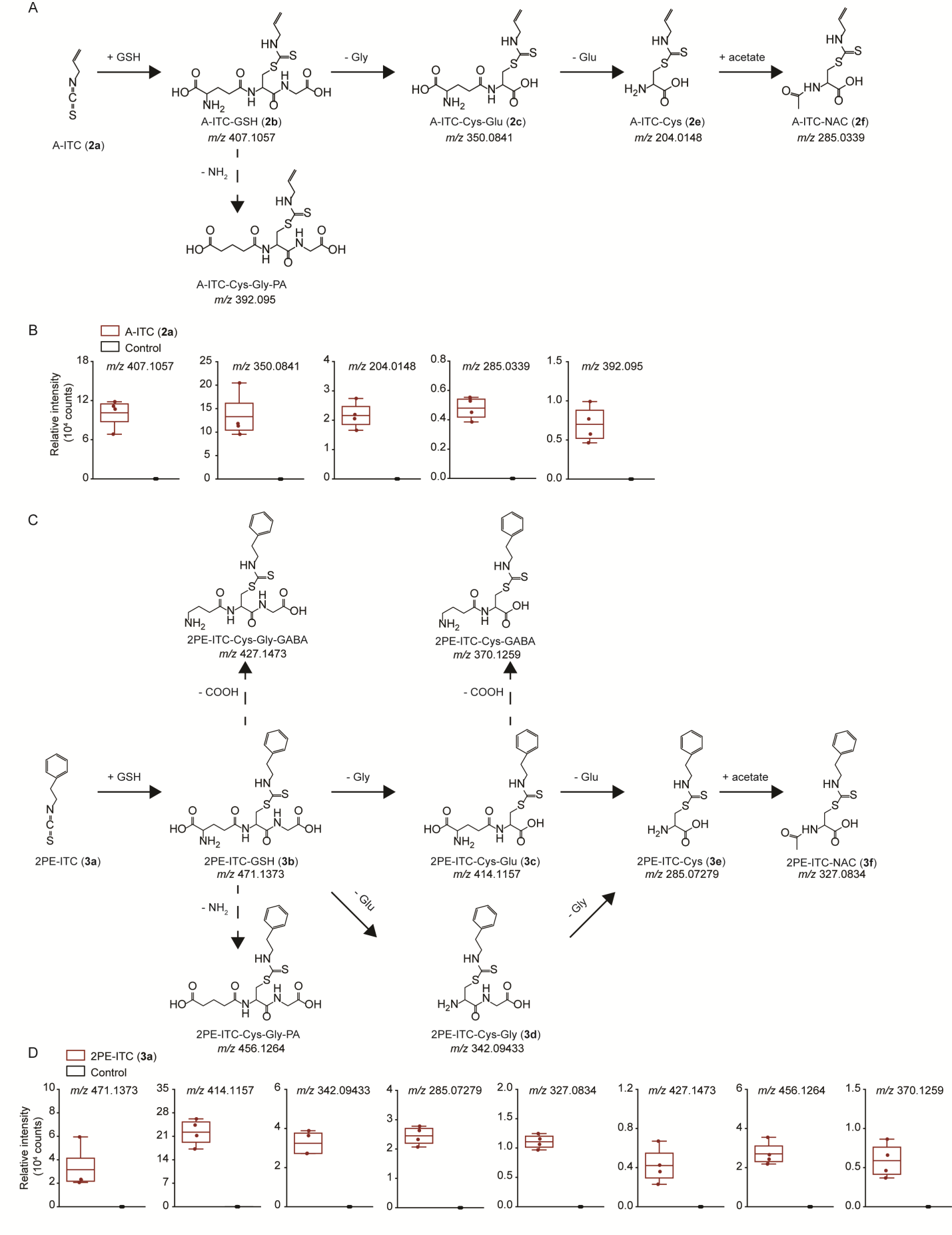


**Fig. S4 A-ITC (2a) and 2PE-ITC (3a) are metabolized by the mercapturic acid pathway in *B. bassiana*.** Schema for the conversion of A-ITC (**2a**) (**A**) and 2PE-ITC (**3a**) (**C**) to GSH conjugates and derivatives. Relative intensities of A-ITC (**B**) and 2PE-ITC (**D**) conjugates measured via non-targeted UHPLC-qTOFMS analyses. The chromatography and mass spectra of the putative products A-ITC-Cys-Gly-PA (pentanedioic acid), 2PE-ITC-Cys-Gly-GABA (α-aminobutyric acid), 2PE-ITC-Cys-GABA, and 2PE-ITC-Cys-Gly-PA are shown in the Supplementary file 4.


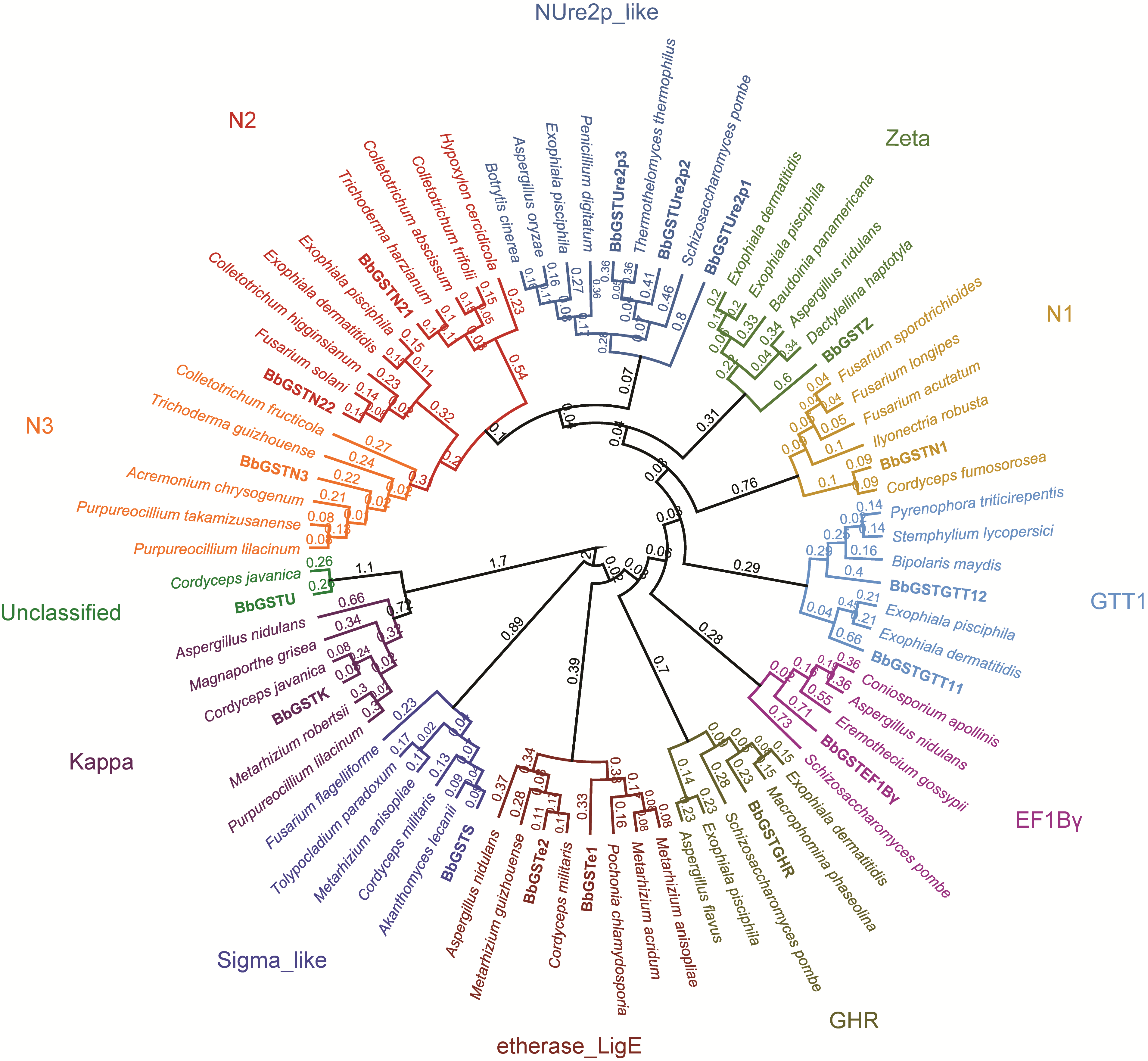


**Fig. S5 Phylogenetic relationships among the fungal GSTs.** Phylogenetic analysis of seventeen *B. bassiana* GSTs along with sixty other fungal GSTs. Accession numbers of these genes are listed in Table S6 and Table S7. The amino acid sequences were aligned and a UPGMA tree was generated. The branch labels represent the expected amino acid substitutions per site. The twelve clades harboring the BbGSTs are differently colored.


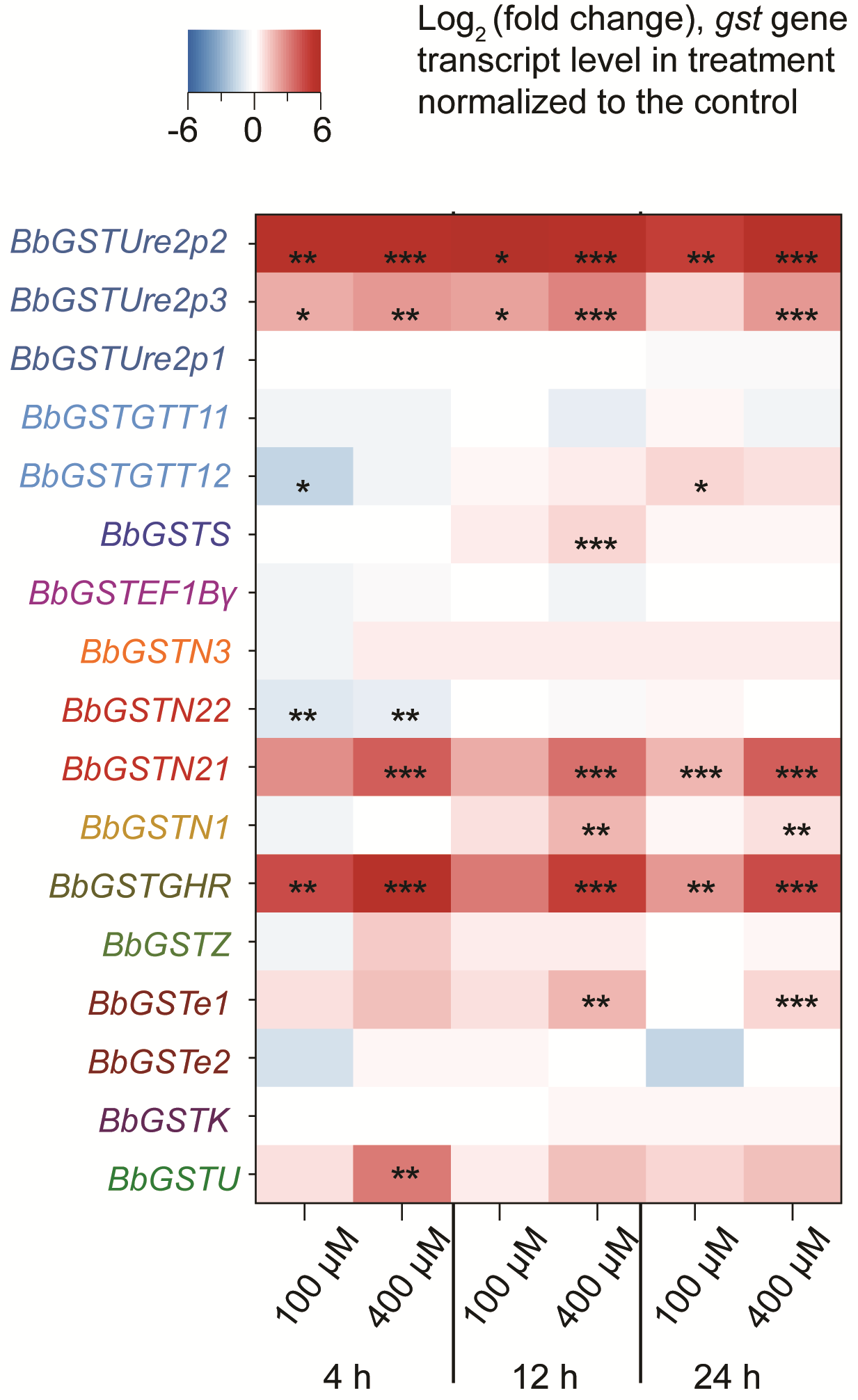


**Fig. S6 Inducibility of *B. bassiana* GST-encoding genes by 4MSOB-ITC (1a).** Heatmap showing the expression (relative to *Bbactin*) of 17 GST-encoding genes in *B. bassiana* (*n*= 5) after incubation with 100 µM or 400 µM 4MSOB-ITC (**1a**) in PDB medium for 4 h, 12 h, and 24 h*.* The data show log_2_ fold-changes of *GST* gene expression level in *B. bassiana* incubated with 4MSOB-ITC (**1a**) normalized to expression in *B. bassiana* incubated with solvent (control). Statistically significant differences between means (±SE) were determined by Tukey HSD tests in conjunction with one-way ANOVA and are denoted by asterisks.


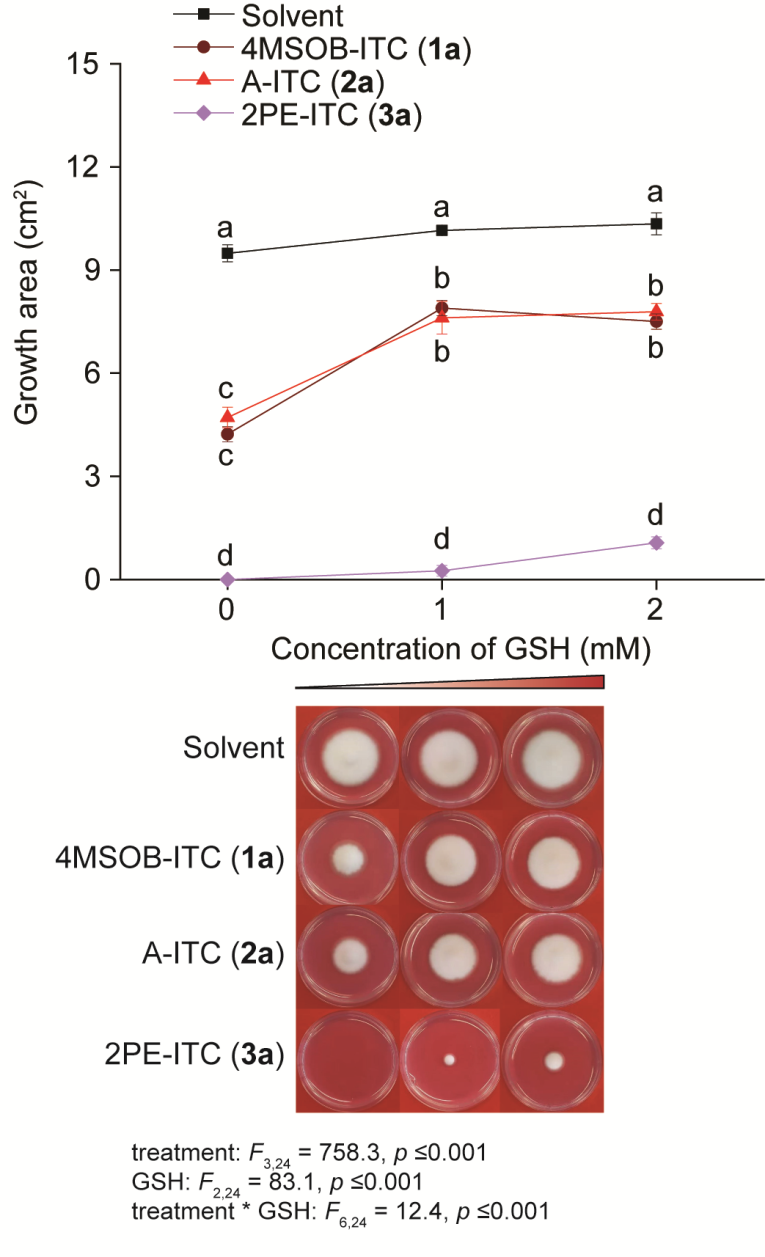


**Fig. S7 GSH supplementation alleviates the toxicity of ITCs towards *B. bassiana*.** Depicted is the growth of *B. bassiana* on basic medium agar plates containing 60 µM 4MSOB-ITC (**1a**), A-ITC (**2a**), or 2PE-ITC (**3a**), and increasing concentrations of GSH (*n*=3). Statistically significant differences between means (±SE) for each group were determined by Tukey HSD tests in conjunction with two-way ANOVA and are denoted by lowercase letters.


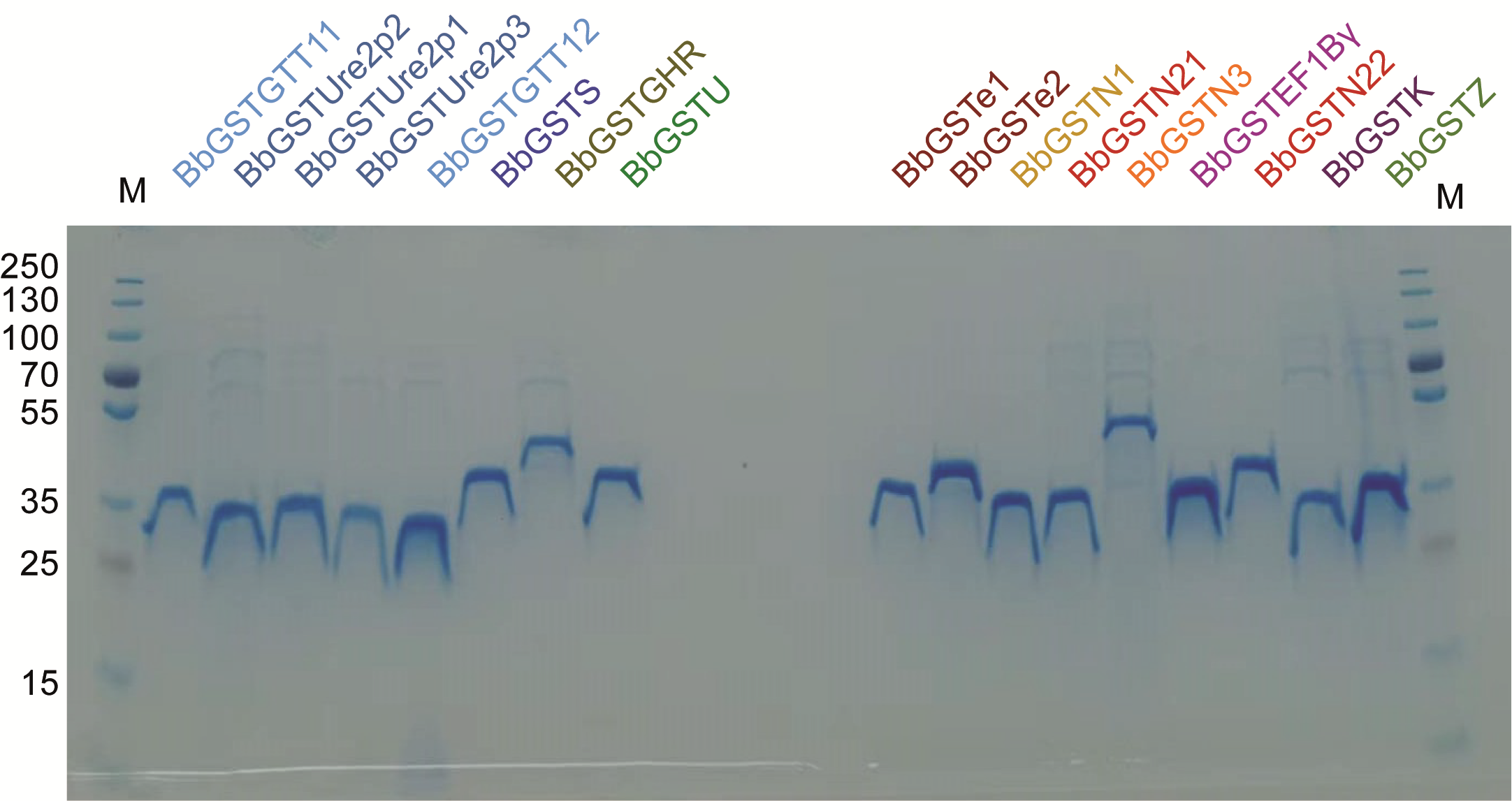


**Fig. S8 SDS-PAGE analysis of purified *B. bassiana* GST proteins expressed in the *Escherichia coli* BL21 (DE3) strain.** *Bbgst* sequences were independently inserted into the pET28a vector and transformed into *E. coli* to heterologously express recombinant tagged proteins. Proteins were purified via His-tag affinity. The molecular weights of the proteins are listed in Table S6.
