# Supplementary material for: Metabolism of plant-derived toxins from its insect host increases the success of the entomopathogenic fungus Beauveria bassiana
Source: ISME J. 2023 Jul 21;17(10):1693–704. doi: 10.1038/s41396-023-01480-3 (PMC10504261; doi:10.1038/s41396-023-01480-3)

**Supplementary file 2: NMR analyses of ITC conjugates**

**Characterization of compounds**

**2PE-ITC-Cys: ^1^H-NMR** (400 MHz, CD_3_OD) δ 7.37–7.14 (m, 5H), 4.06 (dd, *J* = 14.8, 4.2 Hz, 1H), 3.95 – 3.73 (m, 3H), 3.64 (dd, *J* = 14.8, 7.0 Hz, 1H), 2.96 (t, *J* = 7.4 Hz, 2H);

**^13^C-NMR** (101 MHz, CD_3_OD) δ 197.5, 172.3, 140.2, 129.8, 129.6, 129.5, 127.5, 56.0, 36.4, 34.9.

**2PE-ITC-Cys-Glu: ^1^H-NMR** (400 MHz, D_2_O) δ 7.39 (dd, *J* = 9.1, 5.5 Hz, 2H), 7.35 – 7.28 (m, 3H), 4.49 (dd, *J* = 7.5, 4.5 Hz, 1H), 3.99 (dd, *J* = 7.5, 6.4 Hz, 2H), 3.85 (dd, *J* = 14.4, 4.5 Hz, 1H), 3.76 (dd, *J* = 6.9, 5.7 Hz, 1H), 3.49 (dd, *J* = 14.4, 7.5 Hz, 1H), 3.01 (t, *J* = 6.9 Hz, 2H), 2.45 (t, *J* = 7.7 Hz, 2H), 2.22 – 2.05 (m, 2H);

**^13^C-NMR** (101 MHz, D_2_O) δ 197.4, 176.2, 174.2, 174.0, 138.9, 128.9, 128.7, 126.6, 54.4, 54.3, 48.2, 36.3, 33.2, 31.9, 26.4.

**2PE-ITC-Cys-Gly**: **^1^H-NMR** (400 MHz, D_2_O) δ 7.44 – 7.25 (m, 5H), 3.99 (t, *J* = 6.9 Hz, 2H), 3.92 (t, *J* = 6.2 Hz, 1H), 3.83 (dd, *J* = 19.9, 9.2 Hz, 1H), 3.75 – 3.65 (m, 2H), 3.56 (dd, *J* = 14.6, 6.8 Hz, 1H), 3.01 (t, *J* = 7.0 Hz, 2H);

**^13^C-NMR** (101 MHz, D_2_O) δ 196.4, 176.2, 176.1, 138.9, 128.9, 128.7, 126.6, 53.8, 53.0, 48.2, 43.3, 37.0, 33.2.

**2PE-ITC-GSH**: **^1^H-NMR** (400 MHz, D_2_O) δ 7.42 – 7.36 (m, 2H), 7.32 (d, *J* = 7.0 Hz, 3H), 3.99 (t, *J* = 6.8 Hz, 2H), 3.87 (dd, *J* = 14.6, 4.8 Hz, 1H), 3.81 – 3.69 (m, 4H), 3.52 (dd, *J* = 14.6, 8.3 Hz, 1H), 3.01 (t, *J* = 6.9 Hz, 2H), 2.49 (t, *J* = 7.7 Hz, 2H), 2.14 (dd, *J* = 14.6, 7.2 Hz, 2H);

**^13^C-NMR** (101 MHz, D_2_O) δ 196.8, 176.2, 174.9, 174.0, 171.3, 138.9, 128.9, 128.7, 126.6, 54.2, 53.0, 48.2, 43.4, 35.4, 33.2, 31.6, 26.3.

**2PE-ITC-NAC**: **^1^H-NMR** (400 MHz, CD_3_OD) δ 7.28 (t, *J* = 7.3 Hz, 2H), 7.25 – 7.17 (m, 3H), 4.67 (dd, *J* = 8.0, 4.5 Hz, 1H), 3.96 (dd, *J* = 14.1, 4.7 Hz, 1H), 3.86 (td, *J* = 7.2, 3.2 Hz, 2H), 3.52 (dd, *J* = 14.1, 8.6 Hz, 1H), 2.95 (t, *J* = 7.4 Hz, 2H), 1.96 (s, 3H);

**^13^C-NMR** (101 MHz, CD_3_OD) δ 198.0, 173.7 173.3, 140.2, 129.8, 129.5, 127.4, 53.9, 49.6, 36.8, 34.9, 22.5.

**Allyl-ITC-Cys**: **^1^H-NMR** (400 MHz, D_2_O) δ 6.00 – 5.86 (m, 1H), 5.31 – 5.17 (m, 2H), 4.35 (s, 2H), 4.22 – 4.04 (m, 2H), 3.72 (dd, *J* = 14.7, 6.3 Hz, 1H);

**^13^C-NMR** (101 MHz, D_2_O) δ 196.5, 172.3, 131.6, 117.3, 54.3, 49.4, 34.8.

**Allyl-ITC-Cys-Glu**: **^1^H-NMR** (400 MHz, D_2_O) δ 5.92 (ddd, *J* = 22.4, 10.5, 5.3 Hz, 1H), 5.32 – 5.18 (m, 2H), 4.69 (dd, *J* = 7.8, 4.5 Hz, 1H), 4.34 (d, *J* = 5.3 Hz, 2H), 3.97 (dd, *J* = 14.5, 4.5 Hz, 1H), 3.82 (t, *J* = 6.3 Hz, 1H), 3.60 (dd, *J* = 14.5, 7.8 Hz, 1H), 2.49 (t, *J* = 7.8 Hz, 2H), 2.27 – 2.06 (m, 2H);

**^13^C-NMR** (101 MHz, D_2_O) δ 197.6, 174.9, 174.4, 173.6, 131.7, 116.9, 54.0, 53.4, 49.1, 35.9, 31.6, 26.2.

**Allyl-ITC-GSH**: **^1^H-NMR** (400 MHz, D_2_O) δ 5.92 (ddd, *J* = 16.0, 10.4, 5.1 Hz, 1H), 5.33 – 5.19 (m, 2H), 4.34 (d, *J* = 5.2 Hz, 2H), 3.99 (d, *J* = 4.1 Hz, 2H), 3.93 (dd, *J* = 14.6, 5.0 Hz, 1H), 3.82 (t, *J* = 6.4 Hz, 1H), 3.60 (dd, *J* = 14.7, 8.3 Hz, 1H), 2.52 (td, *J* = 7.5, 2.9 Hz, 2H), 2.17 (dd, *J* = 14.2, 7.6 Hz, 2H);

**^13^C-NMR** (101 MHz, D_2_O) δ 197.2, 174.8, 173.6, 173.5, 172.1, 131.7, 117.0, 53.8, 53.0, 49.1, 41.6, 35.4, 31.4, 26.0.

**Allyl-ITC-NAC**: **^1^H-NMR** (400 MHz, CD_3_OD) δ 5.98 – 5.83 (m, 1H), 5.22 (d, *J* = 18.7 Hz, 1H), 5.14 (d, *J* = 10.3 Hz, 1H), 4.51 (dd, *J* = 7.1, 4.4 Hz, 1H), 4.30 (s, 2H), 3.86 (dd, *J* = 13.9, 4.3 Hz, 1H), 3.60 (dd, *J* = 13.8, 7.2 Hz, 1H), 1.94 (s, 3H);

**^13^C-NMR** (101 MHz, CD_3_OD) δ 199.4, 176.7, 172.7, 133.9, 117.5, 56.0, 50.2, 38.6, 22.8.

**4MSOB-ITC-Cys-Glu**: **^1^H-NMR** (400 MHz, D_2_O) δ 3.99-3.91 (m, 1H), 3.89-3.81 (m, 1H), 3.79-3.73 (m, 2H), 3.62-2.54 (m, 1H), 2.98-2.87 (m, 2H), 2.70 (s, 3H), 2.52-2.44 (m, 2H), 2.21-2.09 (m, 2H), 1.89-1.74 (m, 4H);

**^13^C-NMR** (101 MHz, D_2_O) δ 197.1, 52.1, 46.5, 45.8, 36.4, 35.7, 31.5, 26.6, 26.2, 26.2, 19.4.

**4MSOB-ITC-Cys-Gly**: **^1^H-NMR** (400 MHz, D_2_O) δ 4.41 (t, *J* = 6.3 Hz, 1H), 4.05 – 3.72 (m, 6H), 2.94 (dd, *J* = 14.3, 6.5 Hz, 2H), 2.71 (s, 3H), 1.94 – 1.74 (m, 4H);

**^13^C-NMR** (101 MHz, D_2_O) δ 195.3, 174.3, 167.9, 52.8, 52.1, 46.8, 42.4, 36.4, 34.4, 26.2, 19.4.

**4MSOOB-ITC-Cys**: **^1^H-NMR** (400 MHz, D_2_O) δ 4.14 (dd, *J* = 10.6, 4.1 Hz, 2H), 3.84 – 3.66 (m, 3H), 3.36-3.32 (m, 2H), 3.14 – 3.07 (m, 3H), 2.02 – 1.80 (m, 4H);

**^13^C-NMR** (101 MHz, D_2_O) δ 196.6, 179.7, 54.4, 53.2, 46.5, 39.6, 34.8, 25.9, 19.0.

**4MSOOB-ITC-GSH**: **^1^H-NMR** (400 MHz, D_2_O) δ 3.93 (dd, *J* = 14.7, 4.9 Hz, 1H), 3.84 – 3.70 (m, 6H), 3.57 (dd, *J* = 14.6, 8.2 Hz, 1H), 3.34 (t, *J* = 10.3 Hz, 2H), 3.10 (s, 3H), 2.56 – 2.47 (m, 2H), 2.20 – 2.10 (m, 2H), 1.89-1.85 (m, 4H);

**^13^C-NMR** (101 MHz, D_2_O) δ 197.1, 177.8, 176.2, 174.9, 171.3, 54.2, 53.2, 53.0, 46.3, 43.4, 39.6, 35.5, 31.6, 26.4, 26.0, 19.0.

**4MTB-ITC-Cys-Glu**: **^1^H-NMR** (400 MHz, D_2_O) δ 4.55 (dd, *J* = 7.7, 4.4 Hz, 1H), 3.92 (dd, *J* = 14.3, 4.4 Hz, 1H), 3.74 (t, *J* = 6.9 Hz, 2H), 3.69 – 3.64 (m, 1H), 3.54 (dd, *J* = 14.4, 7.7 Hz, 1H), 2.59 (t, *J* = 7.2 Hz, 2H), 2.45 (t, *J* = 7.7 Hz, 2H), 2.15 – 2.08 (m, 5H), 1.77 (dd, *J* = 14.5, 7.5 Hz, 2H), 1.68 (dd, *J* = 14.7, 7.7 Hz, 2H);

**^13^C-NMR** (101 MHz, D_2_O) δ 197.1, 176.9, 176.3, 174.5, 54.6, 54.5, 54.4, 46.9, 36.5, 32.8, 32.0, 26.3, 25.6, 14.0.

**4MTB-ITC-GSH**: **^1^H-NMR** (400 MHz, D_2_O) δ 3.93 (dd, *J* = 14.6, 4.8 Hz, 1H), 3.79-3.77 (m, 3H), 3.68 (t, *J* = 6.4 Hz, 1H), 3.64 – 3.59 (m, 1H), 3.32 (dd, *J* = 14.3, 4.4 Hz, 1H), 2.98 (dd, *J* = 14.2, 9.5 Hz, 1H), 2.59 (t, *J* = 7.2 Hz, 2H), 2.47 (t, *J* = 9.0 Hz, 2H), 2.11 (s, 3H), 2.10 – 2.03 (m, 2H), 1.76 (dd, *J* = 14.9, 6.9 Hz, 2H), 1.67 (dd, *J* = 14.7, 7.5 Hz, 2H);

**^13^C-NMR** (101 MHz, D_2_O) δ 196.6, 176.2, 175.3, 171.8, 171.4, 54.6, 54.4, 52.5, 46.9, 43.4, 35.5, 32.7, 31.6, 26.3, 25.6, 14.0.

**NMR spectra**


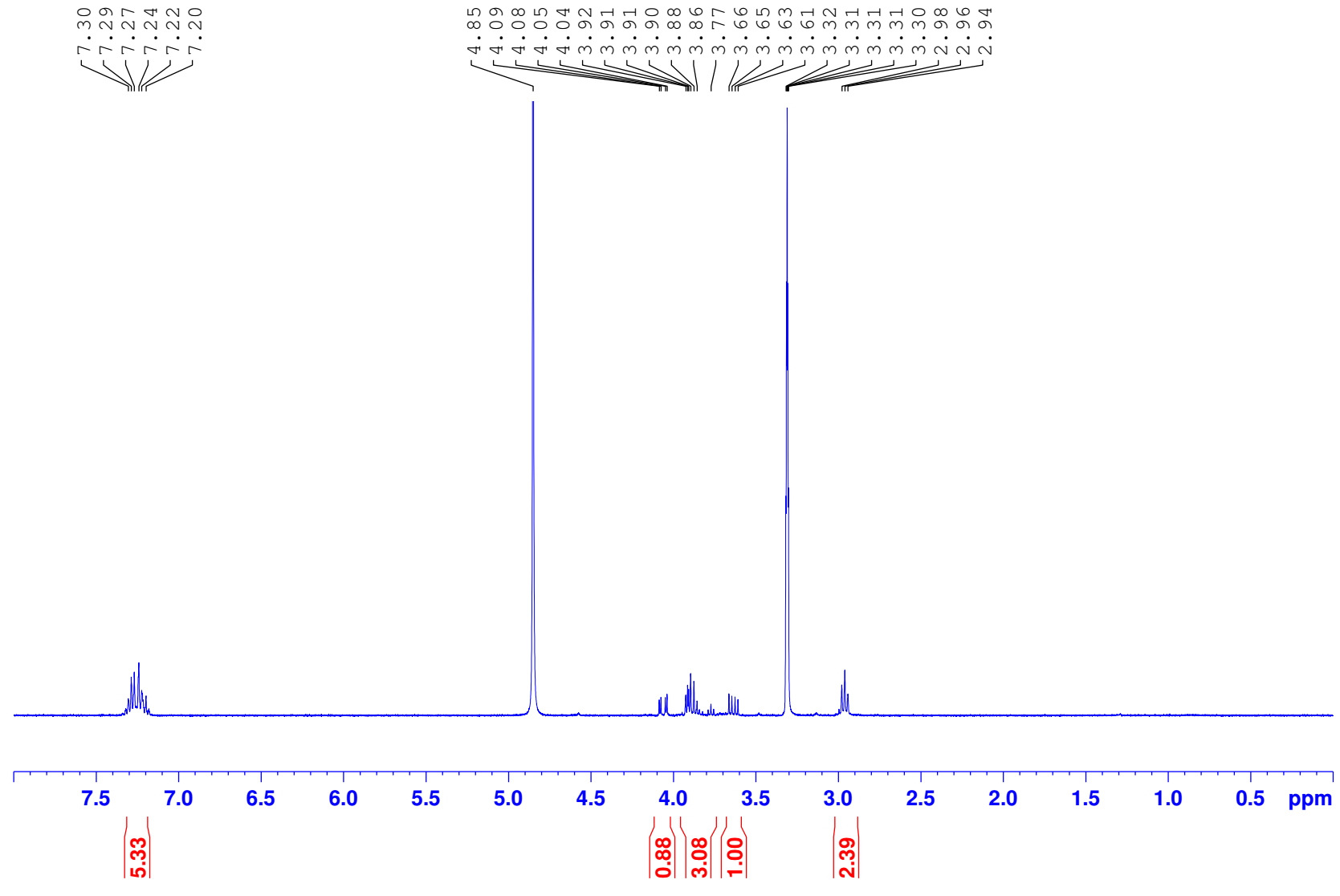


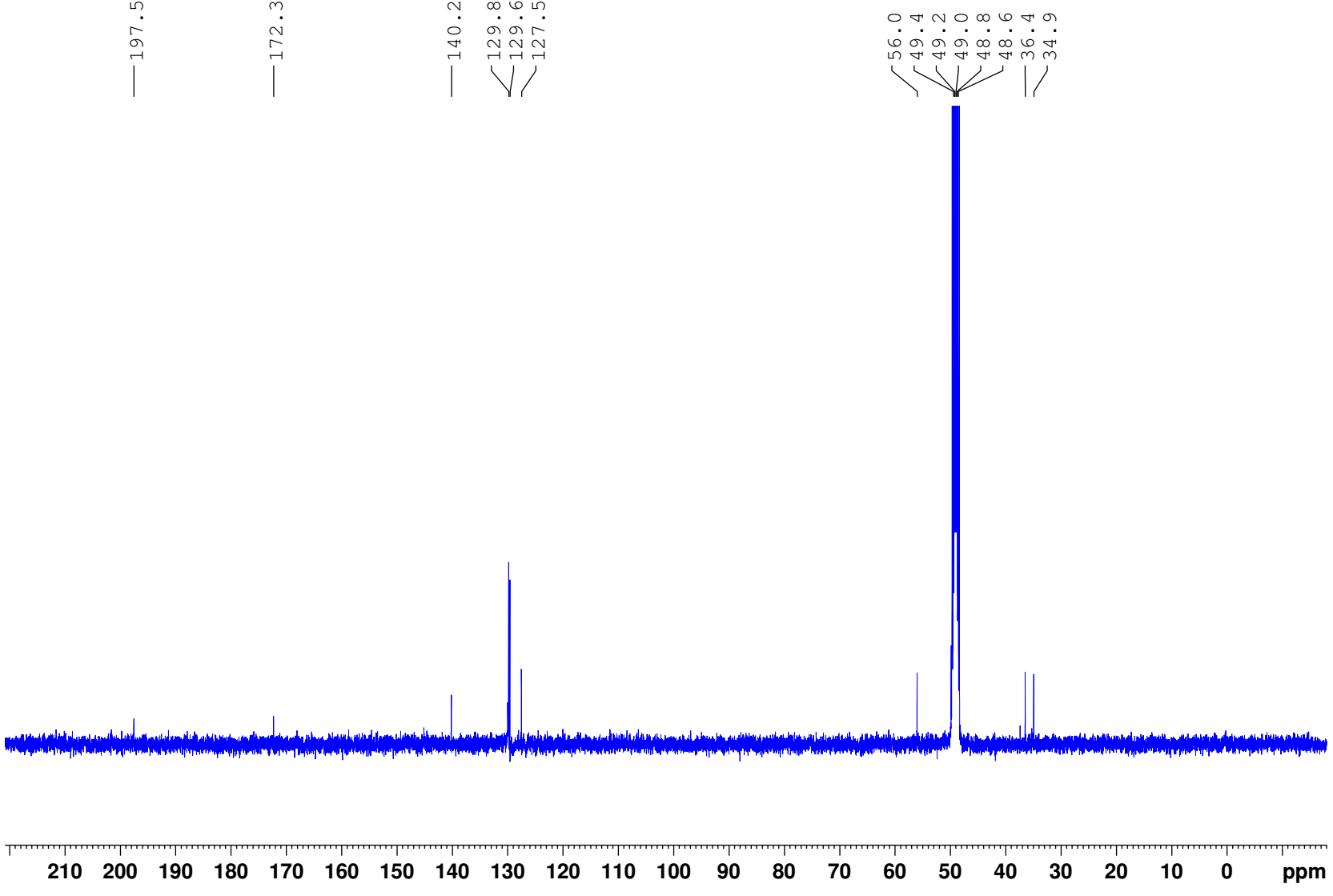


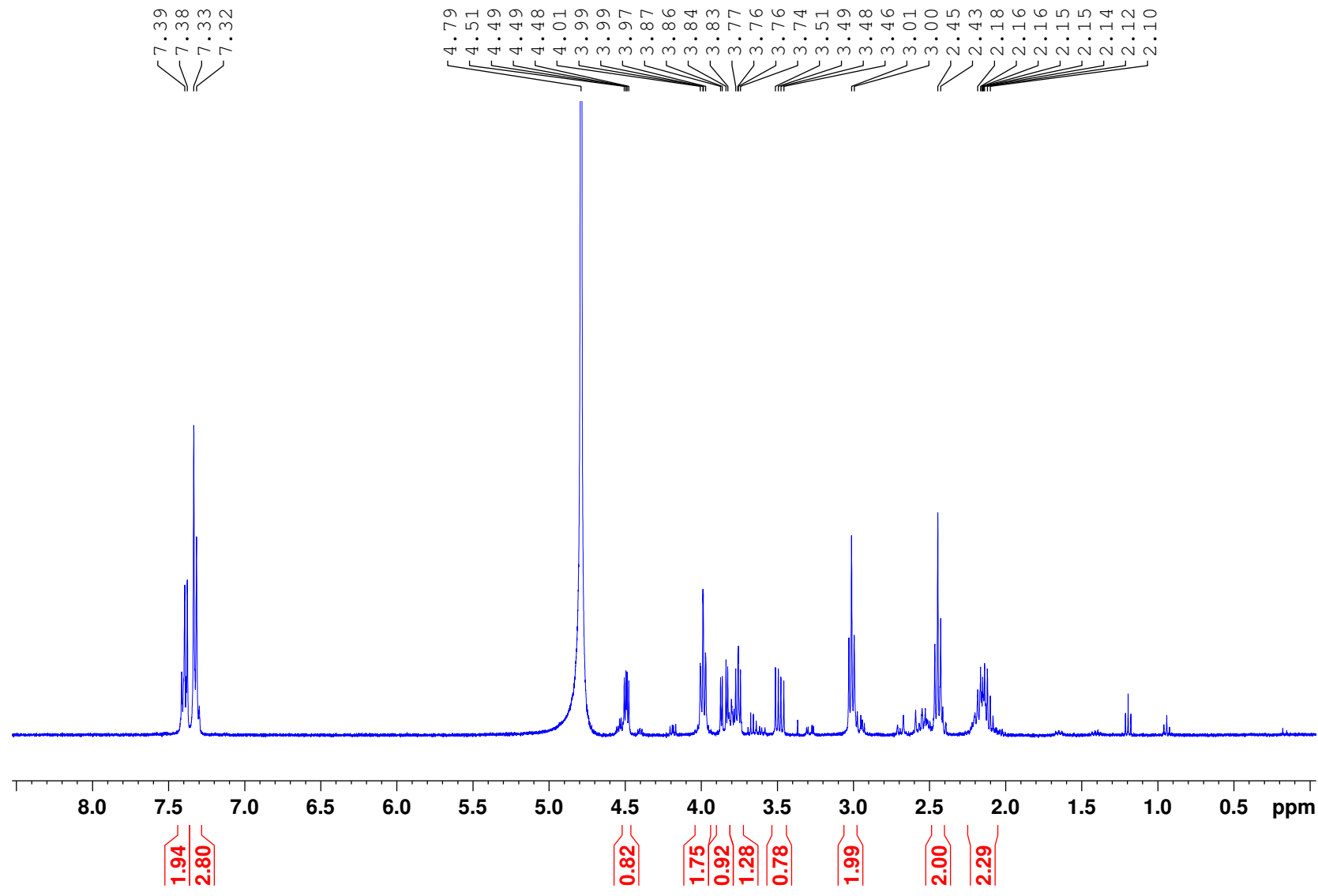


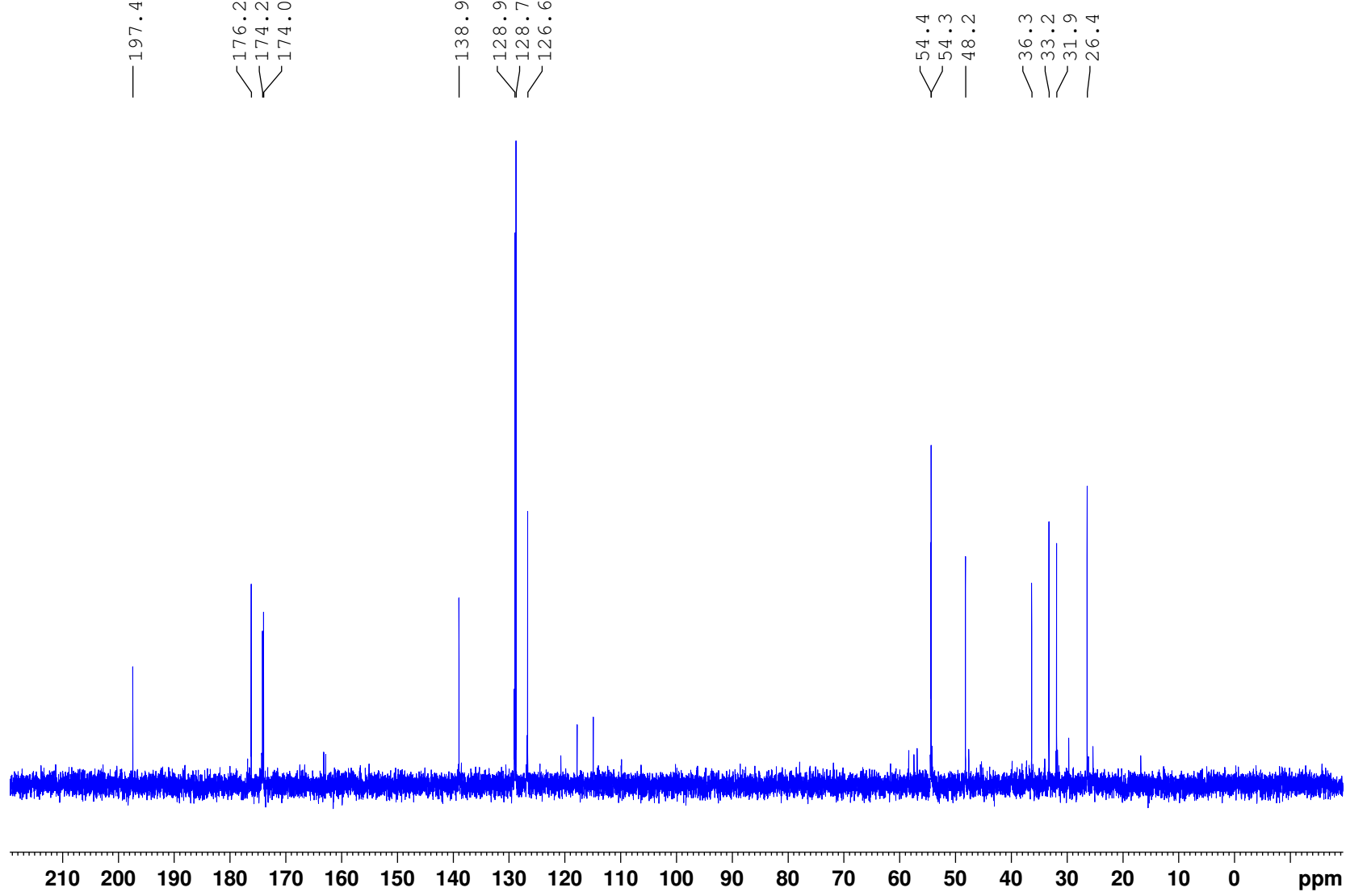


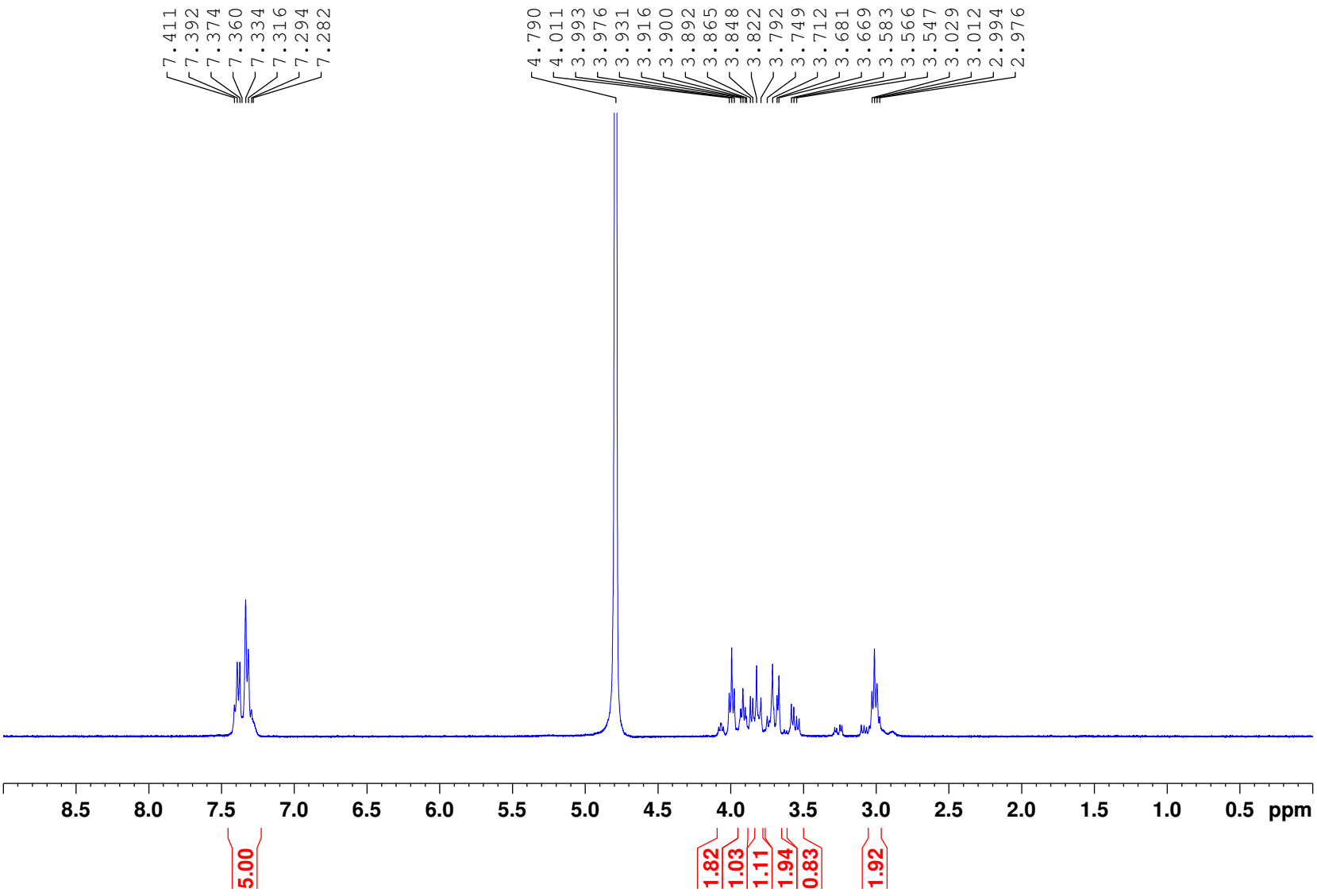


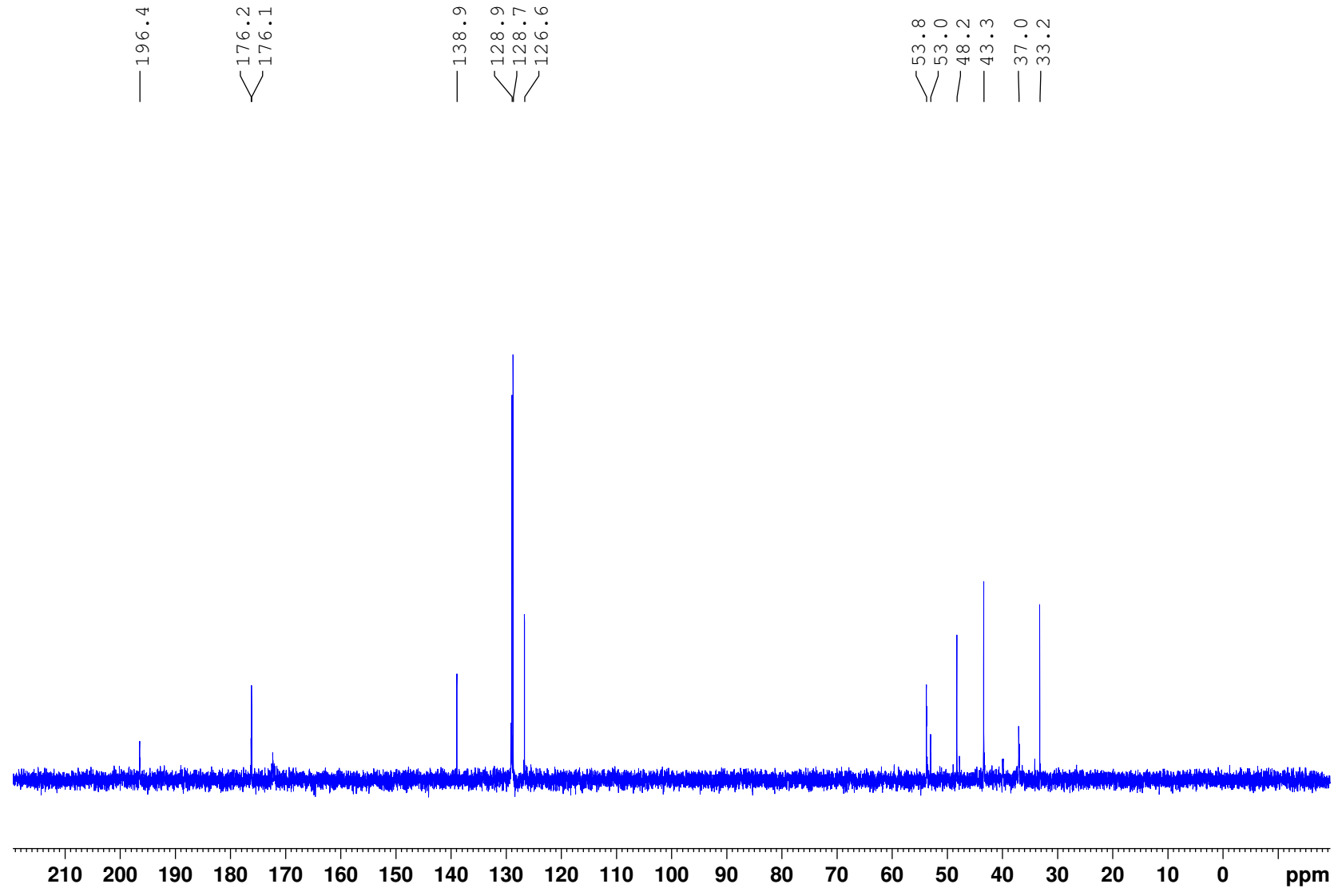


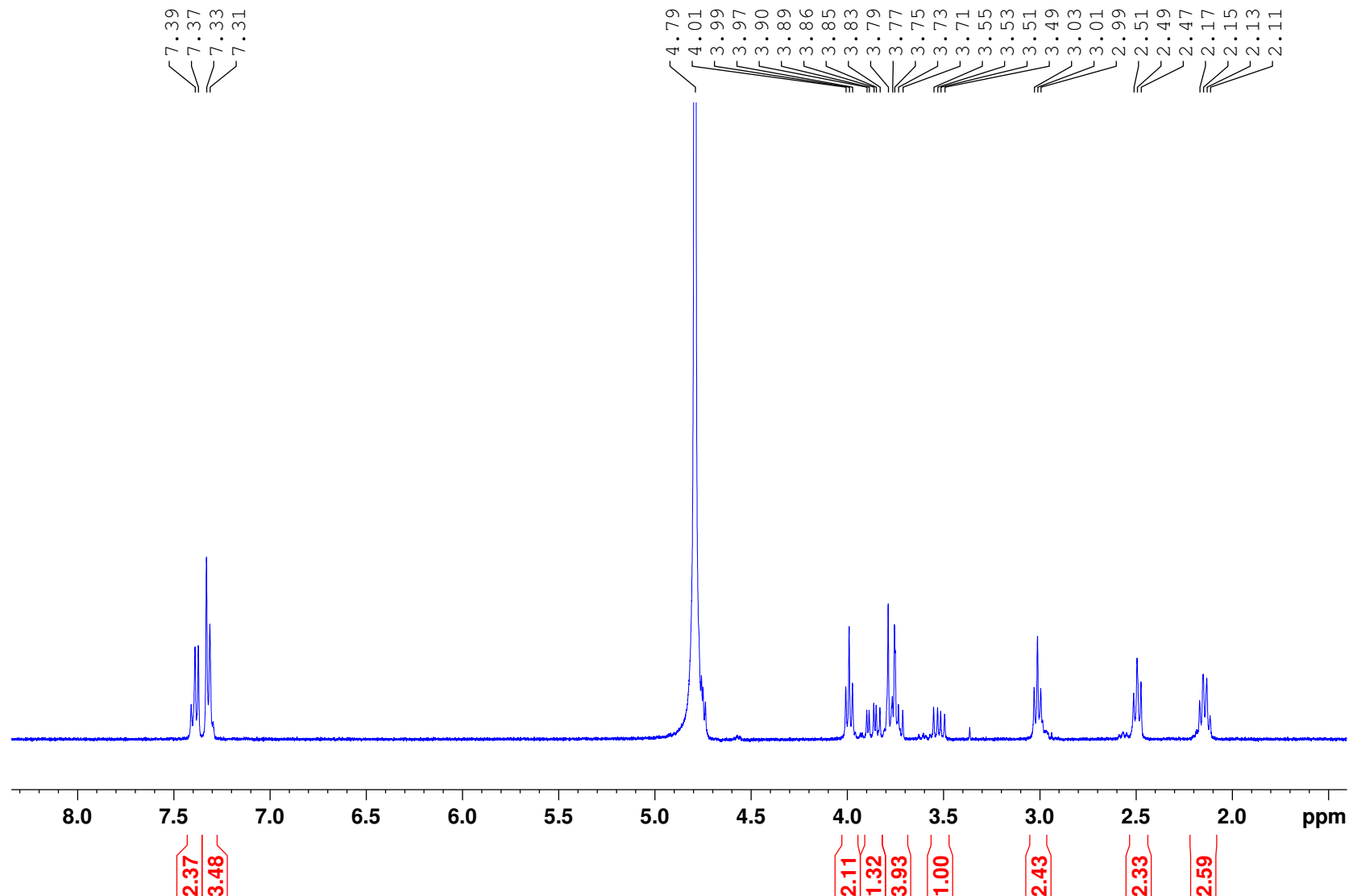


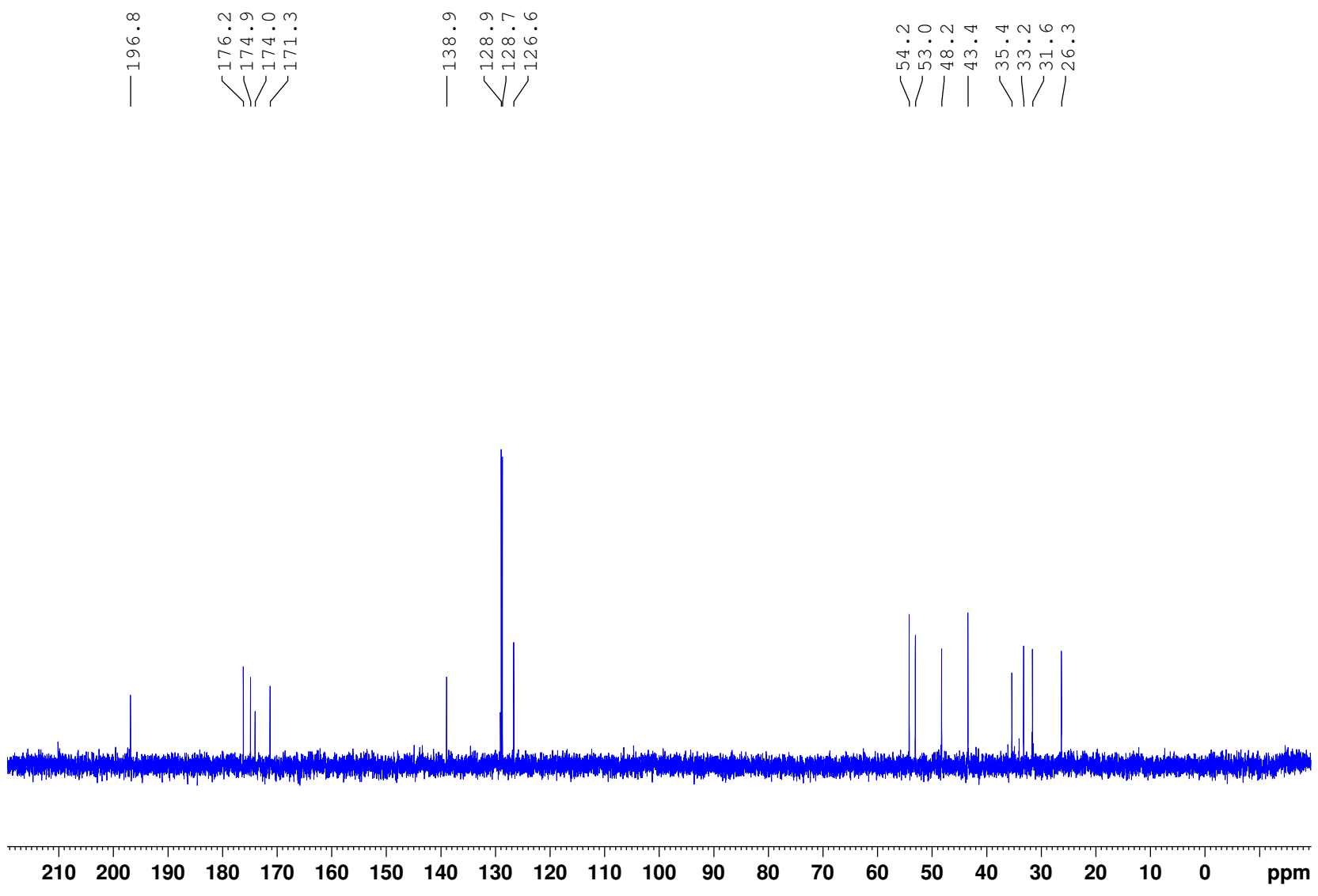


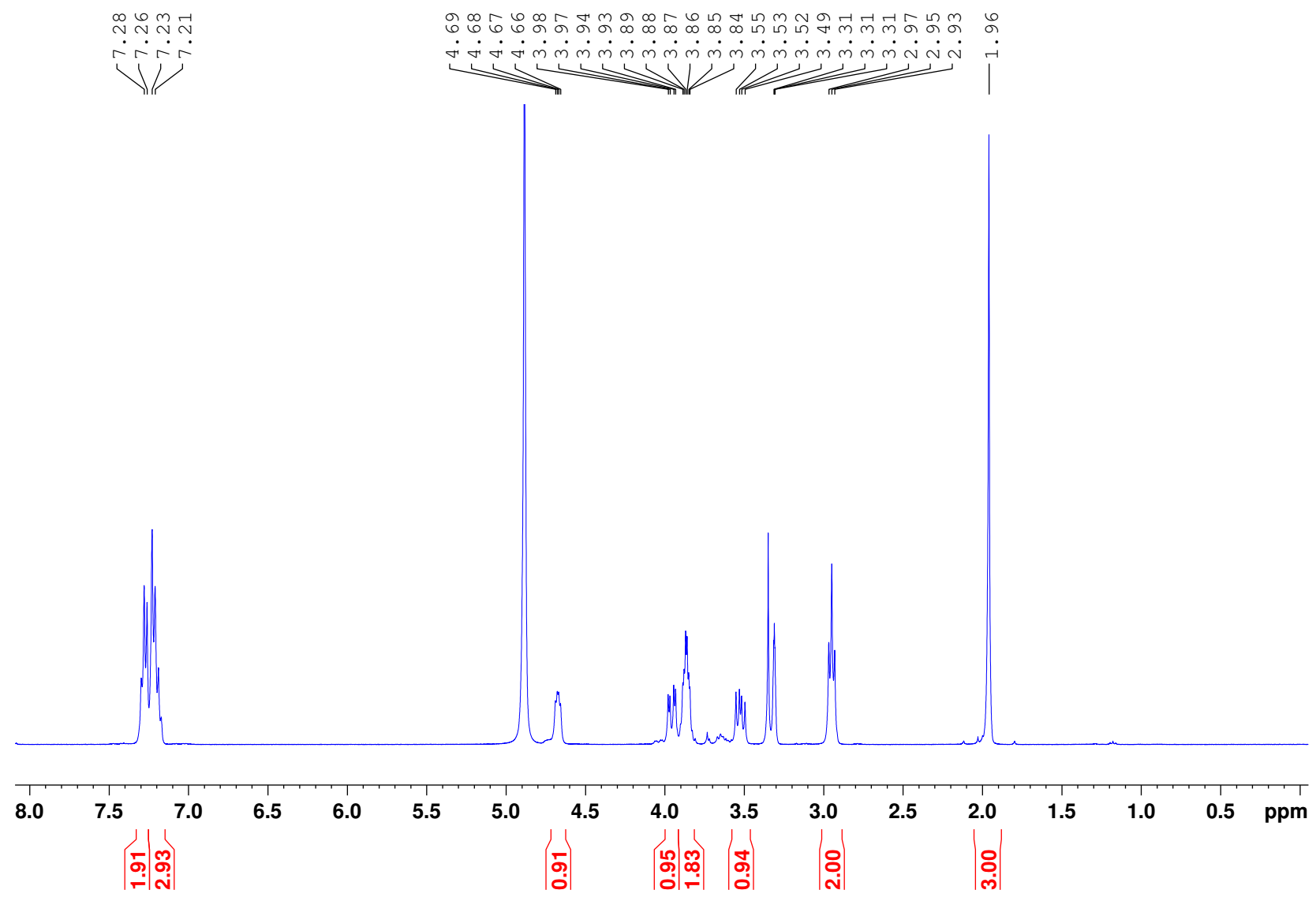


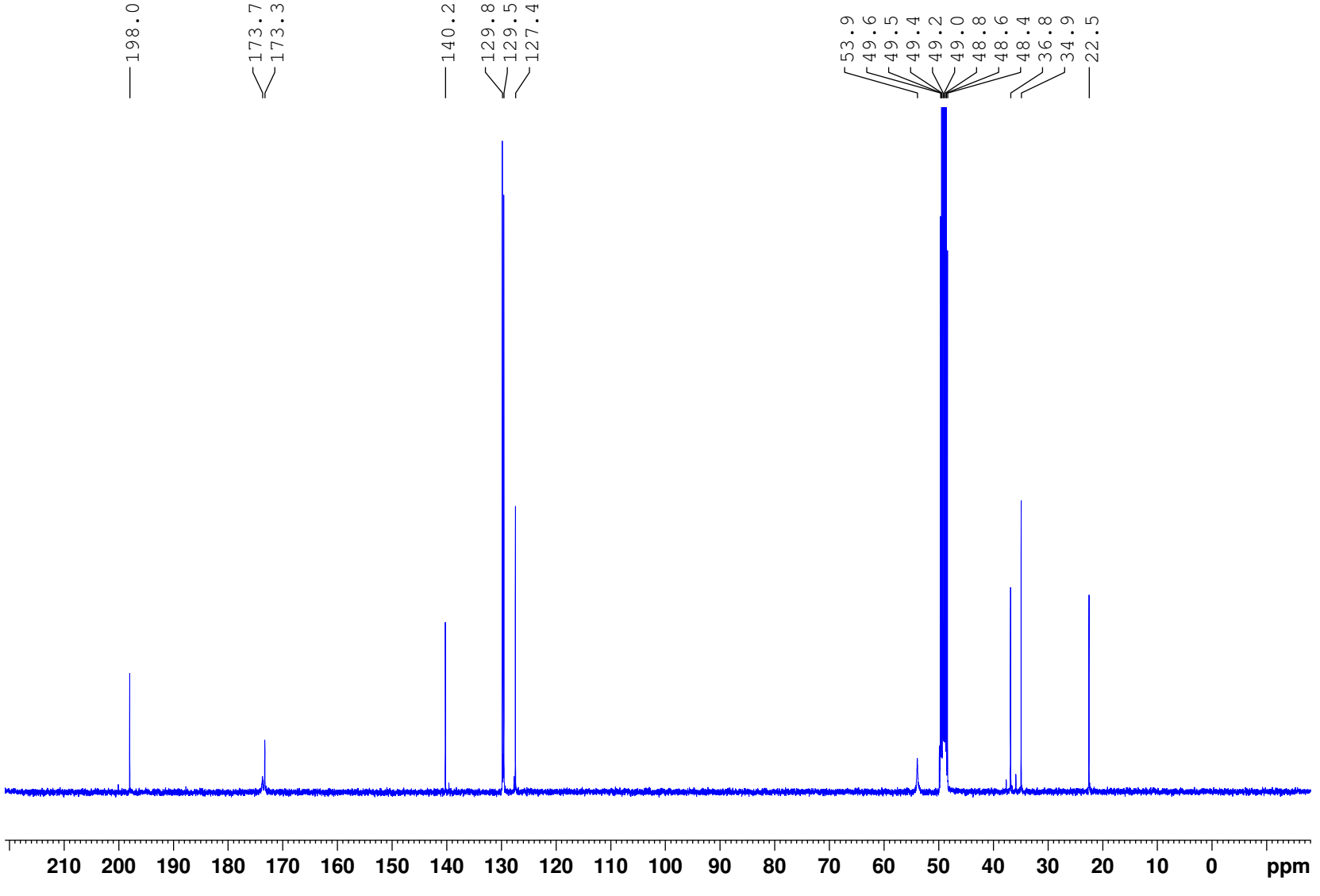


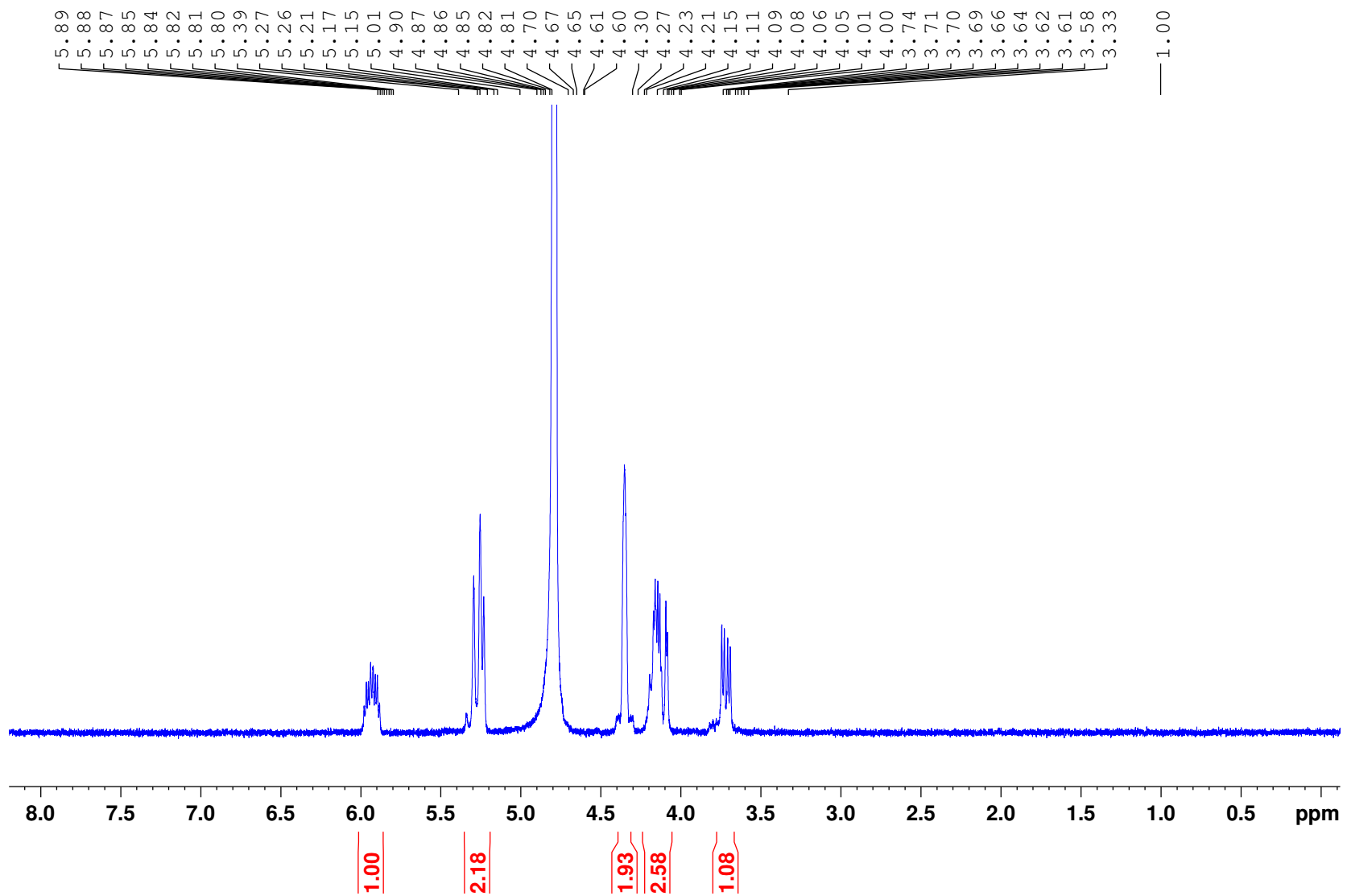


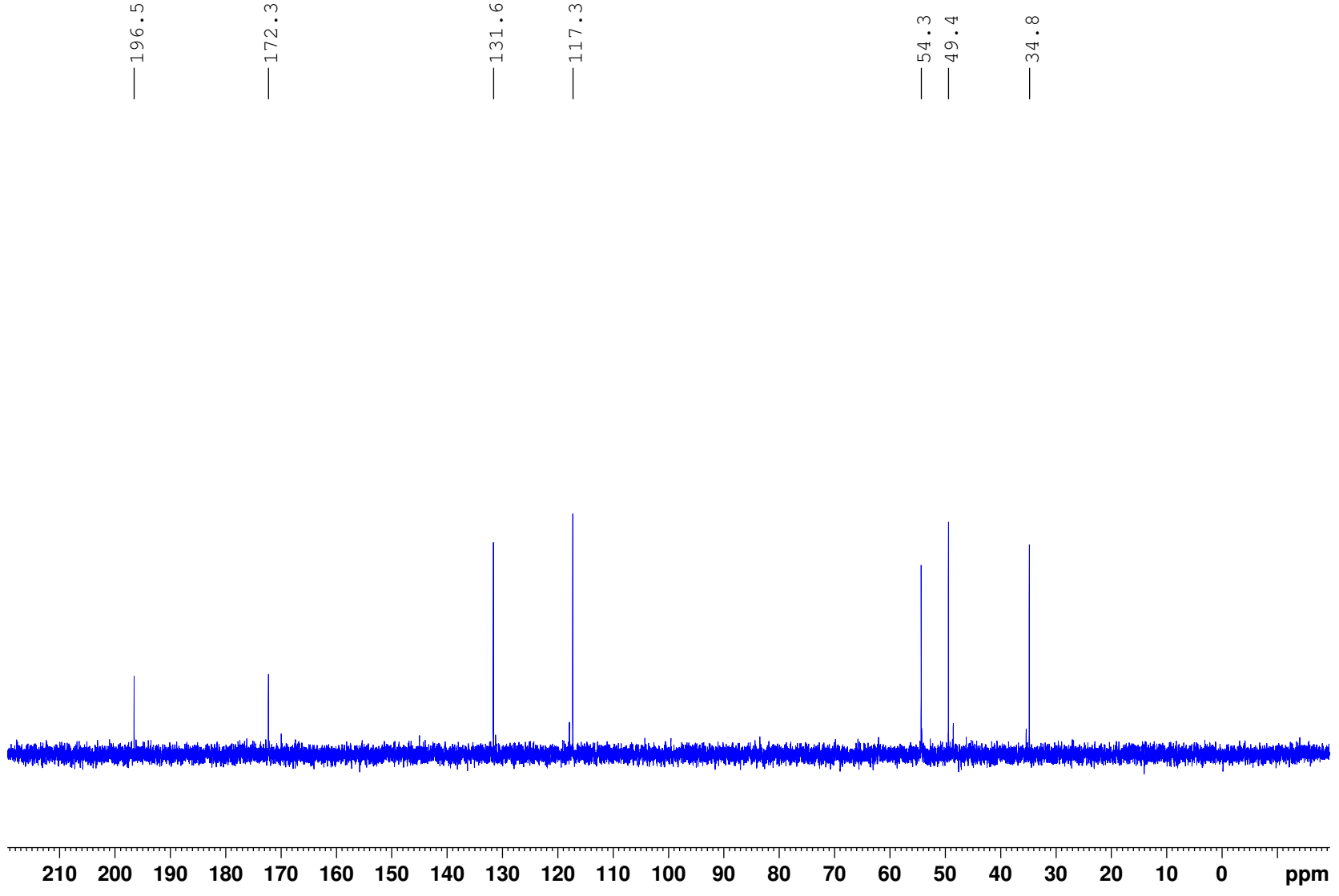


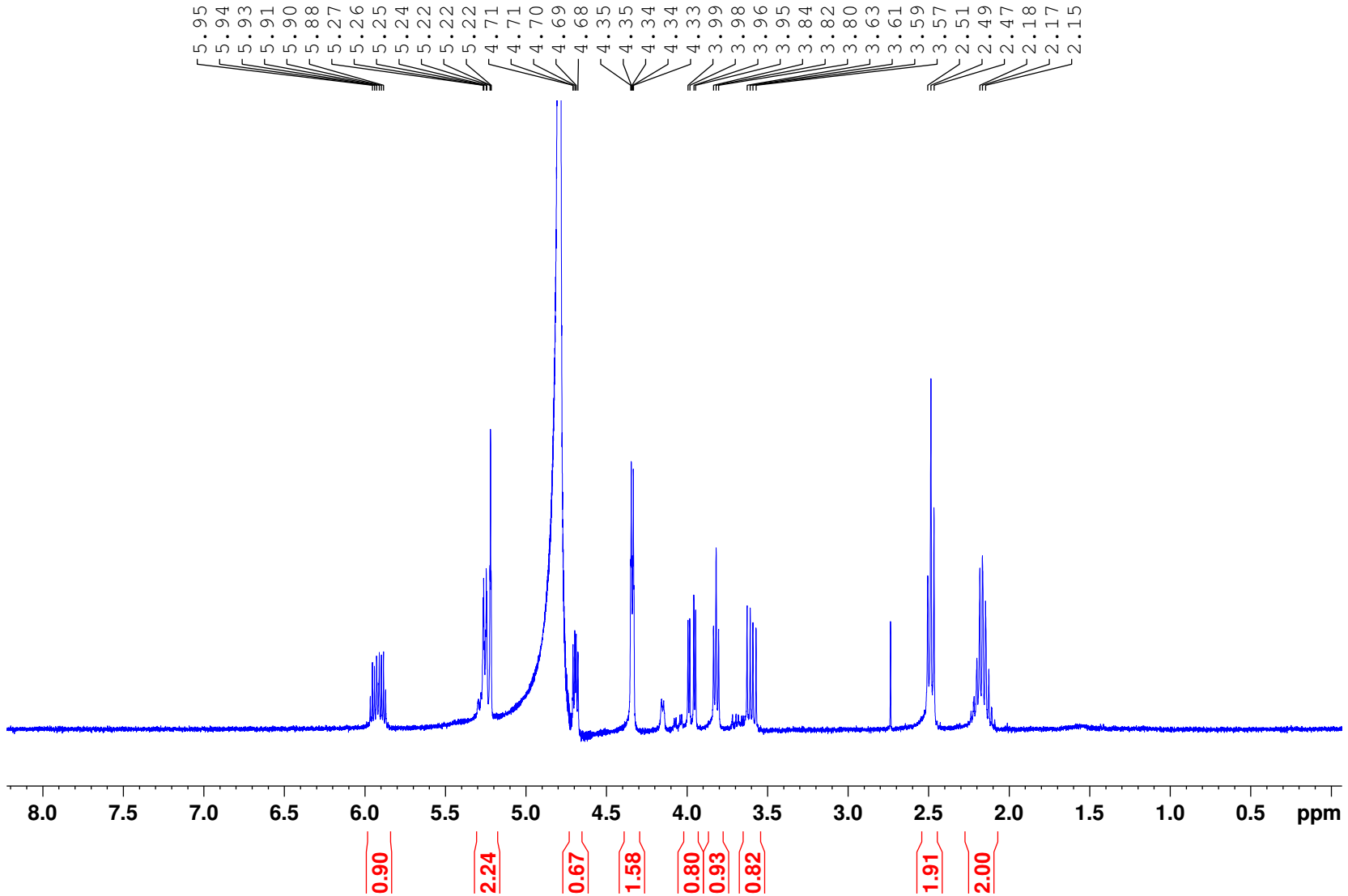


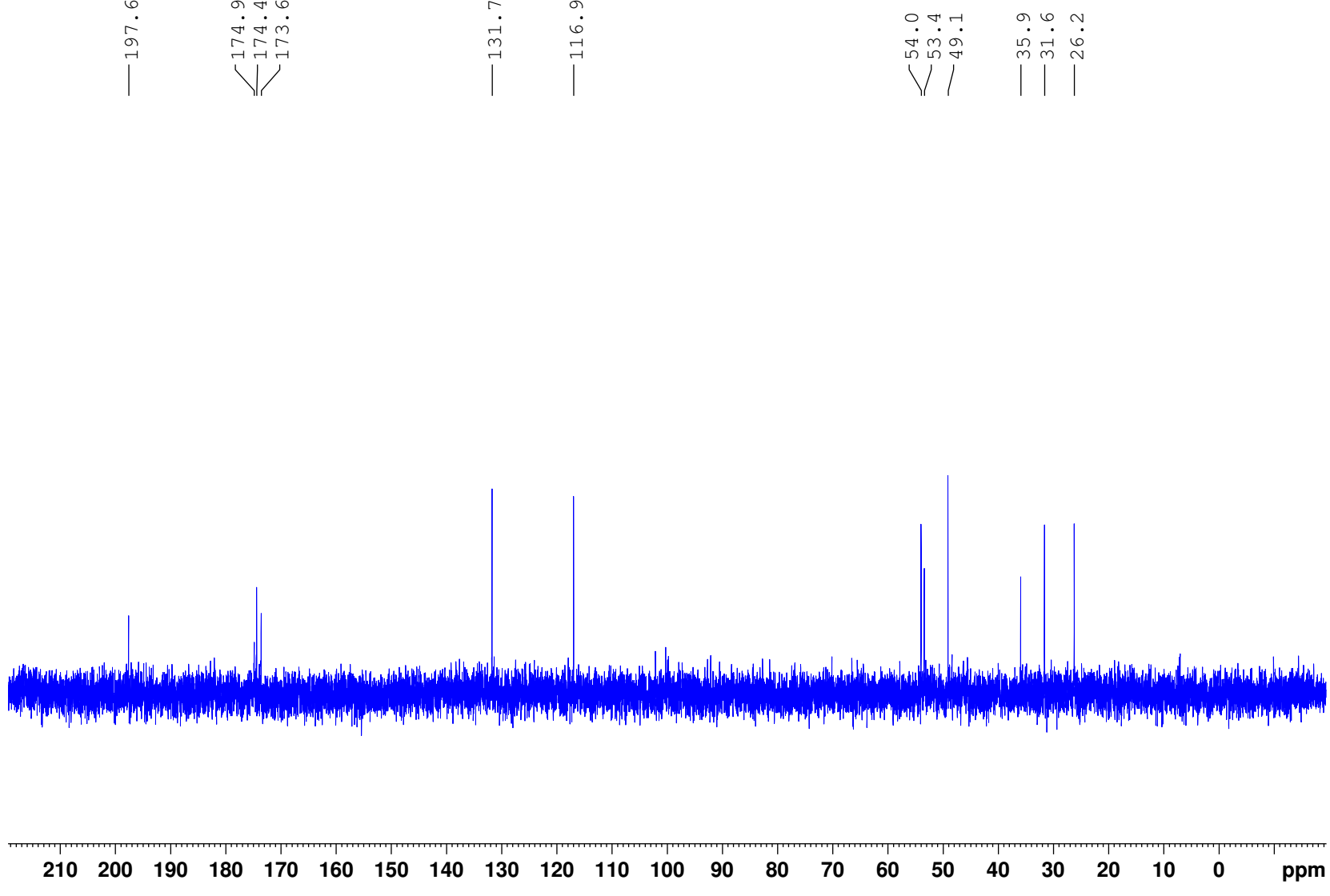


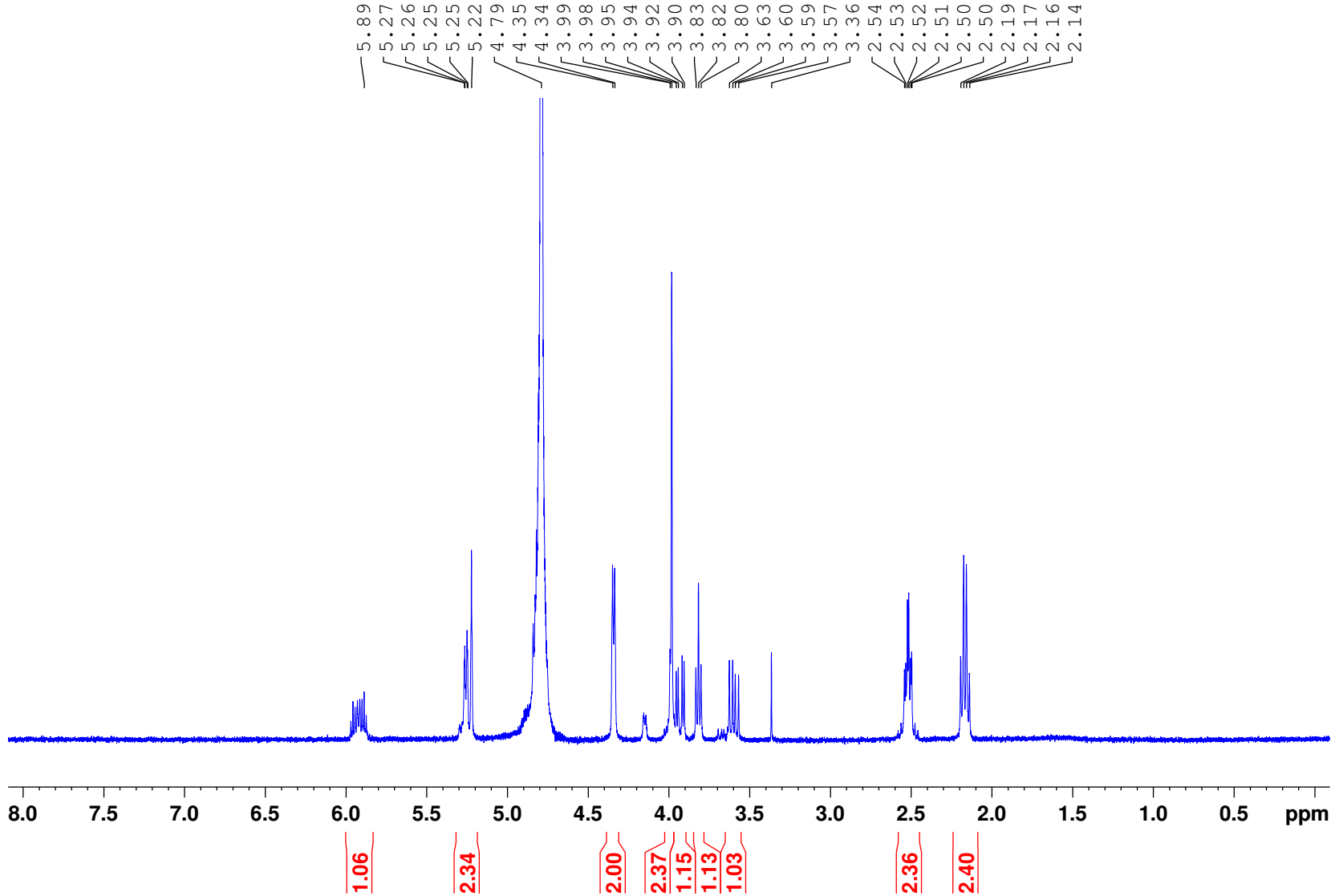


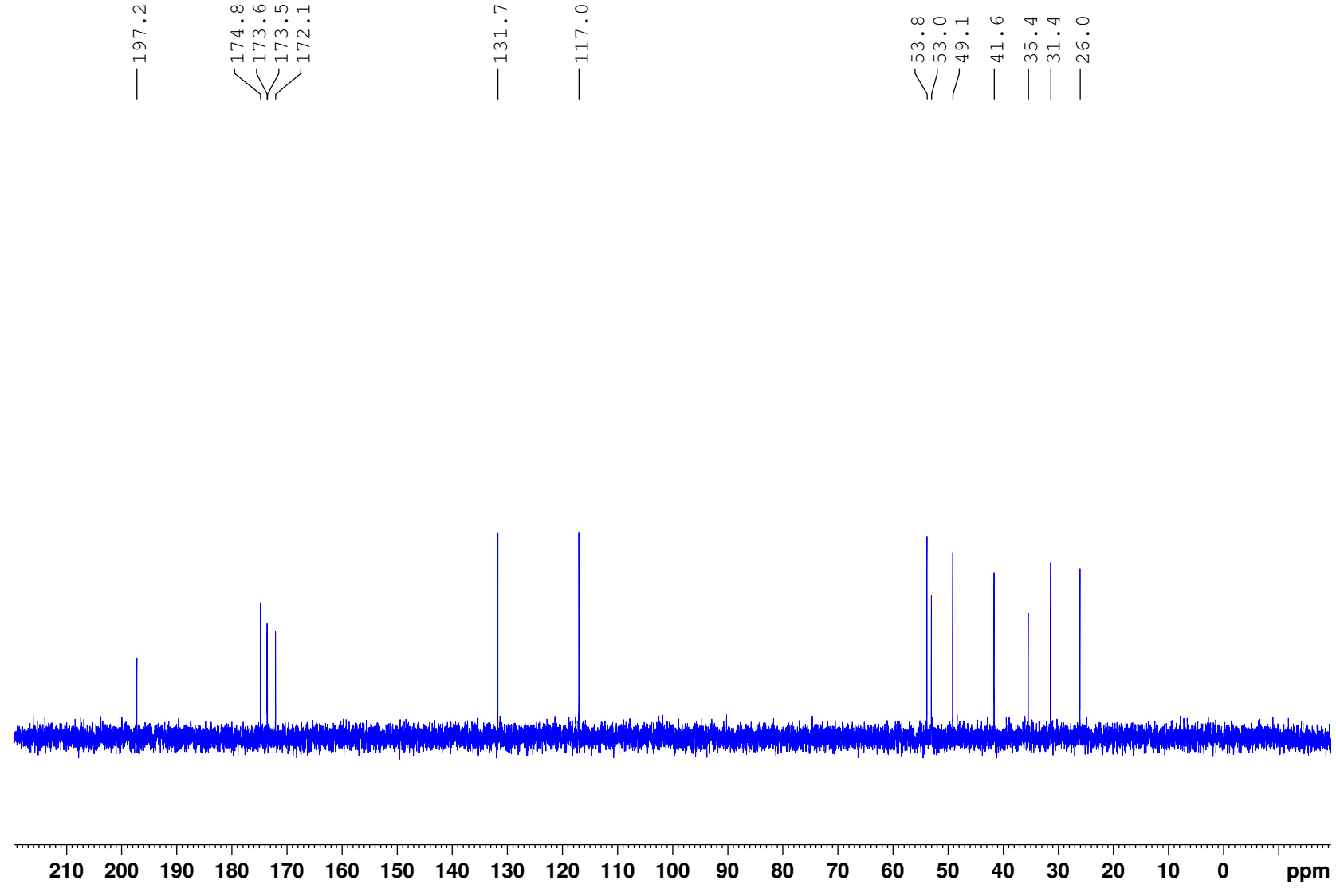


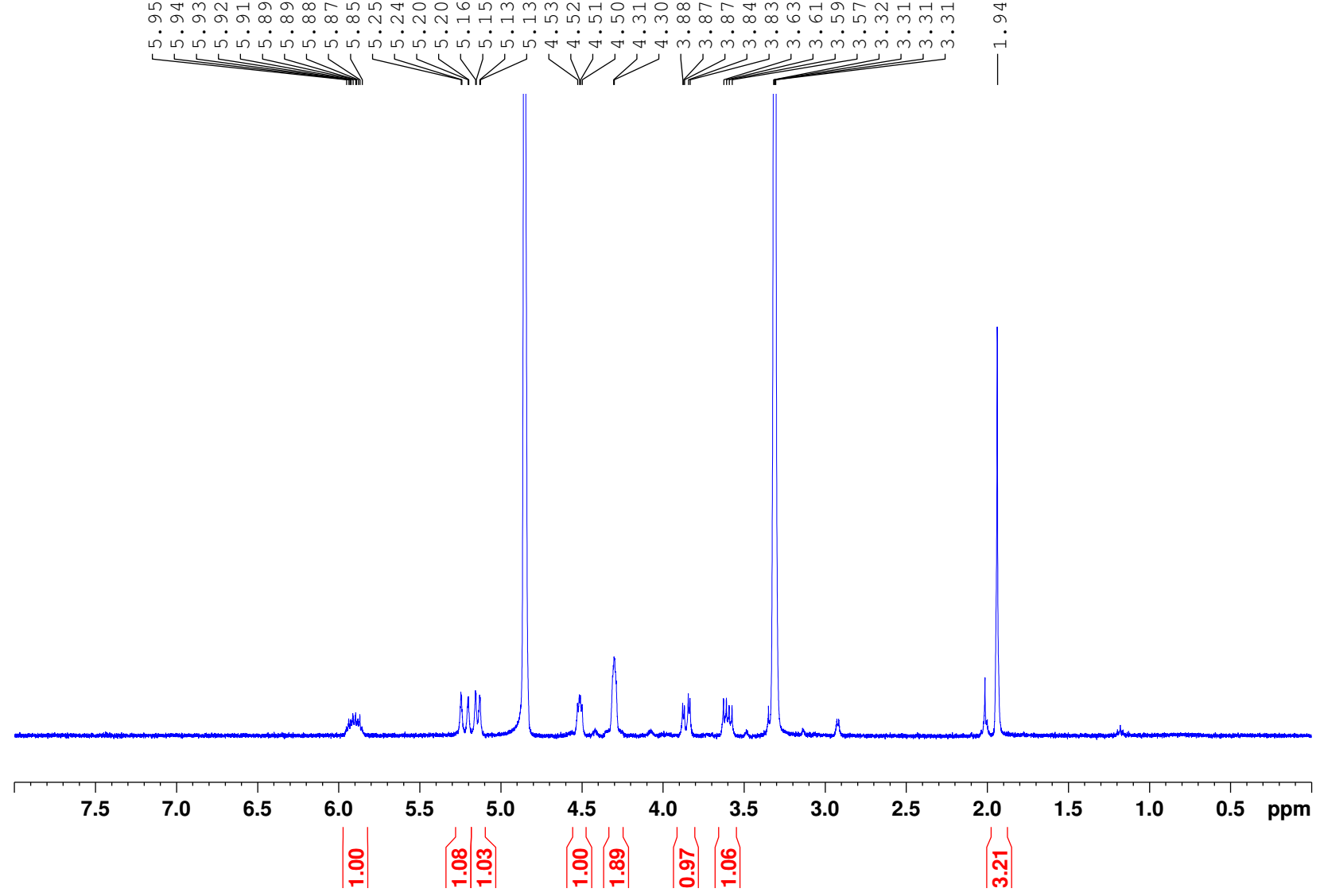


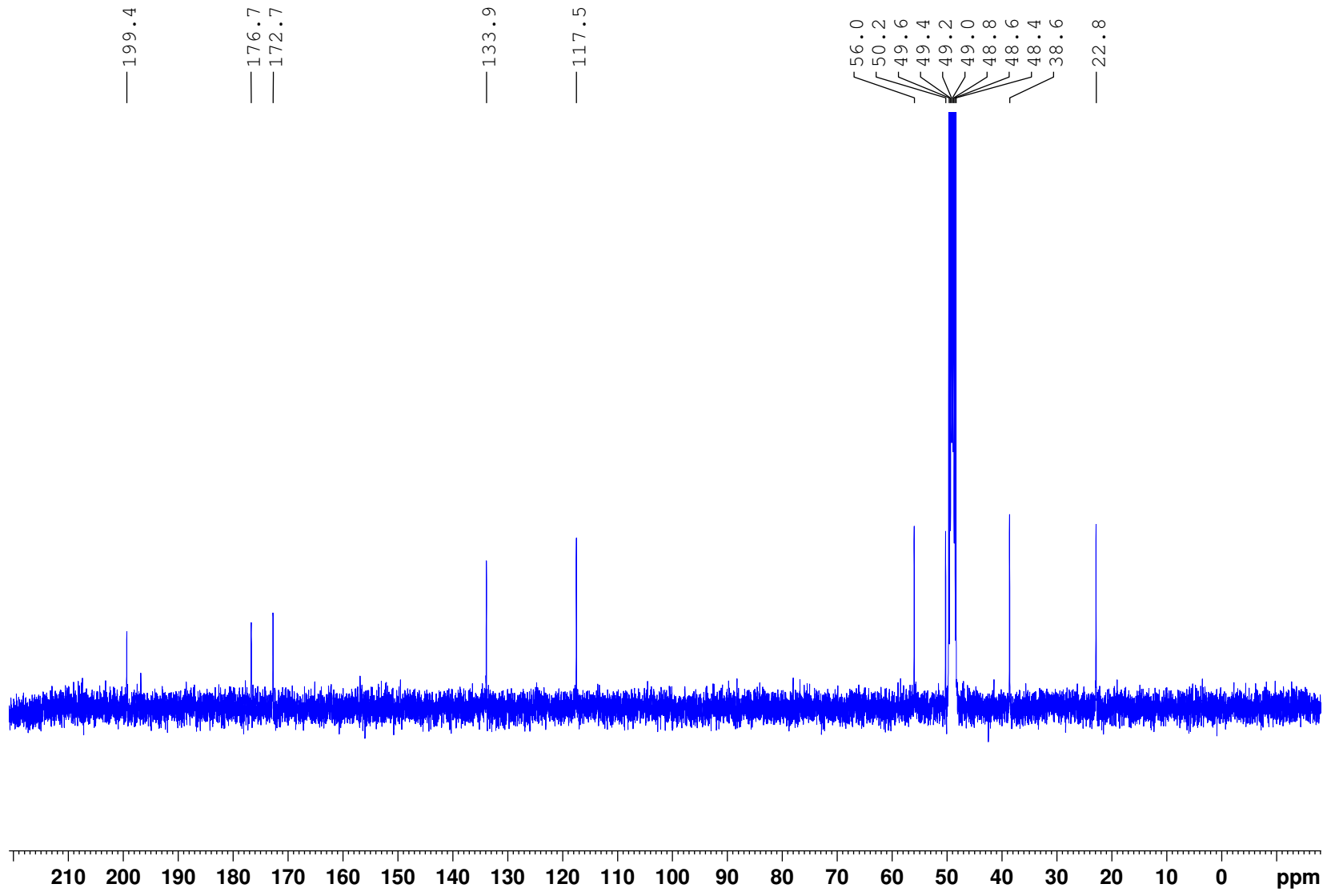


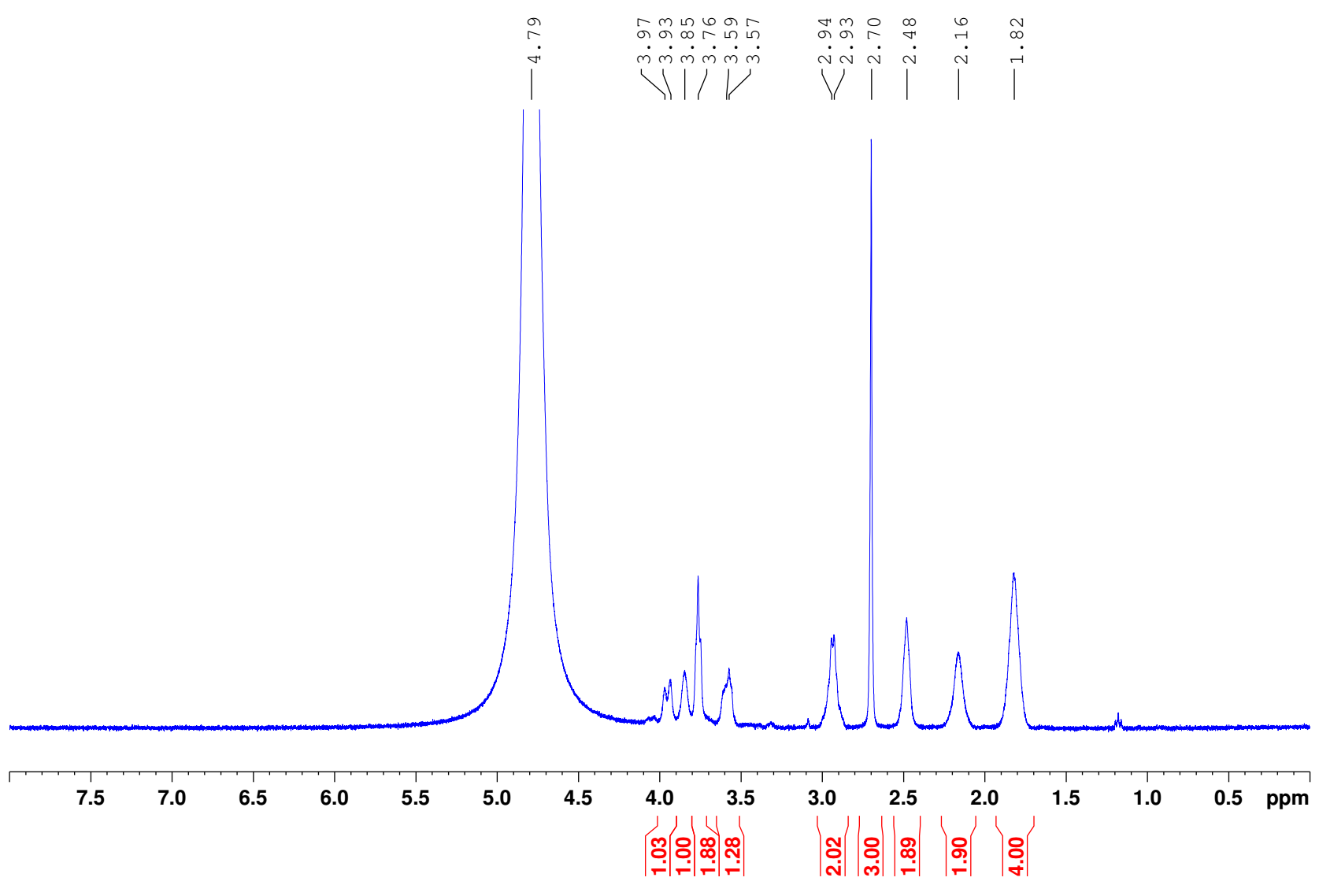


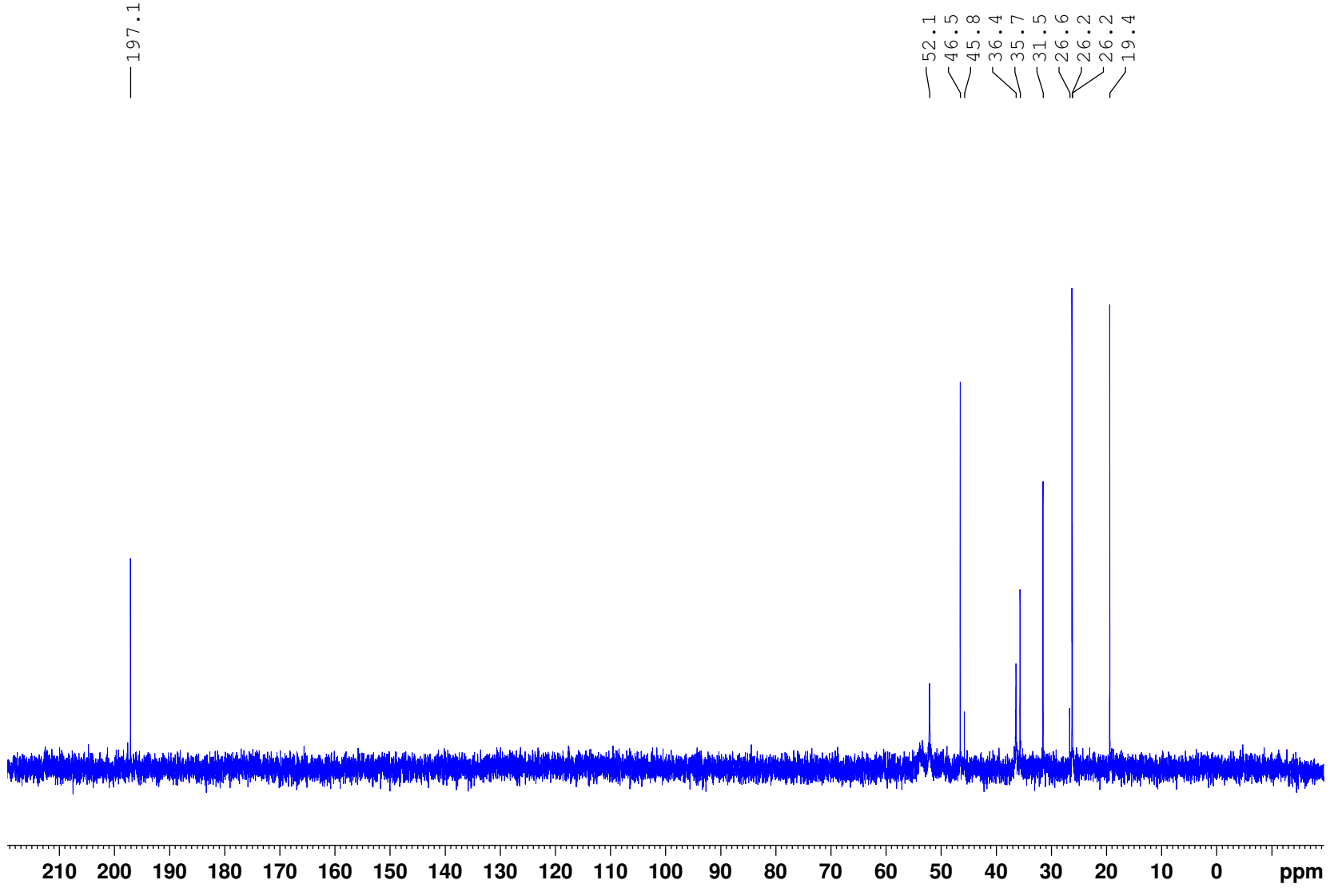


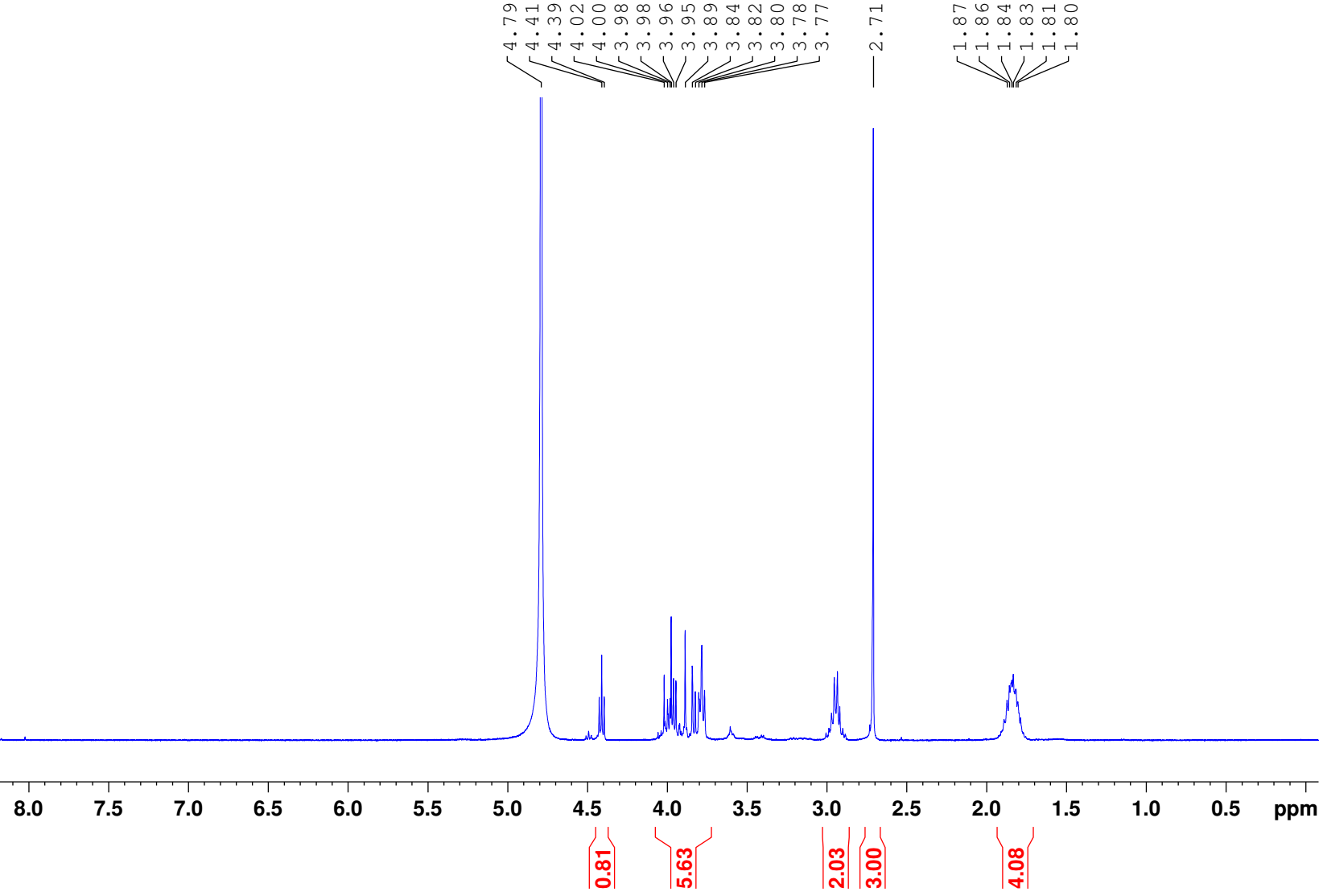


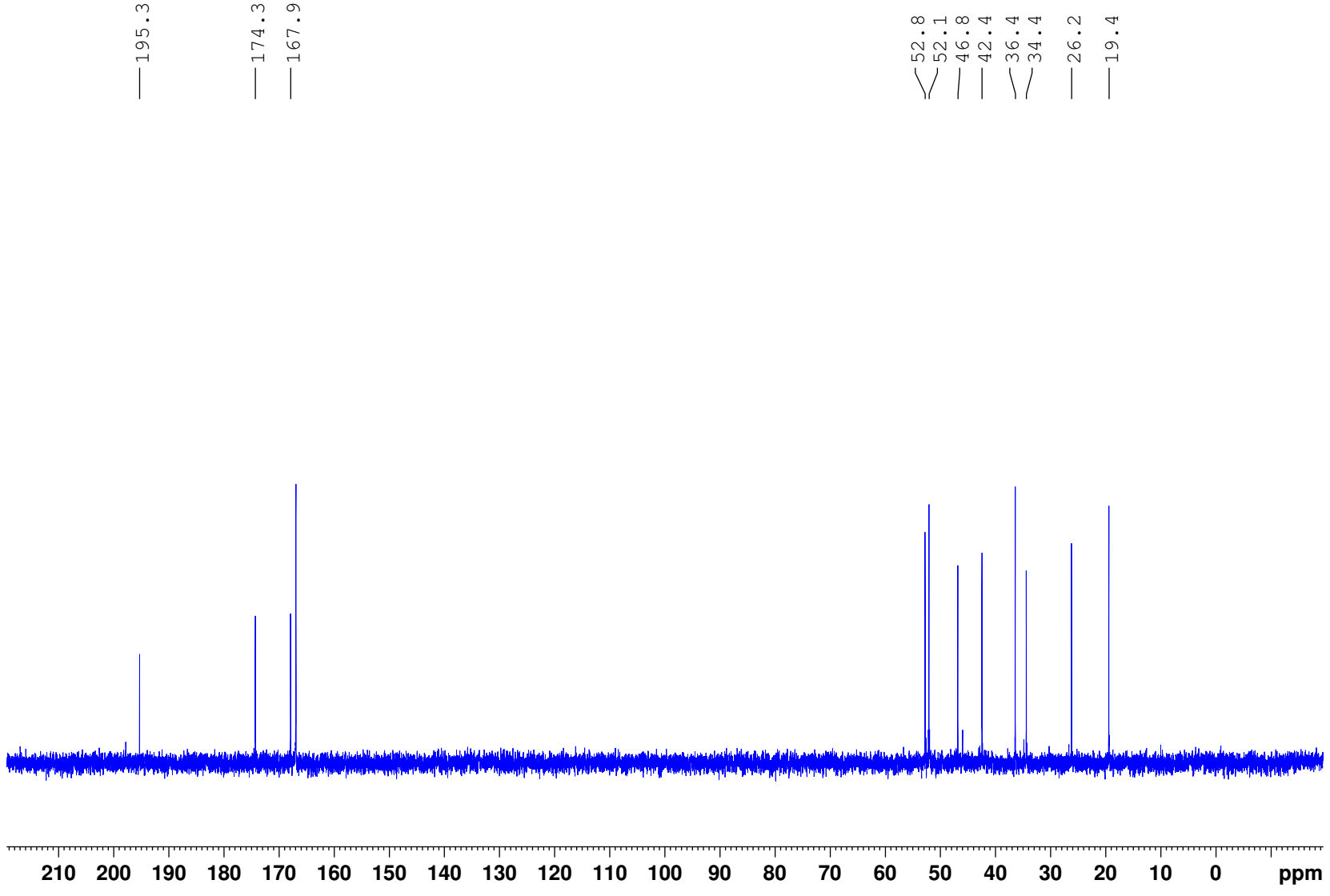


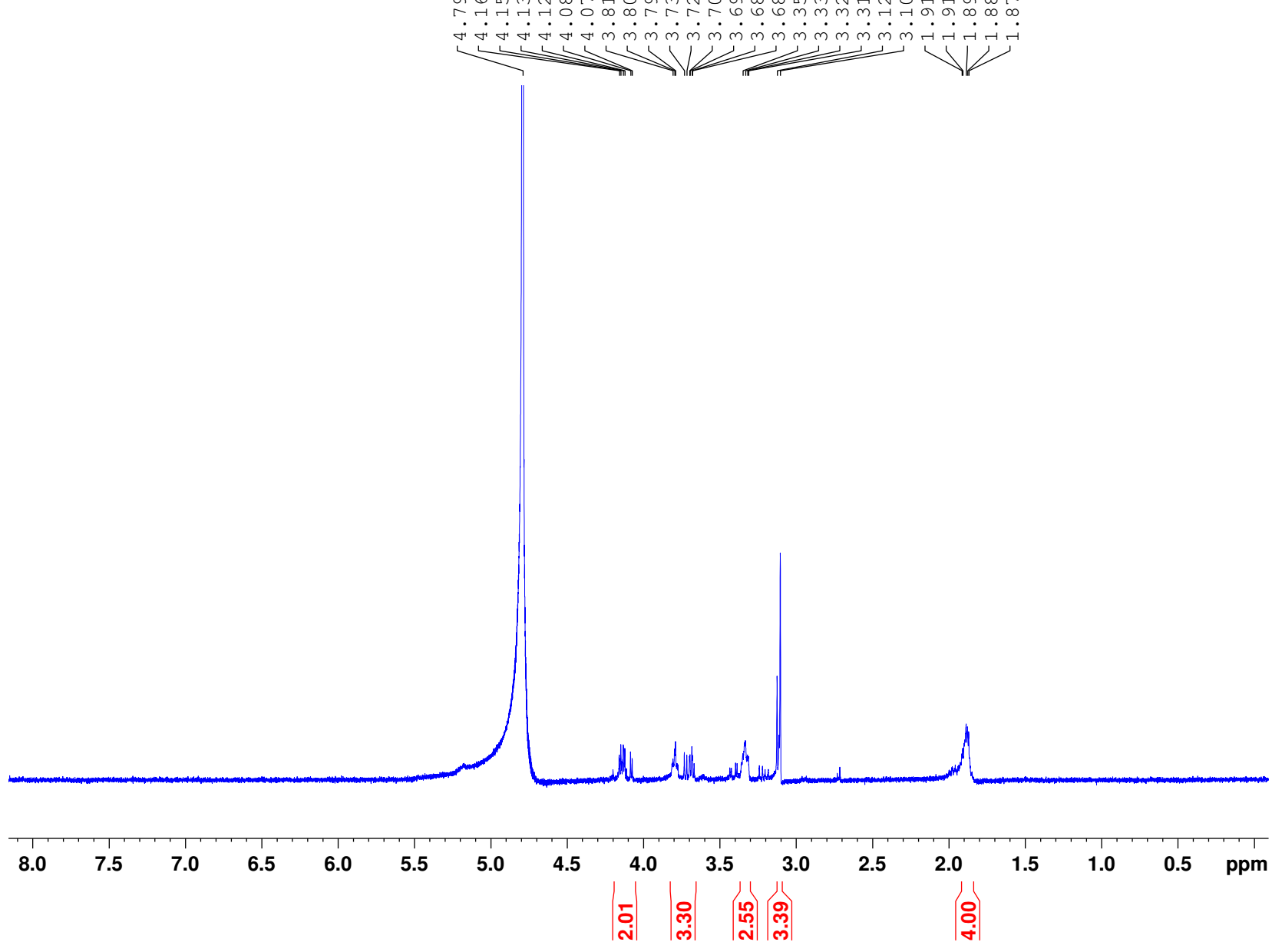


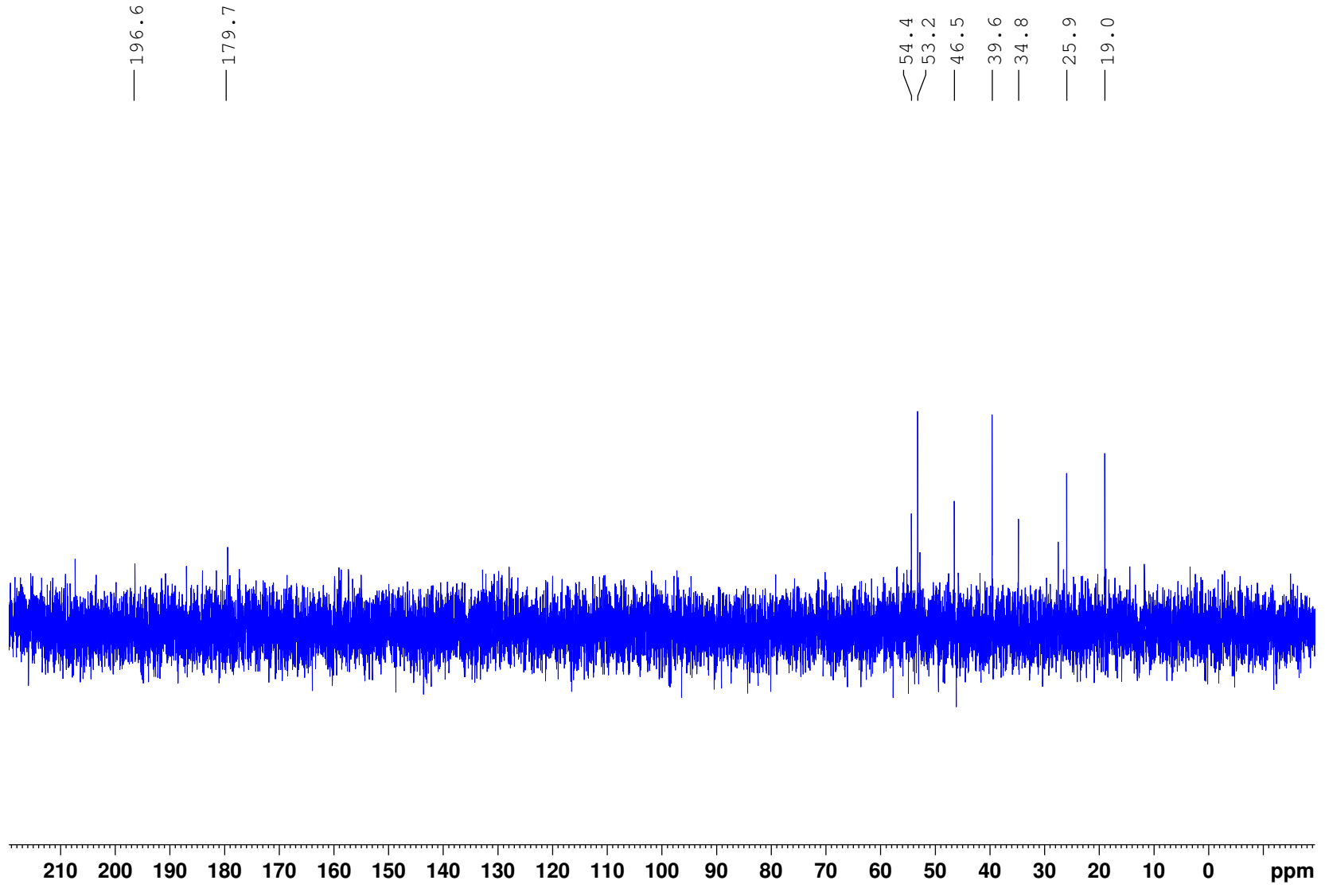


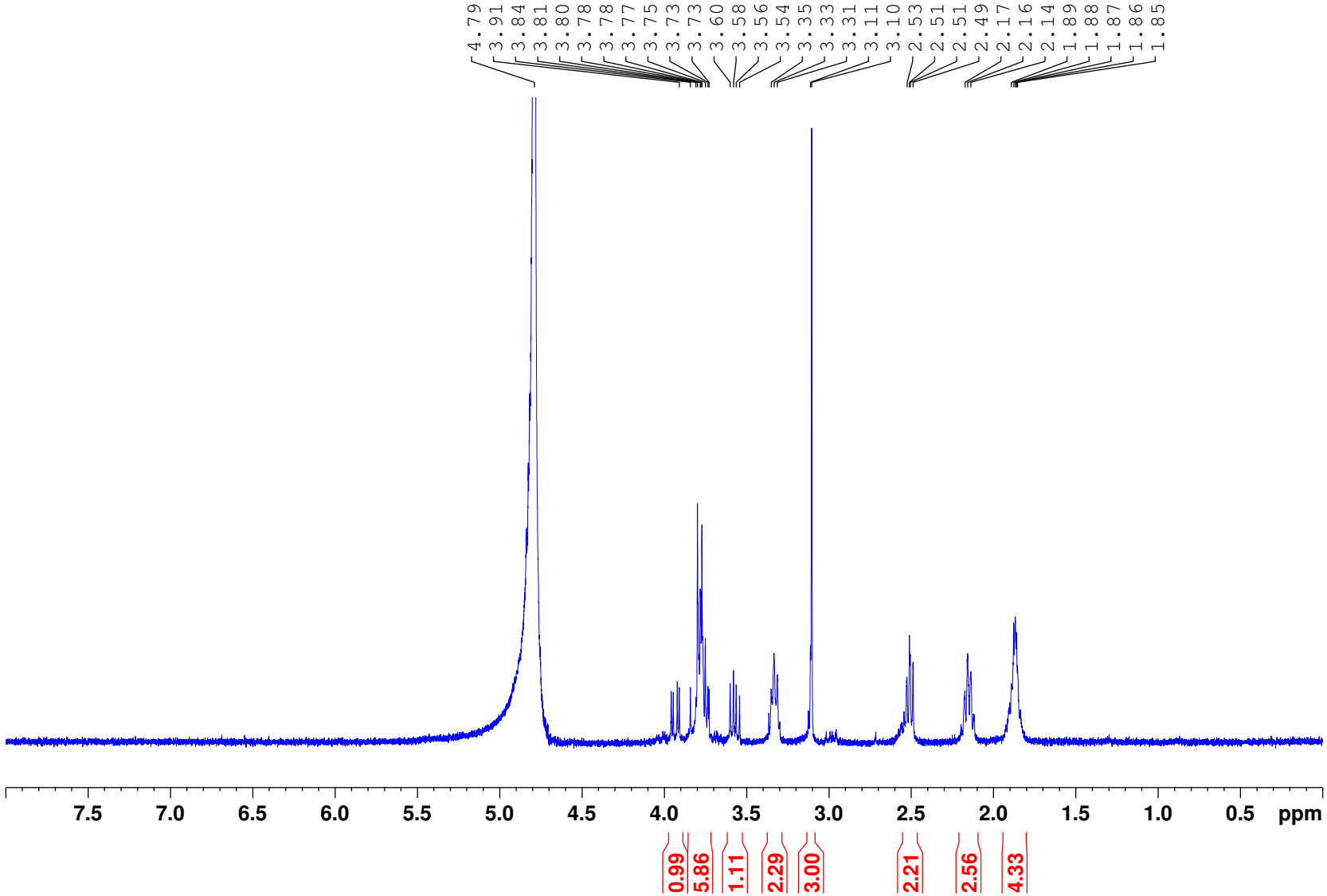


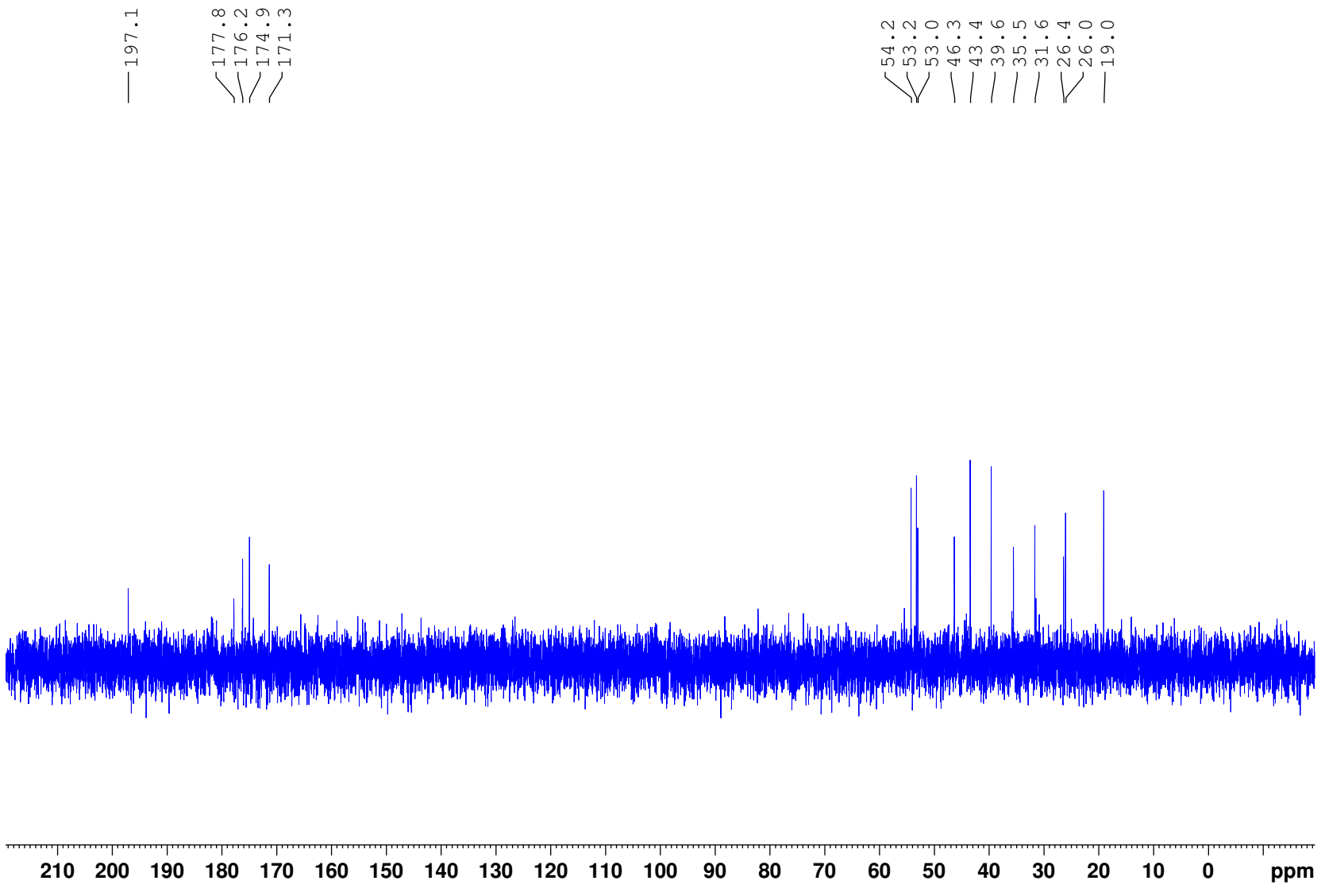


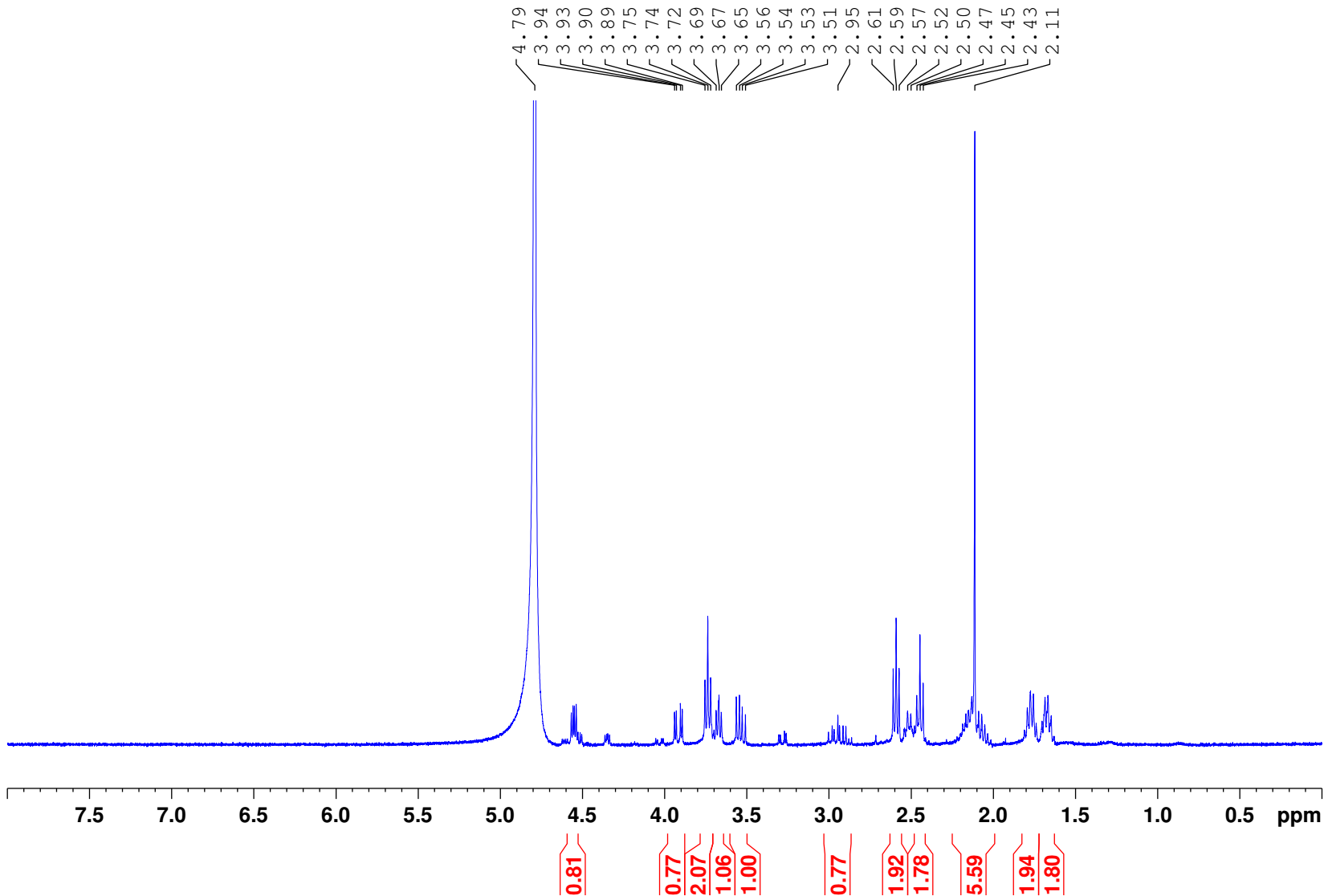


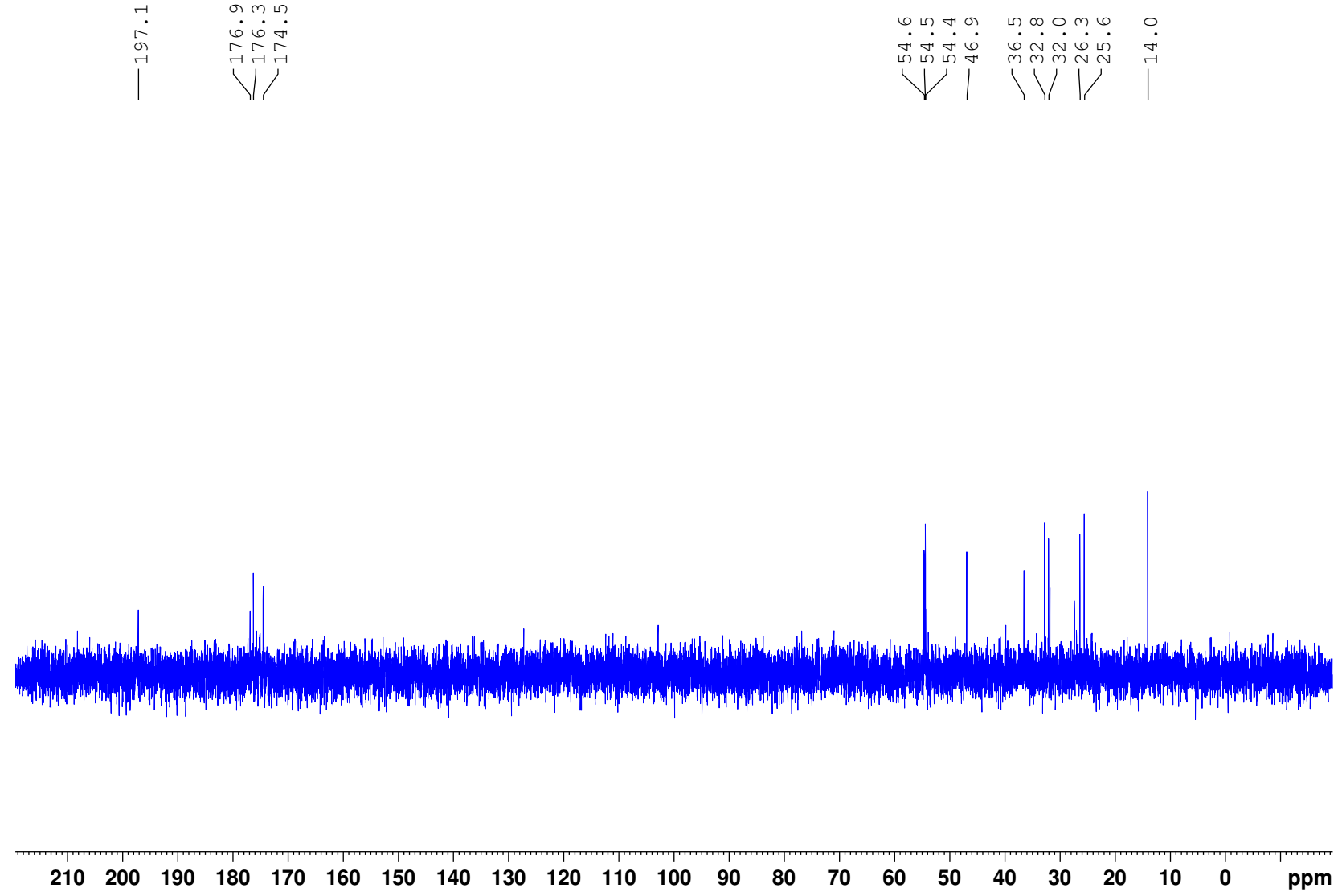


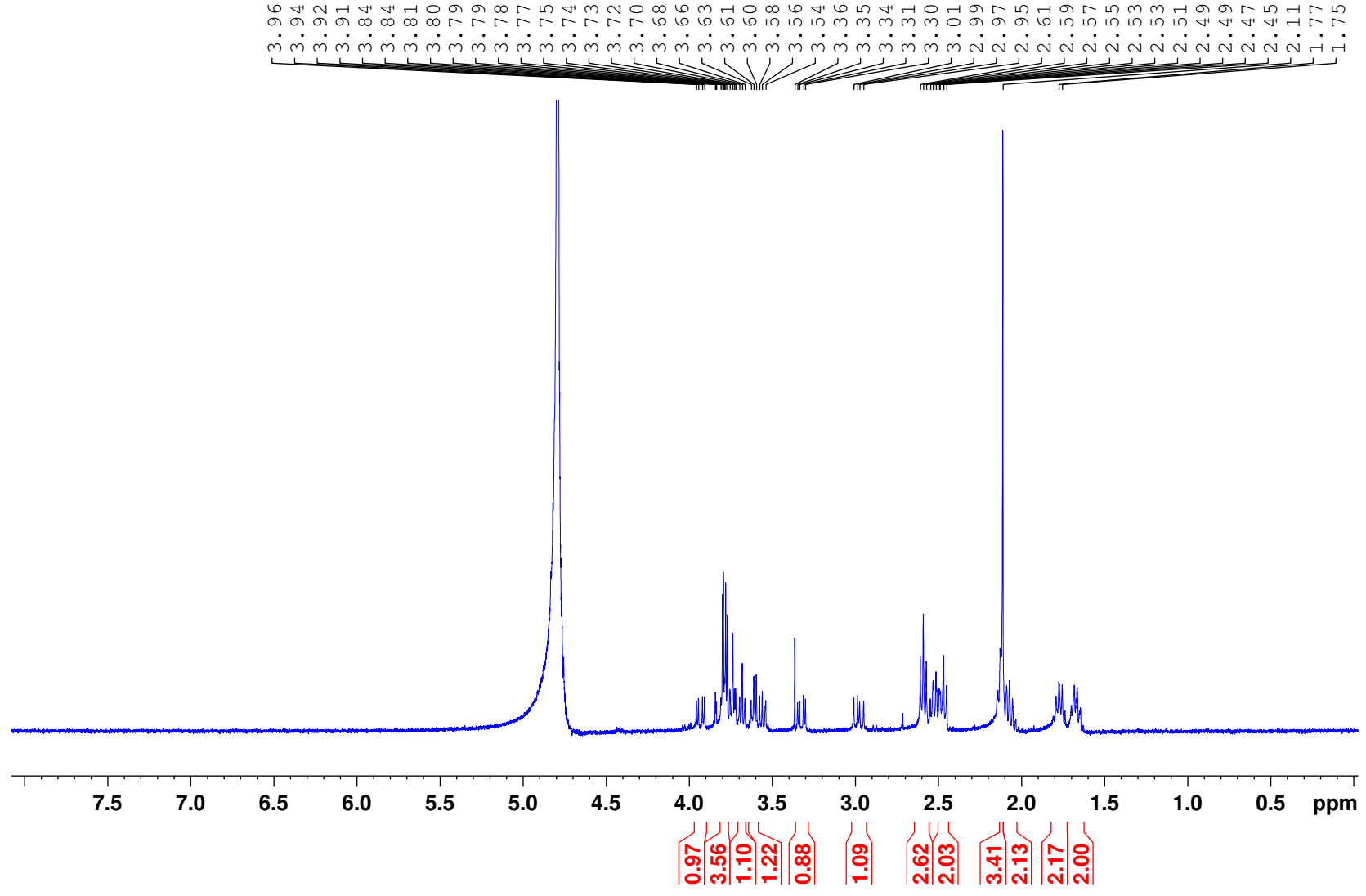


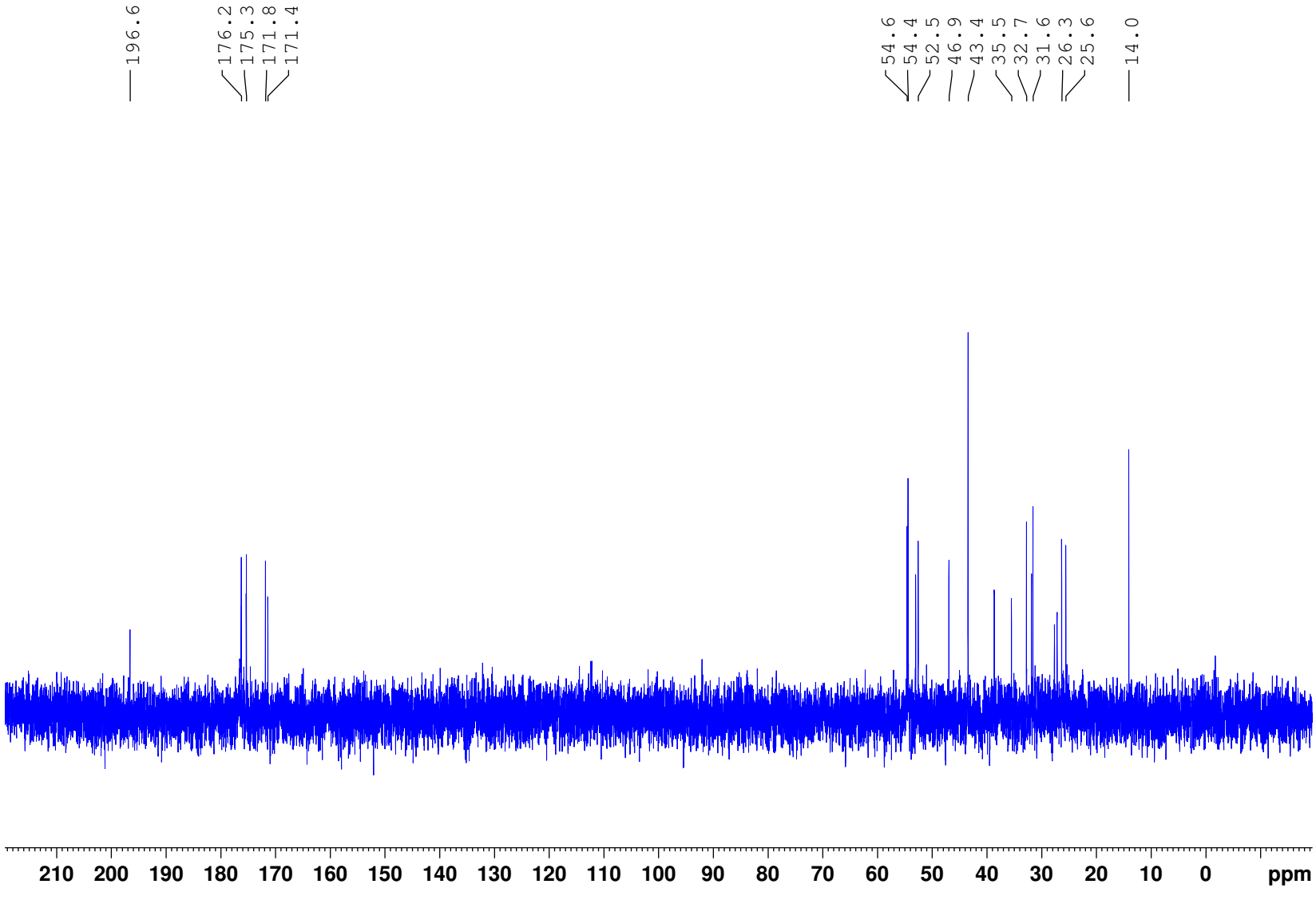

Supplement: Supplementary file 2 [file 41396_2023_1480_MOESM2_ESM.docx]
