# Supplementary material for: Metabolism of plant-derived toxins from its insect host increases the success of the entomopathogenic fungus Beauveria bassiana
Source: ISME J. 2023 Jul 21;17(10):1693–704. doi: 10.1038/s41396-023-01480-3 (PMC10504261; doi:10.1038/s41396-023-01480-3)
Supplement: Supplementary file 3 [file 41396_2023_1480_MOESM3_ESM.docx]

**Metabolism of plant-derived toxins from its insect host increases the success of the entomopathogenic fungus *Beauveria bassiana*, by Sun, R. *et al*.**

**Supplementary methods**

**Fungus, insects and plants**

*Brassica napus*, *Brassica nigra*, *Brassica oleracea*, and *Nasturtium officinale* plants, *Arabidopsis* *thaliana* land race Columbia-0 (Col-0) accession plants (with wild-type glucosinolates (GSLs)), and transgenic *myb28myb29* knockout mutant plants (without aliphatic glucosinolates) [1] were used. Plants were grown in a climate-controlled short-day environmental chamber at 21°C, 60% relative humidity, and a 14:10 h light:dark photoperiod. Colonies of the cabbage aphid *Brevicoryne brassicae* were generously provided by Dr. Rieta Gols (Wageningen University & Research, Wageningen, Netherlands), and were fed on *B. nigra* plants and maintained in a climate-controlled long-day environment chamber at 21°C, 60% relative humidity, and a 16:8 h light:dark photoperiod. *Beauveria bassiana* (ATCC74074) was purchased from E-Nema GmbH (Schwentinental, Germany) and incubated on potato dextrose agar (PDA) plates at 25°C. The related *B. bassiana* infection experiments were conducted in a controlled long-day environmental chamber at 25°C, 60% relative humidity, and a 16:8 h light:dark photoperiod.

**Collection of *B. bassiana* conidia and aphid infection**

*B. bassiana* conidia were collected from cultures grown on PDA over one week at 25°C. Sterile water (5 mL) was added to each plate and the plate scraped gently with a flat weighing spoon to detach conidia. The water was then passed through a filter (Acrodisc PSF with 10 µm pore size Versapor membrane, Pall Corporation, NY, USA) to obtain a conidial suspension. The concentration of conidia per mL was calculated based on counts made from 10 µL of a 1:100 dilution placed in a Neubauer hemocytometer counting chamber (Paul Marienfeld, Lauda-Königshofen, Germany). Conidial concentrations were then adjusted in milliQ water based on the hemocytometer calculation to achieve 10^8^ conidia per mL for our experiment.

To efficiently infect the cabbage aphid with *B. bassiana*, 300 µL of conidia (10^8^ conidia per mL) were spread evenly on freshly prepared PDA plates, and the plates incubated at 25°C for 7 days to allow sufficient additional conidia to form. Aphids from each treatment were then allowed to crawl on the conidia plates for a half hour en masse with 6 replicates for each plant species and treatment [2]. This resulted in a dose of approximately 1 x 10^6^ conidia per aphid. Having aphids crawl on conidia plates was found to be a more effective means of infection than spraying them directly with a spore suspension. Aphids that crawled on plates without *B. bassiana* were used as the uninfected control groups (N=6 for each treatment).

**Development of *B. bassiana* on the cabbage aphid**

The survival of the cabbage aphid after *B. bassiana* infection was investigated on different crucifer host plants. Aphids were allowed to feed on uninfected leaves of *B. napus*, *B. nigra*, *B. oleracea*, *N. officinale,* or *A. thaliana* Col-0 wild-type and transgenic *myb28myb29* knockout mutant plants (6 replicates per species) for two weeks to sufficiently sequester GLSs from their host plants. Subsequently, groups of forty 4^th^ instar nymph cabbage aphids were collected from each plant and infected with *B. bassiana* en masse by crawling on a plate covered with conidia (see previous section)*.* Forty 4^th^ instar nymphs not infected by *B. bassiana* were used as a control (6 replicates for each plant species). After infection, the infected and non-infected cabbage aphids were transferred to fresh plants (40 aphids per plant, and 6 plants for each species; two treatments (infected and not infected aphids) per plant species, totaling 12 plants per plant species). The numbers of surviving adult aphids (the 4^th^ instar nymphs turned into adults after 5 days of development) and their offspring were counted on each plant five days post infection. The total number of surviving adult aphids plus their offspring on each plant was calculated by adding the two figures above. The survivorship of infected aphids was calculated based on the surviving adults compared to the initial number (40) of aphids applied. Numbers of surviving adults and their offspring are reported in Table S11.

**GLSs in the cabbage aphid sequestered from the host plant**

To quantify the accumulation of GSLs from host plants by the cabbage aphid, the GSL contents of both the cabbage aphids and their host plants were chemically analyzed. Leaves from *B. napus*, *B. nigra*, *B. oleracea*, *N. officinale*, and *A. thaliana* plants were infested with 4^th^ instar aphid nymphs. On the 14^th^ day post–infestation, the cabbage aphids from infested plants, as well as non-infested control plants, were collected and immediately frozen in liquid nitrogen for GSL analyses. Five replicates were used for each treatment. The samples were freeze-dried (ALPHA 1-4 LDplus freeze dryer, Martin Christ, Osterode am Harz, Germany) thoroughly for 2 days, and homogenized by shaking with 5-6 metal balls (3 mm) in each tube.

GLSs were extracted from samples (approximately 10 mg leaves and 5 mg aphids) using 1 mL extraction solvent (80% aqueous methanol; v:v) with 50 mM sinalbin as an internal GSL standard [3]. After 5 min incubation on a horizontal shaker (230 rpm) with solvent, supernatants were collected by centrifugation (13,000 x g at 4°C for 30 min). Next, 800 µL of each supernatant were loaded on DEAE-Sephadex A-25 columns (28mg; Sigma-Aldrich, Munich, Germany). The GLSs bound on DEAE-Sephadex were desulfated by treatment with 30 µL sulfatase (for preparation of sulfatase solution see Graser et al., 2000 [4]) overnight at room temperature. The next day, desulfo-GSLs were eluted with 500 µL milliQ water for HPLC-UV measurement. Desulfo-GSLs were analyzed on an Agilent Technologies 1100 Series HPLC (Agilent Technologies, Santa Clara, CA, USA) with a diode-array detector using a Nucleodur Sphinx RP column (250 × 4.6 mm × 5 mm, Macherey-Nagel, Düren, Germany). Desulfo-GSLs were detected at 229 nm and quantified using molar response factors according to Burow et al., 2006 [5]. Water and acetonitrile were employed as mobile phases A and B, respectively. The elution profile was: 0-1 min, 1.5% B; 1-6 min, 1.5-5% B; 6-8 min, 5-7% B; 8-18 min, 7-21% B; 18-23 min, 21-29% B; 23 -23.1 min, 29-100% B; 23.1-24 min, 100% B; 24-24.1 min, 100-1.5% B; 24.1-28 min, 1.5% B, at a flow rate of 1.0 mL/ min.

**Transcript abundance for measuring *B. bassiana* infection and *gst* expression**

To quantify *B. bassiana* infection on cabbage aphids fed on different crucifer plants, infected and non-infected aphids prepared as above were collected on the 5^th^ day post infection. Total RNA was isolated from aphids by using the RNeasy plant mini kit (Qiagen, Hilden, Germany) and genomic DNA contamination was eliminated by RNase-free DNase (Qiagen). The quantity and quality of RNA in each sample were determined with a NanoDrop 2000c instrument (Thermo Fisher Scientific). cDNA was synthesized using SuperScript III Reverse transcriptase kits (Invitrogen, Waltham, MA, USA). Afterwards, qPCR was performed to determine the relative transcript abundance of *B. bassiana actin* (N=5 for each treatment) using Brilliant III Ultra-Fast SYBR Green QPCR Master Mix (Agilent Technologies). The aphid *EF1α* gene was used as an internal control to normalize the abundance of *B. bassiana* *actin* gene transcripts.

To examine the inducibility of *Bbgst* genes by ITCs, *B. bassiana* cultures were incubated in PDB with 50 µM ITC (dissolved in 0.025% aqueous ethanol) for 4 h in a shaker at 25°C and 180 rpm. *B. bassiana* incubated in PDB with solvent alone (0.025% aqueous ethanol) was used as a negative control (N=5 for each treatment). *B. bassiana* was harvested by centrifugation (13,000 x g at 4 °C for 30 min) and used for RNA isolation, cDNA synthesis and qPCR. *Bbgst* transcript levels were normalized to the *B. bassiana* *actin* gene. All gene accession numbers and primer pairs (designed via Primer3 software version 4.0) are listed in Table S8.

**Identification and classification of BbGST genes**

All seventeen putative GST-encoding genes were cloned from the *B. bassiana* ATCC74074 strain, based on the previously annotated *B. bassiana* ARSEF 2860 strain sequence [(assembly accession: ASM28067v1)](https://www.ncbi.nlm.nih.gov/genome/910?genome_assembly_id=31910). The full length *BbGST* mRNA sequences were cloned from the synthesized cDNA pool obtained from primer pairs FLF and FLR (Table S8), and their sequences have been submitted to NCBI (accession codes are listed in Table S6).

In order to classify the BbGSTs, the conserved domains within a protein or coding nucleotide sequence were searched in NCBI. GST proteins that contained the most similar N-terminal domain or C-terminal domain were identified. The closest hits of the BbGSTs to known GSTs are listed in Table S2. Phylogenetic analysis of BbGST proteins were performed on the amino acid sequences of the 17 GSTs. Sequences were aligned using Clustal Omega 1.2.2, and a UPGMA tree was generated using Geneious Prime. In addition to the 17 BbGSTs, the sequences of 60 other putative fungal GSTs were obtained from NCBI (Table S7).

**Recombinant BbGST expression and enzyme purification**

To determine the specific activities of *B. bassiana* GSTs with ITCs, BbGST proteins were heterologously expressed in *Escherichia coli* cells. Restriction enzyme cutting sites were added to the ends of the cloned full-length *BbGSTs* using the primer pairs BbgstVF and BbgstVR (Table S8), and the fragments were further digested by restriction enzymes (Thermo Fisher Scientific). The pET28a vector used to express the target proteins was also restriction digested. The restriction-digested *Bbgst* fragments were inserted into the restriction-cut pET28a cloning site by T4 DNA ligase (Invitrogen). The inserted vector was further transformed into *E. coli* BL21 (DE3) cells (Invitrogen) by chemical transfection. The selected colonies were incubated in 5 mL LB medium with 80 µg/mL kanamycin overnight at 37°C. Aliquots (1 mL) of the cultures were added to 100 mL LB medium with 80 µg/mL kanamycin at 37°C for 3 h to achieve the 0.5-0.8 OD_600_ value. Subsequently, IPTG (1 mM) was added to induce protein expression with incubation overnight at 18°C, 200 rpm. Simultaneously, *E. coli* BL21 (DE3) cells containing pET28a empty vector were used as negative control. *E. coli* cells were collected in 50 mL falcon tubes by centrifugation (13,000 x g at 4 °C for 30 min), and the pellets were resuspended into 1.5 mL lysis buffer (50 mM Tris, 20 mM imidazole, 500 mM NaCl, 10% glycerol, and 0.5% Tween 20; pH 7.5) containing a protease-inhibitor mix HP (1:100 v/v) (Serva Electrophoresis, Heidelberg, Germany) and benzonase (2 μL/10 mL lysis buffer) (Merck KGaA, Darmstadt, Germany), and then incubated on ice for 30 min before sonication (Sonoplus HD 2070, Bandelin, Berlin, Germany). The supernatant of the crude cell-free extract was collected by centrifugation (13,000 x g at 4 °C for 30 min) and then the histidine-tagged recombinant proteins were affinity-purified by transfer to Ni-NTA agarose resin (Qiagen) in 2 mL Eppendorf tubes, followed by mixing under circular rotation at 4 °C for 1 h. The Ni-NTA agarose resin was washed twice using wash buffer (50 mM Tris, 20 mM imidazole, 500 mM NaCl, and 10% glycerol; pH 7.5). Then the protein was eluted from the resin using elution buffer (50 mM Tris, 250 mM imidazole, 500 mM NaCl, and 10% glycerol; pH 7.5) and then changed into a potassium phosphate buffer (50 mM, and 10% glycerol; pH 6.5) using Amicon ultra-10 K centrifugal filter units (Merck KGaA). The purity of the eluted recombinant proteins was analyzed by SDS‐PAGE (BioRad, California, USA).

**Enzyme assays**

Specific activity and kinetic enzymatic assays were carried out in 250 µL volumes in UV-Star 96-well flat bottom microplates (Greiner Bio-One, Frickenhausen, Germany) and absorbances were measured using a multifunction microplate reader (Infinite M200, Tecan, Männedorf, Switzerland). Absorbance was measured for each well across a single row in 2 minutes (14 reaction cycles) at 274 nm with a minimal interval time at 25°C, and 4 no-enzyme controls were measured simultaneously. The linear reaction rates (measured over the 14 reaction cycles) were corrected for the non-enzymatic reaction rates and converted to molar substrate turnover using published extinction coefficients for ITCs (for 4MSOB-ITC (**1a**) Ɛ_274_ = 8.00 mM^-1^ cm^-1^; for A-ITC (**2a**) Ɛ_274_ = 7.45 mM^-1^ cm^-1^; for 2PE-ITC (**3a**) Ɛ_274_ = 8.89 mM^-1^ cm^-1^) [6]. Solutions containing GSH and ITCs (4MSOB-ITC (**1a**), A-ITC (**2a**), and 2PE-ITC (**3a**)) were freshly prepared before the assay, and dissolved in potassium phosphate buffer (100 mM, pH 6.5) and pure ethanol, respectively. A final concentration of 1 mM GSH, 0.4 mM ITCs (4% ethanol), and 1-2 µg BbGST protein in 250 µL potassium phosphate buffer (100 mM, pH 6.5) was used for specific activity assays. Specific activities reported here are the mean of 4 independent replicates. For kinetic assays, initial reaction rates (N=4) were measured at 6 to 7 concentrations (from 25 µM to 800 µM in 4% ethanol) of the rate-limiting substrate with 4 mM GSH concentration and 1 to 4 µg BbGST protein in 250 µL potassium phosphate buffer (100 mM, pH 6.5). Michaelis-Menten kinetic constants were determined using nonlinear regression in SigmaPlot 14.0.

**ITC formation by crude aphid protein**

To detect the GSL hydrolysis products of the cabbage aphid, a crude aphid protein extract was extracted and reacted with GSLs. Approximately 20 aphids fed on aliphatic GSL-deficient *myb28myb29* (KO) *A.* *thaliana* plants were pooled into 1.5 mL Eppendorf tubes as one sample (4 replicates per treatment). Samples were extracted in 200 μL ice-cold citric acid buffer (50 mM, and 10% glycerol; pH 4.1) with ceramic beads using a homogenizer for 3 min. Homogenized samples were centrifuged at 13,000 x g at 4°C for 20 min to separate undissolved particles. Clear supernatants were transferred to 1.5 ml Eppendorf tubes, and 5 μL of each sample were used to measure protein concentrations using Quick Start Bradford 1× Dye Reagent (Bio-Rad). Bovine serum albumin (Thermo Fisher Scientific) was used as an external standard.

Extracted protein (5 µg) from each sample was reacted with 4 mM GSL at 28 °C for 2 h in 100 µL citric acid buffer (50 mM, pH 4.1). 4MSOB-GSL (**1**), A-GSL (**2**), 2PE-GSL (**3**), and 2OH3But-GSL (**4**) were used for assays (Table S9). The reactions were stopped by adding 100 µL methanol (pH 3.0) and frozen at -20°C. Then they were centrifuged (13,000 x g at 4 °C for 30 min) and transferred to 2 mL vials. The hydrolysis products, including ITCs, were analyzed on an Agilent Technologies 1100 Series HPLC (Agilent Technologies) with a diode-array detector using a Nucleodur Sphinx RP column (250 × 4.6 mm × 5 mm, Macherey-Nagel). ITCs were detected at 240 nm and quantified by external standards (Table S9). Water and acetonitrile were employed as mobile phases A and B, respectively. The elution profile was: 0-10 min, 10-30% B; 10-10.10 min, 30-45% B; 10.10-25 min, 45-75% B; 25-25.1 min, 75-100% B; 25.1-27 min, 100% B; 27-27.1 min, 100-10% B; and 27.1-31 min, 10% B, at a flow rate of 1.0 mL/min.

**Non-targeted metabolomic analyses of GSL hydrolysis product metabolism in *B. bassiana* by UHPLC-qTOF-MS**

*B. bassiana* were incubated in PDB with 50 µM GSL hydrolysis products (4MSOB-ITC (**1a**), A-ITC (**2a**), 2PE-ITC (**3a**), or goitrin (**4b**) dissolved in 0.025% ethanol) for 24 h at 25°C and 180 rpm. *B. bassiana* incubated in PDB with solvent (0.025% ethanol) was used as negative control (N=4 for each treatment). *B. bassiana* was harvested by centrifugation (4,200 x g at 4 °C for 30 min) and freeze-dried samples were extracted in 500 µL 80% aqueous methanol.

The extracts were analyzed via UHPLC (Thermo Dionex Ultimate 3000, MA, USA) coupled to an qTOF MS system (Bruker timsTOF, Bremen, Germany). The UHPLC was equipped with a C18 reversed phase column (Zorbax Eclipse XDB-C18, 1.8 µm, 2.1 mm × 100 mm; Agilent Technologies, Böblingen, Germany) maintained at 25 °C and operated at 0.3 mL/min with a gradient flow of 0.1% aqueous formic acid (solvent A) and acetonitrile (solvent B) with the following profile: 5% B from 0–0.5 min, 5–60% B from 0.5 to 11 min, 60–100% B from 11–11.1 min and kept at 100% B until 12 min, then re-equilibrated at 5% B from 12.1–15 min. HRMS analyses were performed in positive ionization mode and automatic MS2 scans (“autoMS”) enabled. The source end plate offset was kept at 500 V and the capillary voltage at 4500 V, with the nebulizer gas at 2.8 bar, dry gas at 8 L/min and the drying temperature at 280 °C. Ion transfer was performed with a funnel 1 RF of 150 Vpp, funnel 2 RF of 200 Vpp, multipole RF of 50 Vpp, and a deflection delta of 70 V, with the quadrupole ion energy maintained at 4 eV (low mass 90 m/z). The mass scan range was 50–1500 m/z at an acquisition rate of 12 Hz. Collision energies were stepped in a 50:50 timing between a collision energy of 20 eV and 50 eV, respectively. At the beginning of each chromatographic analysis, 10 µL of a sodium formate-isopropanol solution (10 mM solution of NaOH in 50/50 (v/v%) isopropanol-water containing 0.2% formic acid) was injected into the dead volume of the sample injection for recalibration of the mass spectrometer using the expected cluster ion m/z values. Data were analyzed using the MetaboScape 5.3 software (Bruker, Bremen, Germany) and MetaboAnalyst 5.0 (https://dev.metaboanalyst.ca/). Automated peak picking and alignment were done within a retention time between 0.4 and 11 minutes, signal intensity ≥ 1500, maximum deviation of 2 ppm, and minimum occurrence in at least 4 samples. Feature groups, potentially representing single metabolites, were reduced to one bucket by the MetaboScape software to represent the respective metabolite in later analysis. Peaks deemed not present in specific samples were assigned an intensity value via k-nearest neighbors based on similar features for significant calculations. The data were normalized by sample weight and by log transformation base 10.

**Syntheses, purification, and NMR analyses of ITC conjugates**

The putative mercapturic acid pathway products 4MSOB-ITC-GSH (**1b**), 4MSOB-ITC-Cys (**1e**), and 4MSOB-ITC-NAC (**1f**) were confirmed by chromatographic and MS/MS comparison with commercially available standards (Table S9) via UHPLC-qTOFMS. The structures of other, non-commercially available putative ITC conjugates were confirmed after the compounds were chemically synthesized, chromatographically purified and analyzed by NMR. ITC conjugates were prepared synthetically as follows: 10 µL of the ITCs were mixed with 25 mg of GSH, Cys-Glu, Cys-Gly, Cys, or NAC in 10 mL H_2_O:ethanol (1:1; v:v) at room temperature for 48 h. The formation of targeted ITC-conjugates was confirmed using an HPLC (Agilent HP1100 series, Agilent Technologies) ion trap mass spectrometer (ESQUIRE-6000 system, Bruker) using a Nucleodur Sphinx RP column (250 × 4.6 mm × 5 µm, Macherey-Nagel). Aqueous formic acid (0.2%, solvent A) and acetonitrile were employed as mobile phases A and B, respectively. The elution profile was: 0-20 min, 10-50% B; 20-20.10 min, 50-100% B; 20.10-22 min, 100% B; 22-22.1 min, 100-10% B; and 22.1-26 min, 10% B at a flow rate of 1.0 mL/min. MS analyses were performed separately with positive and negative ionization and automatic MS2 scans (“autoMS”) enabled. The following parameters were used: capillary exit voltage, +117/-117 eV; capillary voltage, +3,000/-3,000V; nebulizer pressure, 35 psi; drying gas, 11 l min-1; gas temperature, 330°C. The mass scan range was 60–1000 *m*/*z*. The ethanol in the reaction mixtures was evaporated under N_2_ flow overnight. The ethanol-free mixture was passed through a SPE C18 column (5 g C18, 45 mL volume, Macherey-Nagel GmbH, Düren, Germany) previously conditioned using pure methanol and followed by MilliQ water. Subsequently, the SPE C18 column cartridge was washed with a stepwise gradient of aqueous methanol solutions (from 0%, 5%, 10%, 15%, 20%, 30%, 40% to 50 % methanol in water; v:v). 2PE-ITC-GSH (**3b**), 2PE-ITC-Cys-Glu (**3c**), 4MTB-ITC-Cys-Glu (**5c**), 4MTB-ITC-GSH (**5b**), 4MSOOB-ITC-GSH (**6b**), and A-ITC-NAC (**2f**) were collected in the 5% aqueous methanol fractions eluting from the SPE C18 cartridge; 4MSOOB-ITC-Cys (**6e**) was collected in the 10% aqueous methanol fraction; 4MSOB-ITC-Cys-Glu (**1c**) was collected in 20% aqueous methanol; and 2PE-ITC-Cys (**3e**) was collected in the 50% aqueous methanol fraction. These solutions were concentrated using a rotary evaporator R-114 (Büchi, Essen, Germany) before NMR analyses. 2PE-ITC-Cys-Glu (**3c**), 2PE-ITC-NAC (**3f**), 4MSOB-ITC-Cys-Gly (**1d**), A-ITC-GSH (**2b**), A-ITC-Cys-Glu (**2c**), and A-ITC-Cys (**2e**), which were not obtained in sufficient purities after C18 SPE, were further purified by repeated injection into an HPLC-UV system (Agilent Technologies 1100 Series HPLC (Agilent Technologies) equipped with a diode-array detector, using a Nucleodur Sphinx RP column (250 × 4.6 mm × 5 µm, Macherey-Nagel)). ITC conjugates were detected at 280 nm and collected. Aqueous formic acid (0.1%, solvent A) and acetonitrile were employed as mobile phases A and B, respectively. The elution profile was: 0-8 min, 5-45% B; 8-8.10 min, 45-100% B; 8.10-10 min, 100% B; 10-11.1 min, 100-5% B and 11.1-15 min, 5% B at a flow rate of 1.0 mL/min. The system was equipped with a fraction collector (Advantec SF-2110) and the collected conjugates were concentrated using a rotary evaporator. All purified ITC conjugates were analyzed by NMR spectroscopy. NMR spectra were measured on a 400 MHz Bruker Avance III HD spectrometer (Bruker Biospin GmbH, Rheinstetten, Germany). CD3OD and D2O were used as solvents. NMR spectra were referenced to the residual solvent signals at δH 3.31 and δC 49.0 for CD3OD and δH 4.79 for D2O. For spectrometer control and data processing, Bruker TopSpin ver. 3.6.1 software was used.

**Targeted HPLC-MS/MS analyses of 4MSOB-ITC (1a) metabolism**

*B. bassiana* cultures were incubated in PDB with 100 µM or 400 µM 4MSOB-ITC (**1a**) for 4 h, 12 h, or 24 h. *B. bassiana* was harvested by centrifugation (13,000 x g at 4 °C for 30 min), and a 5 µL aliquot of the PDB medium supernatant was collected. The harvested *B. bassiana* was freeze-dried, weighed, and homogenized. *B. bassiana* and the medium metabolites (N=5 for each treatment) were extracted in 500 µL 80% aqueous methanol. To detect *B. bassiana* metabolism of the 4MSOB-GSL (**1**) sequestered in the cabbage aphids fed on *A. thaliana* wild-type plants, aphids infected and non-infected by *B. bassiana* were collected on the 5^th^ day post infection from host plants. The harvested samples (N=5 for each treatment) were extracted in 350 µL 80% aqueous methanol for further chemical analyses.

The targeted analyses of 4MSOB-ITC (**1a**), and its conjugates were performed on an Agilent HP1260 series HPLC instrument (Agilent Technologies, Böblingen, Germany) coupled to an API5000 tandem mass spectrometer (Applied Biosystems, Darmstadt, Germany). 4MSOB–ITC (**1a**) and its conjugates were separated on an Agilent Zorbax Eclipse XDB-C18 column (50 × 4.6 mm× 1.8 μm, Agilent Technologies, Wilmington, DE, USA) with mobile phase A (0.05% formic acid in milliQ water) and mobile phase B (acetonitrile). The elution profile was: 0–0.5 min, 3-15% B; 0.5– 2.5 min, 15-85% B; 2.5–2.6 min, 85-100% B; 2.6– 3.5 min, 100% B,3.5– 3.6 min, 100-3% B 3.6– 6 min, 3% B at a flow rate of 1.1 mL/min. 4MSOB-GSL was analyzed with the same LC-MS system using a Nucleodur Sphinx RP column (250 × 4.6 mm × 5 μm, Macherey-Nagel) with mobile phase A (0.2 % formic acid in milliQ water) and mobile phase B (acetonitrile). The elution profile was: 0–1 min, 1.5% B; 1–6 min, 1.5-5% B; 6– 8 min, 5-7% B; 8–9 min, 7-8.4% B; 9–9.1 min, 8.4-100% B; 9.1–10min, 100% B; 10–10.1 min, 100-1.5% B; 10.1–14 min, 1.5% B, at a flow rate of 1.0 mL/min. Detection of each compound was achieved by multiple reaction monitoring (MRM) of specific parent to product ion conversions for each compound. Parameters are described in Table S10. Analyst 1.5 software (Applied Biosystems) was used for data acquisition and processing. Quantification of individual compounds was achieved by external calibration curves, with the origins of the external standards listed in Table S9.

**Amino acid content measurement**

Amino acids, GSH and GSSG were quantified after *B. bassiana* was shaken for 24 h in PDB with 4MSOB-ITC (**1a**), A-ITC (**2a**), 2PE-ITC (**3a**), goitrin (**4b**), or solvent control (0.025% ethanol). Cysteine and glycine were measured as their FMOC derivatives, while the remaining proteinogenic amino acids were analyzed directly without derivatization. The analyses of GSH and GSSG, and FMOC-derived amino acids were performed on an Agilent HP1260 Series instrument coupled to an API5000 tandem mass spectrometer. An aliquot of the aqueous extract (10 µL) was mixed with 90 µL ^13^C- and ^15^N- labeled amino acid standard solution (10 µg/mL; algal amino acids ^13^C, ^15^N, Isotec, Miamisburg, US) for the measurement of the remaining amino acids, which were quantified using an Agilent 1260 HPLC coupled to an API6500 tandem mass spectrometer (Sciex).

The FMOC-derivatization was carried out as follows: 10 µL of the aqueous extract were mixed with 90 µL ^13^C- and ^15^N- labeled amino acid standard solution (20 µg/mL; algal amino acids ^13^C, ^15^N, Isotec, Miamisburg, US) and 100 µL borate buffer (0.8 M, pH 10). FMOC-Cl (200 µL of 30 mM solution in acetonitrile) was added and the reaction was gently mixed and incubated for 5 min. Excess FMOC-Cl was removed by extraction with hexane (800 µL). After phase separation, 100 µL of the bottom aqueous phase were carefully collected and transferred to a 2 mL vial with insert. The analyses of GSH, GSSG, and FMOC-derivatized amino acids were performed on an Agilent HP1260 series instrument (Agilent Technologies) coupled to an API5000 tandem mass spectrometer (Applied Biosystems). GSH and GSSG were analyzed with a Nucleodur Sphinx RP column (250 × 4.6 mm × 5 µm, Macherey-Nagel) with mobile phase A (0.2 % formic acid in milliQ water) and mobile phase B (acetonitrile). The elution profile was: 0–3.5 min, 2% B; 3.5-9 min, 2-35% B 9–9.1 min, 35-100% B; 9.1–11 min, 100% B; 11– 11.1 min, 100-2% B; 11.1– 15 min, 2% B at a flow rate of 1.0 mL/min. FMOC derivatives were analyzed with an Agilent Zorbax Eclipse XDB-C18 column (50 × 4.6 mm× 1.8 μm, Agilent Technologies) with mobile phase A (0.05 % formic acid in milliQ water) and mobile phase B (acetonitrile). The elution profile was: 0–0.5 min, 10% B; 0.5-4.5 min, 10-90% B; 4.5–6 min, 90-100% B; 6–6.5 min, 100% B; 6.5– 6.51 min, 100-10% B; 6.51– 9 min, 10% B at a flow rate of 1.1 mL/min. The electrospray parameters for both LC-MS methods were: ionspray voltage -4.5 keV, turbo gas temperature 700°C, curtain gas 35 psi, nebulizing gas 70 psi, and heating gas 70 psi. Detection in the negative ionization mode was achieved in multiple-reaction-monitoring mode (MRM), for details see Table S10.

Aliquots (10 µL) of the aqueous extracts were mixed with 90 µL ^13^C- and ^15^N- labeled amino acid standard solution (10 µg/mL) for the remaining amino acid measurements, which were performed using an Agilent 1260 HPLC system (Agilent Technologies) coupled to an API6500 tandem mass spectrometer (Sciex). The HPLC was equipped with an Agilent Zorbax Eclipse XDB-C18 column (50 × 4.6 mm× 1.8 μm, Agilent Technologies) with mobile phase A (0.05 % formic acid in milliQ water) and mobile phase B (acetonitrile). The elution profile was: 0–1 min, 3% B; 1–2.7 min, 3-100% B; 2.7–3 min, 100% B; 3–3.1 min, 100-3% B; 3.1–6 min, 3% B, at a flow rate of 1.1 mL/min. The electrospray parameters were: ionspray voltage 5 keV, turbo gas temperature 650°C, curtain gas 40 psi, nebulizing gas 70 psi, and heating gas 70 psi. Detection of each compound was achieved by multiple reaction monitoring (MRM) of specific parent to product ion conversions for each compound, parameters are described in (Table S10). Analyst 1.5 software (Applied Biosystems) was used for data acquisition and processing. Quantification of individual compounds was achieved using internal standards (amino acids) and external calibration curves (GSH and GSSG); the origins of the standards are listed in Table S9.

**HPLC-UV determination of 4MSOB-ITC (1a), 4MTB-ITC (5a), and 4MSOOB-ITC (6a) in commercial stocks**

The contents of 4MTB-ITC (**5a**) and 4MSOOB-ITC (**6a**) in the commercial 4MSOB-ITC (**1a**) standard used were determined. 4MSOB-ITC (**1a**) (0.4 mM) was prepared in methanol and analyzed on an Agilent Technologies 1100 Series HPLC (Agilent Technologies) with a diode-array detector using a Nucleodur Sphinx RP column (250 × 4.6 mm × 5 mm, Macherey-Nagel). Commercial 4MTB-ITC (**5a**) (0.4mM), and 4MSOOB-ITC (**6a**) (0.2 mM) in methanol were used as standards. The chromatographic method for ITC detection by HPLC-UV (at 240nm) was as above (see ”ITC formation by crude aphid protein”).

**Effects of ITCs on *B. bassiana* growth**

The influence of GSL hydrolysis products on *B. bassiana* growth was monitored by growing the fungus on PDA with increasing concentrations (0 µM to 360 µM) of 4MSOB-ITC (**1a**), A-ITC (**2a**), 2PE-ITC (**3a**), goitrin (**4b**), or solvent (negative control), and the growth of the fungal colonies were measured 8 days after inoculation. To examine the roles of GSH in 4MSOB-ITC (**1a**) detoxification in *B. bassiana*, fungal growth was measured on basic medium (Czapek dox agar, Sigma-Aldrich, Munich, Germany) with 2mM GSH, 4 mM GSH, or without GSH, and a gradient of increasing concentrations of 4MSOB-ITC (**1a**) from 0 µM to 1000 µM. *B. bassiana* conidia (1 µL of a solution containing 10^8^ conidia per mL) were added in the middle of the prepared plates. The growth of the fungal colonies was measured 9 days after inoculation. The diameters of the fungal colonies were measured from two different directions and the averages used for the colony area calculation. To examine the roles of GSH in detoxification of different ITCs, *B. bassiana* growth was measured on basic medium with 60 µM ITCs and increasing concentrations of GSH (0 mM to 2 mM. N=3 replicates for each treatment), and the growth of the fungal colonies was measured 9 days after inoculation.

**Statistical analyses**

Data were analyzed using the R statistics package version 3.6.1 and figures were created using Origin 2019 and Adobe Illustrator CS5. All data were checked for statistical prerequisites such as homogeneity of variances and normality. Significance differences were determined by Tukey HSD tests in conjugation with one/two-way ANOVA, two-tailed t tests, Mann-Whitney U tests, and Dunn’s post hoc tests in conjugation with Kruskal-Wallis test according to the data distribution and the number group for comparison. The specific analyses used are listed in the figures and figure legends. Letters in the graphs represent differences at *P*≤ 0.05. n.s, *P*≥ 0.05; *, *P*≤ 0.05; **, *P*≤ 0.01; ***, *P*≤ 0.001.

**References**

1. Sønderby IE, Hansen BG, Bjarnholt N, Ticconi C, Halkier BA, and Kliebenstein DJ. A systems biology approach identifies a R2R3 MYB gene subfamily with distinct and overlapping functions in regulation of aliphatic glucosinolates. PLoS One. 2007; 2(12):e1322.

2. Kim S, Lee SJ, Nai YS, Yu JS, Lee MR, Yang YT, et al. Characterization of T-DNA insertion mutants with decreased virulence in the entomopathogenic fungus *Beauveria bassiana* JEF-007. Appl Microbiol Biotechnol. 2016; 100(20):8889-8900.

3. Brown PD, Tokuhisa JG, Reichelt M, and Gershenzon J. Variation of glucosinolate accumulation among different organs and developmental stages of *Arabidopsis thaliana*. Phytochem. 2003; 62(3):471-481.

4. Graser G, Schneider B, Oldham NJ, and Gershenzon J. The methionine chain elongation pathway in the biosynthesis of glucosinolates in *Eruca sativa* (Brassicaceae). Arch Biochem Biophys. 2000; 378(2):411-419.

5. Burow M, Müller R, Gershenzon J, and Wittstock U. Altered glucosinolate hydrolysis in genetically engineered *Arabidopsis thaliana* and its influence on the larval development of *Spodoptera littoralis*. J Chem Ecol. 2006; 32(11):2333-2349.

6. Kolm RH, Danielson UH, Zhang Y, Talalay P, and Mannervik B. Isothiocyanates as substrates for human glutathione transferases: structure-activity studies. Biochem. 1995; 311(2):453-459.
