# Supplementary material for: Metabolism of plant-derived toxins from its insect host increases the success of the entomopathogenic fungus Beauveria bassiana
Source: ISME J. 2023 Jul 21;17(10):1693–704. doi: 10.1038/s41396-023-01480-3 (PMC10504261; doi:10.1038/s41396-023-01480-3)

Standards of 4MSOB-ITC-conjugates

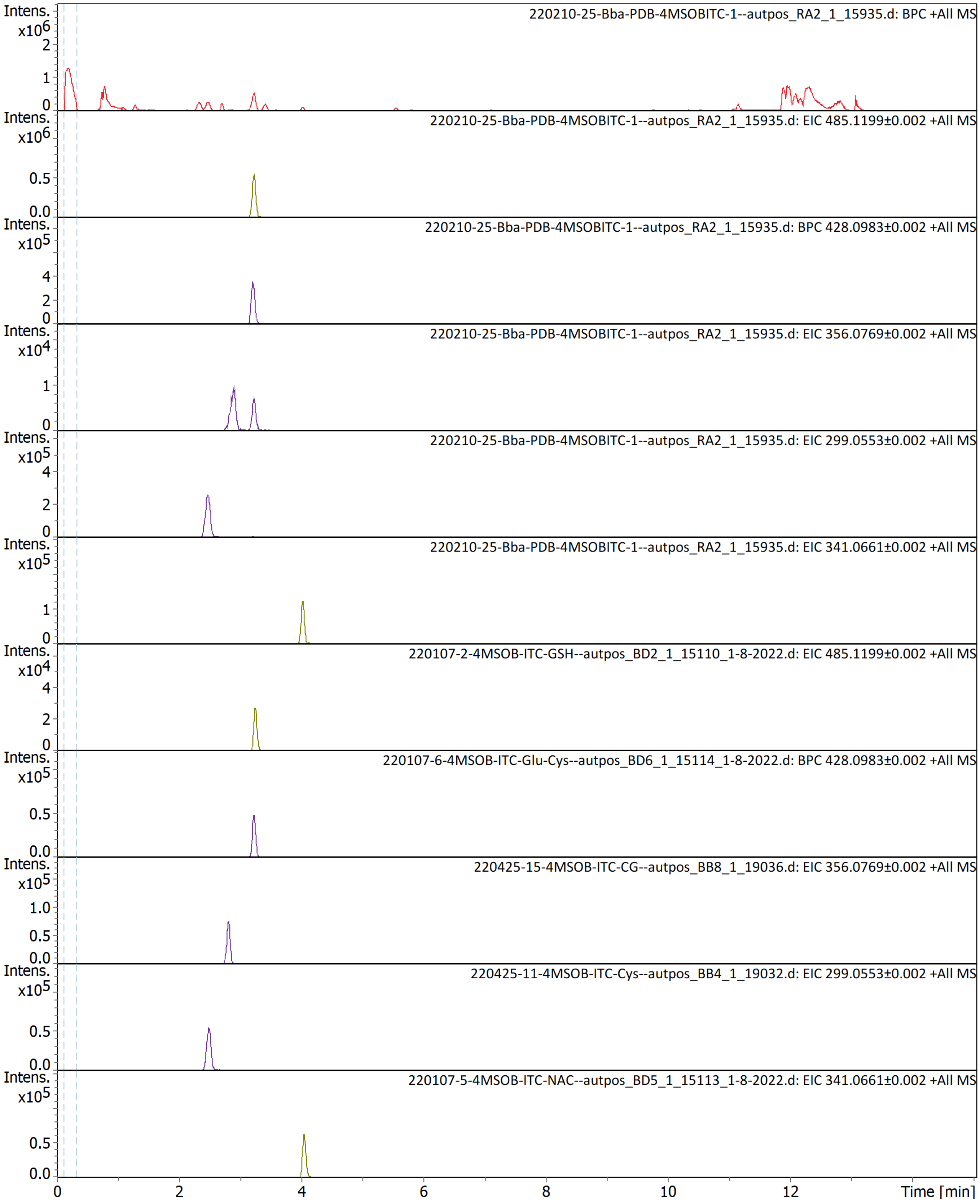

Bruker Compass DataAnalysis 5.3

Standards of 4MTB-ITC-conjugates

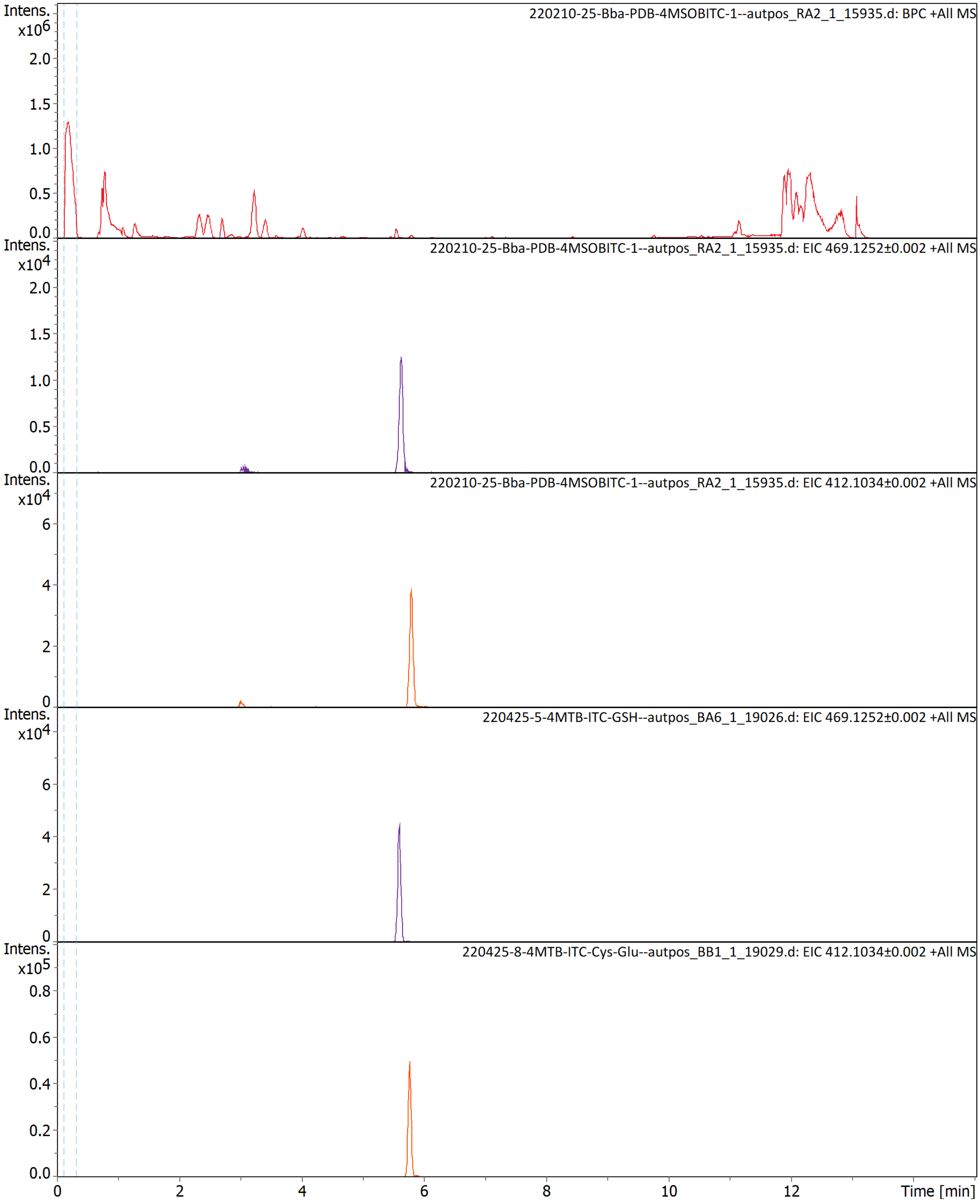

Standards of Erysolin-conjugates

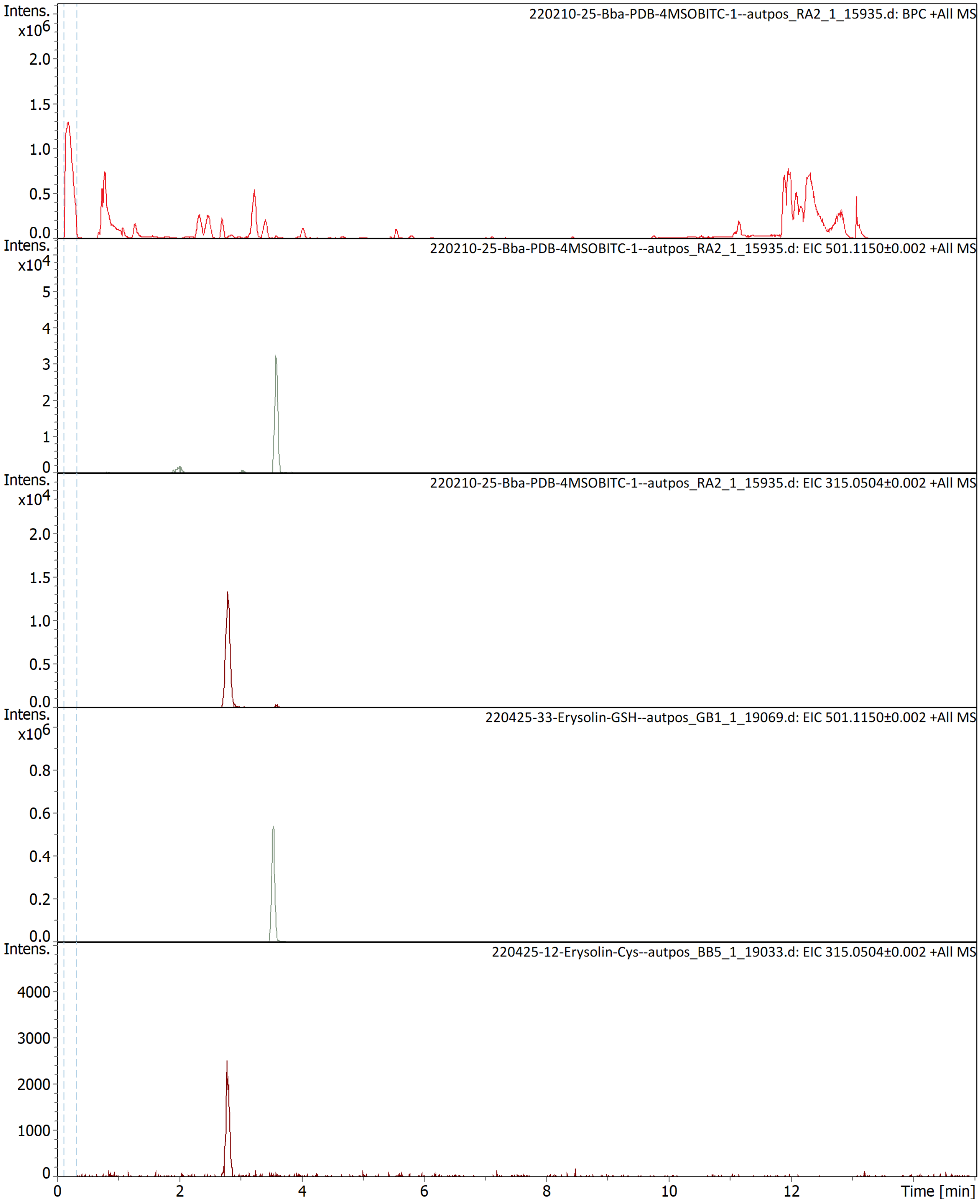

Bruker Compass DataAnalysis 5.3

Standards of Allyl-ITC-conjugates

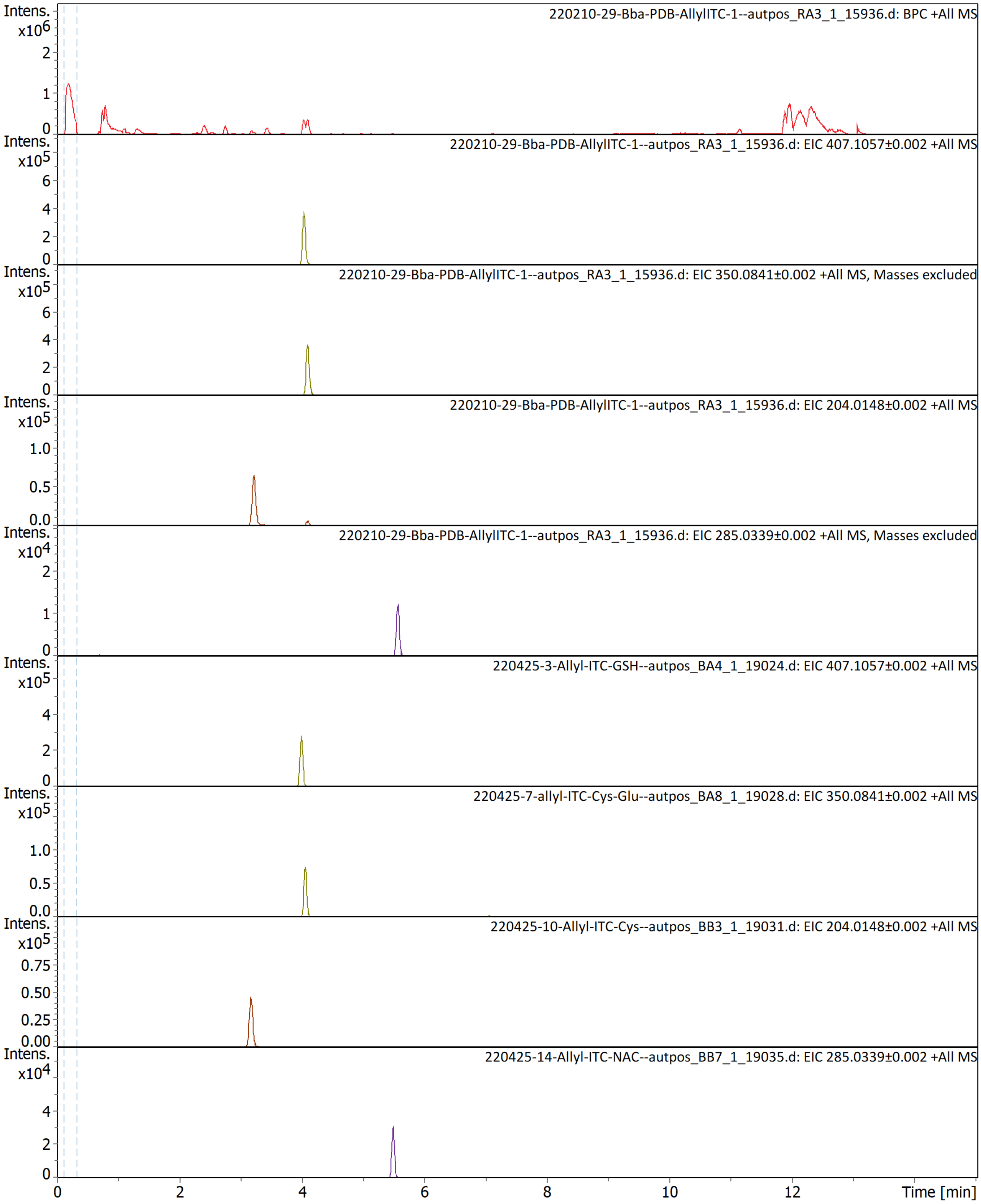

Standards of 2PE-ITC-conjugates

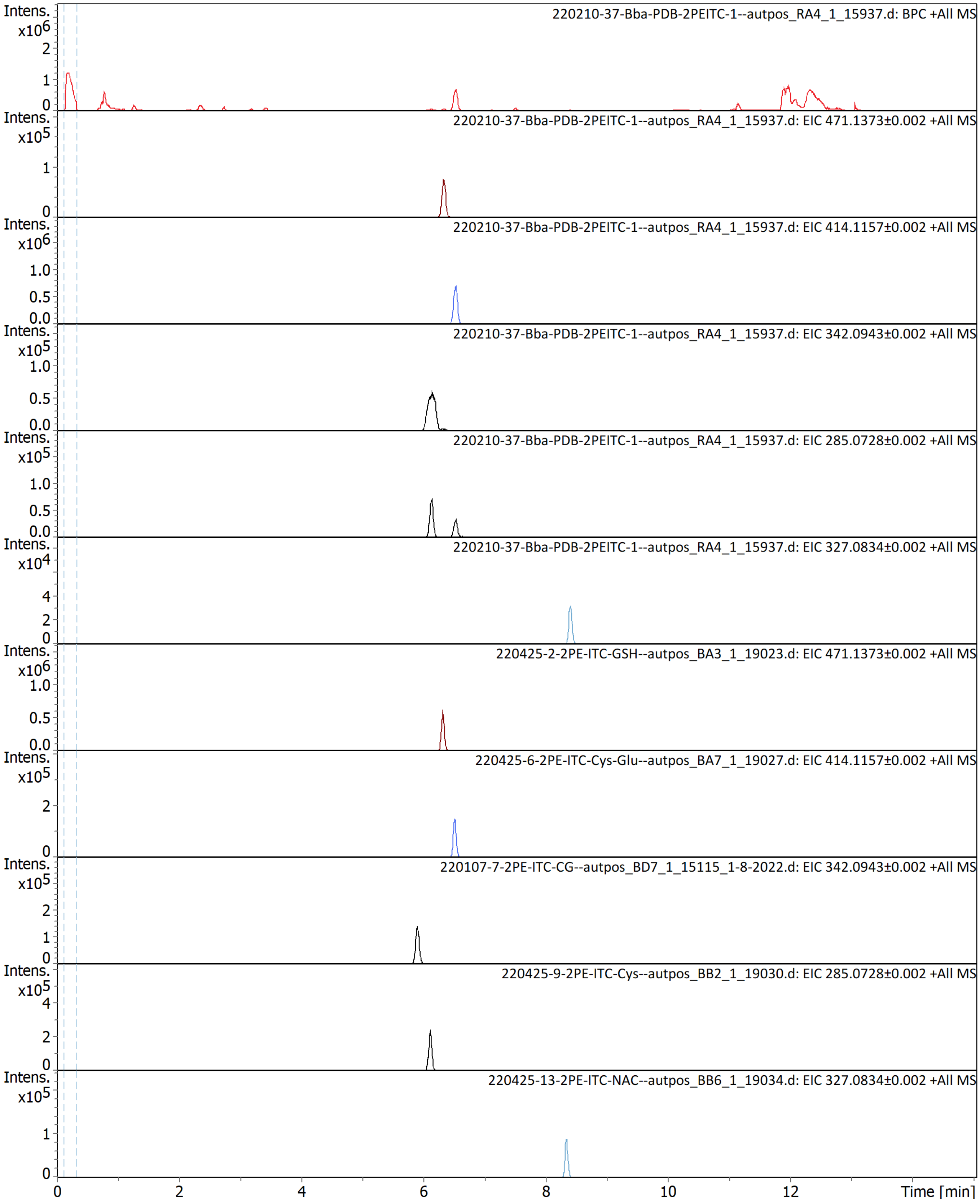

Bruker Compass DataAnalysis 5.3

4MSOB-ITC-GSH vs 4MSOB-ITC-Cys-Gly-GABA

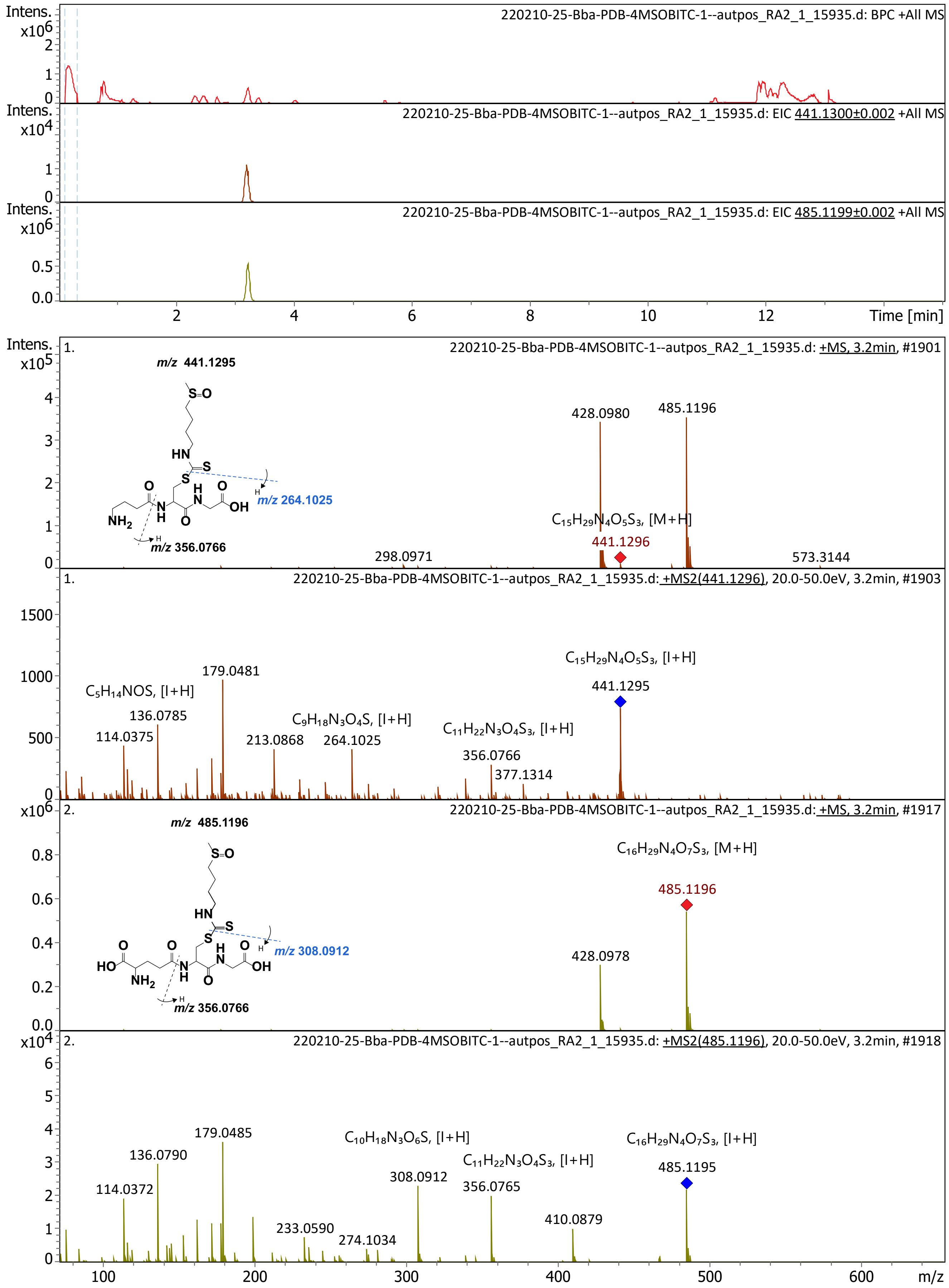

Allyl-ITC-GSH vs Allyl-ITC-Cys-Gly-PA

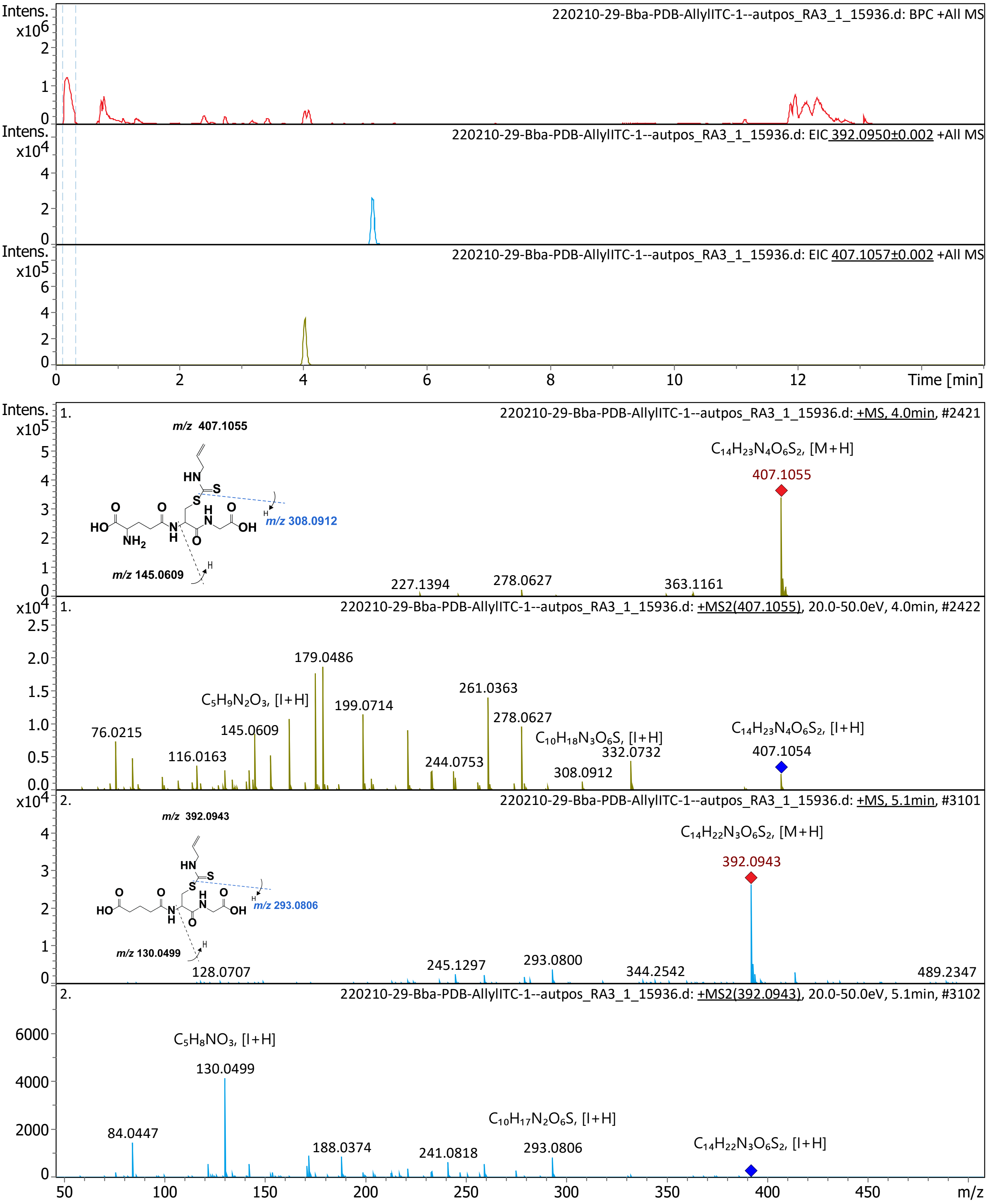

2PE-ITC-Cys-Glu vs 2PE-ITC-Cys-GABA

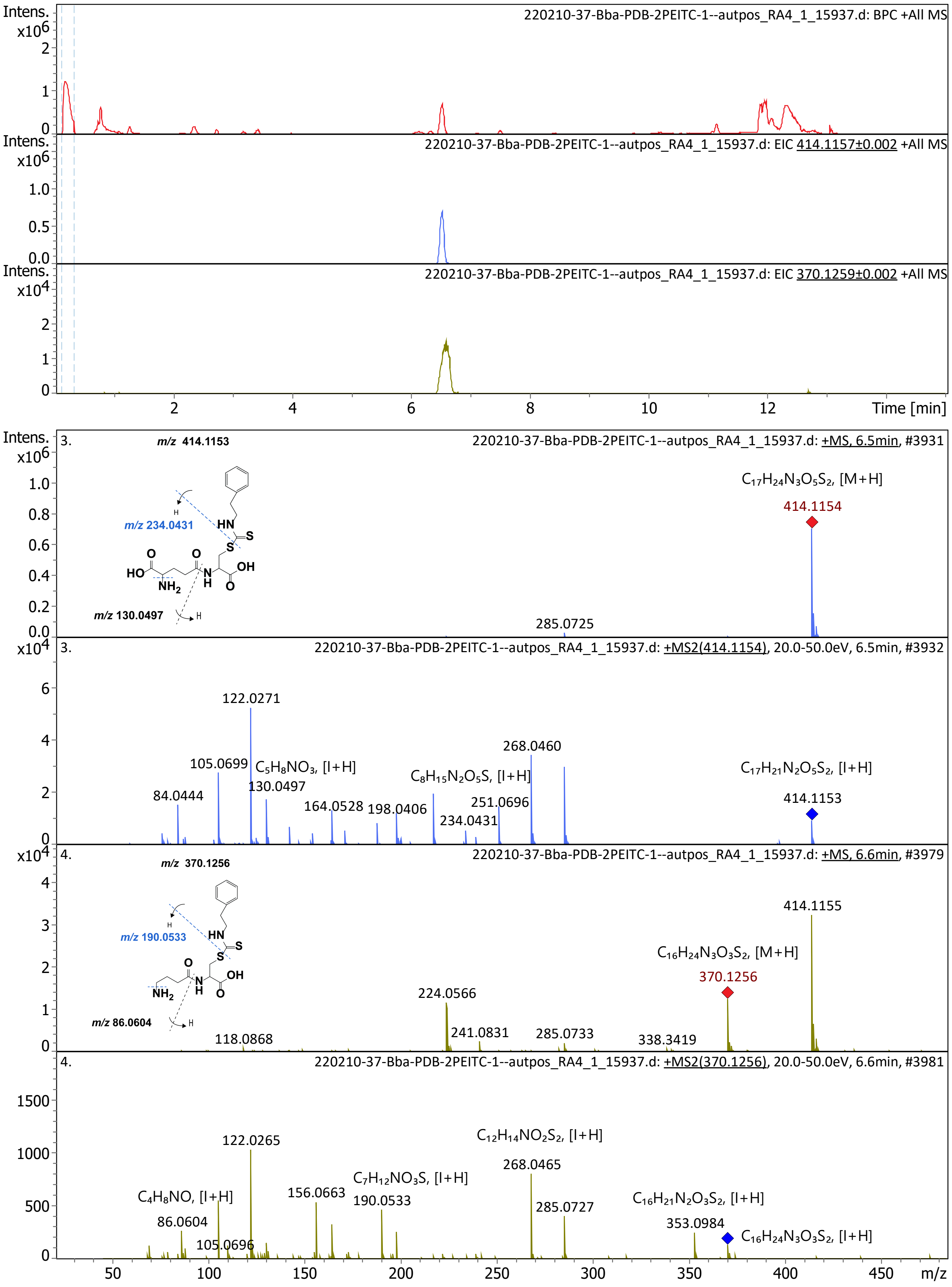

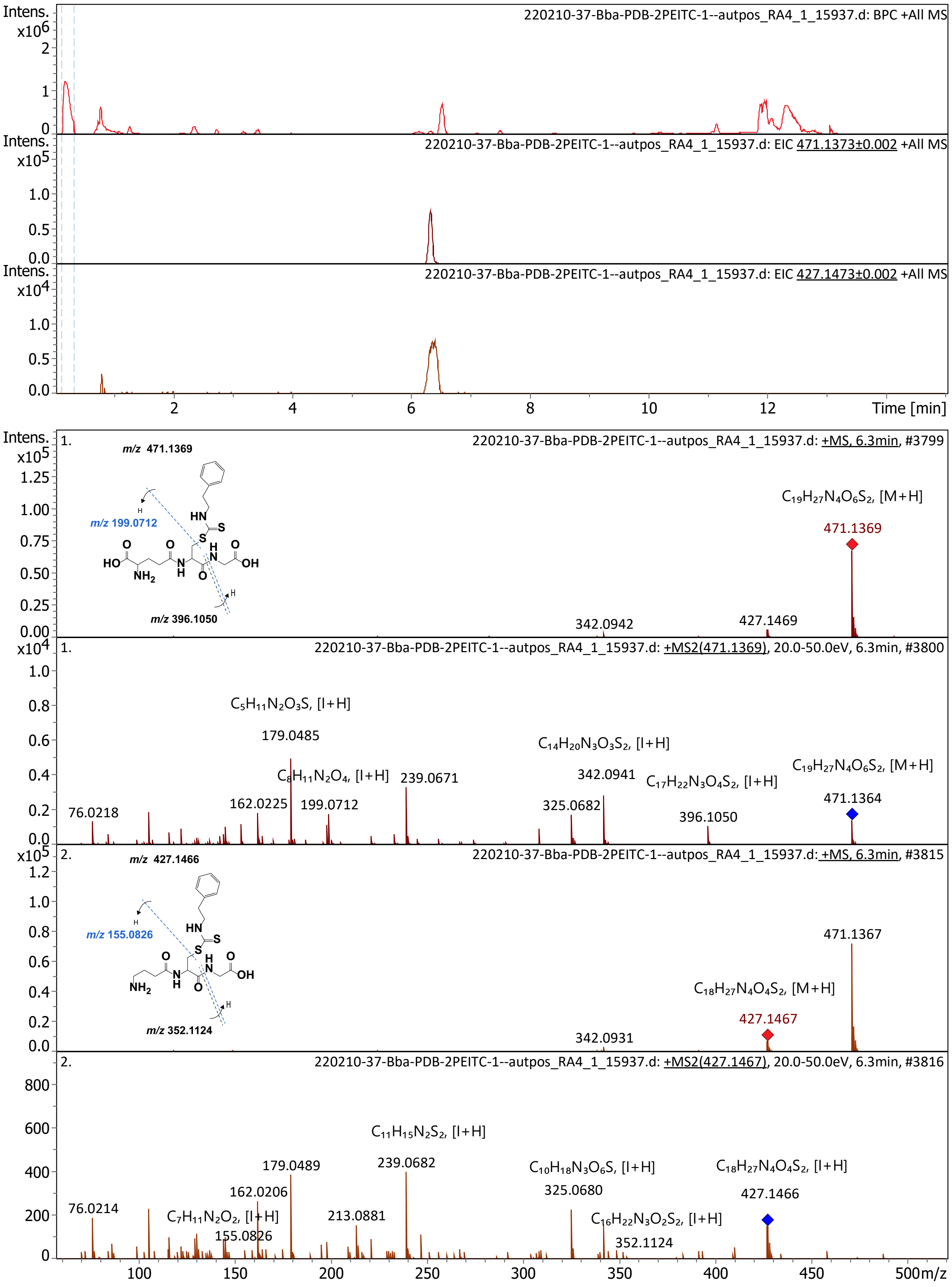
220210-37-Bba-PDB-2PEITC-1--autpos_RA4_1_15937.d: BPC +All MS
220210-37-Bba-PDB-2PEITC-1--autpos_RA4_1_15937.d: EIC 471.1373±0.002 +All MS
220210-37-Bba-PDB-2PEITC-1--autpos_RA4_1_15937.d: EIC 427.1473±0.002 +All MS
Intens.
Time [min]
1. 220210-37-Bba-PDB-2PEITC-1--autpos_RA4_1_15937.d: +MS, 6.3min, #3799
m/z 471.1369
m/z 199.0712
m/z 396.1050
C19H27N4O6S2, [M+H]
471.1369
342.0942
427.1469
1. 220210-37-Bba-PDB-2PEITC-1--autpos_RA4_1_15937.d: +MS2(471.1369), 20.0-50.0eV, 6.3min, #3800
C5H11N2O3S, [I+H]
179.0485
C8H11N2O4, [I+H]
199.0712
162.0225
239.0671
76.0218
C14H20N3O3S2, [I+H]
342.0941
325.0682
C17H22N3O4S2, [I+H]
396.1050
C19H27N4O6S2, [M+H]
471.1364
2. 220210-37-Bba-PDB-2PEITC-1--autpos_RA4_1_15937.d: +MS, 6.3min, #3815
m/z 427.1466
m/z 155.0826
m/z 352.1124
471.1367
C18H27N4O4S2, [M+H]
427.1467
342.0931
2. 220210-37-Bba-PDB-2PEITC-1--autpos_RA4_1_15937.d: +MS2(427.1467), 20.0-50.0eV, 6.3min, #3816
C11H15N2S2, [I+H]
239.0682
179.0489
162.0206
76.0214
C7H11N2O2, [I+H]
155.0826
213.0881
C10H18N3O6S, [I+H]
325.0680
C16H22N3O2S2, [I+H]
352.1124
C18H27N4O4S2, [I+H]
427.1466
m/z

2PE-ITC-GSH vs 2PE-ITC-Cys-Gly-PA

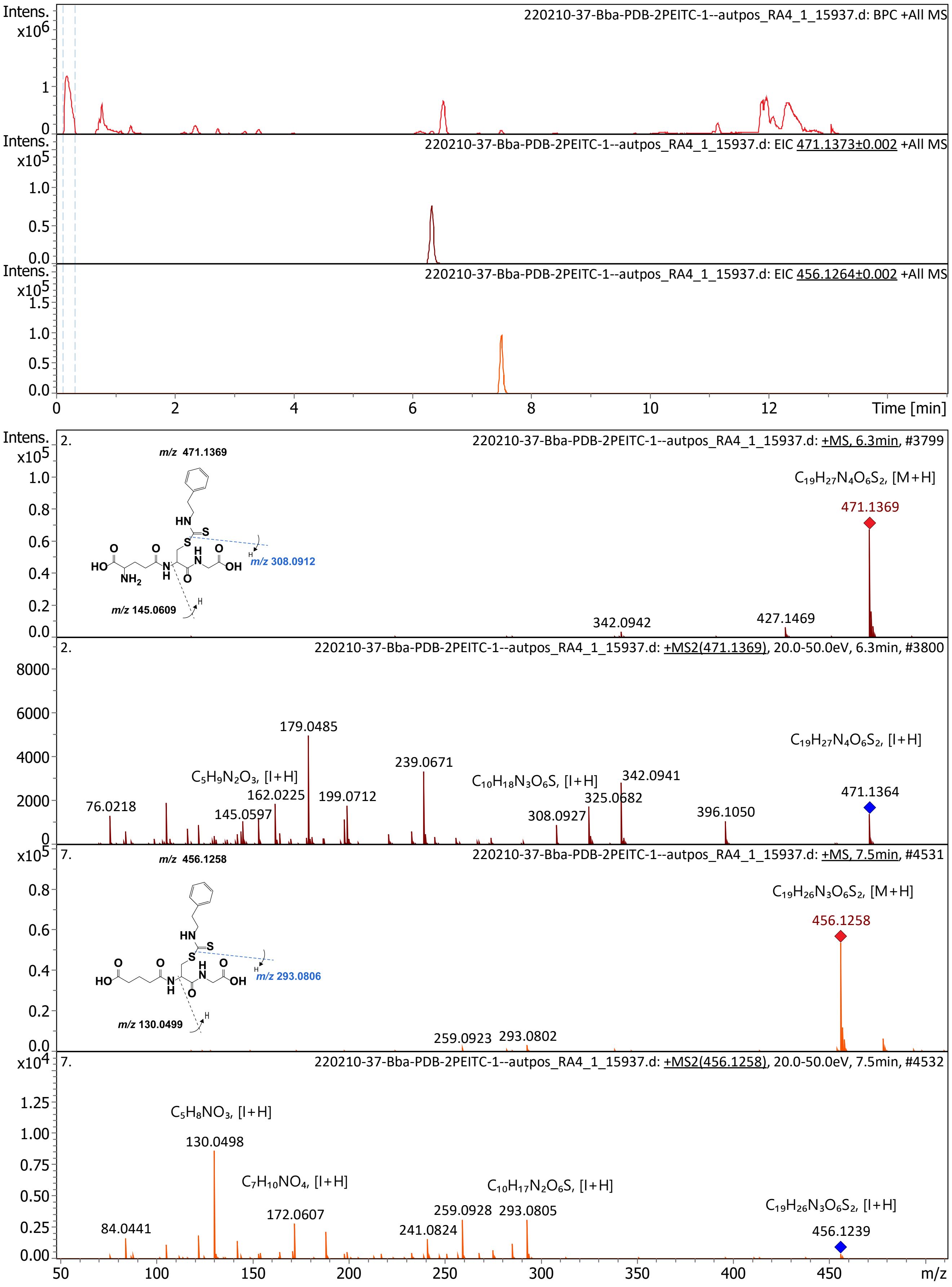

Supplement: Supplementary file 4 [file 41396_2023_1480_MOESM4_ESM.pdf]
